# Identification of resistance sources and genomic regions regulating Septoria tritici blotch resistance in South Asian bread wheat germplasm

**DOI:** 10.1002/tpg2.20531

**Published:** 2024-11-27

**Authors:** Manjeet Kumar, Xinyao He, Sudhir Navathe, Umesh Kamble, Madhu Patial, Pawan Kumar Singh

**Affiliations:** ^1^ ICAR‐Indian Agricultural Research Institute New Delhi India; ^2^ International Maize and Wheat Improvement Centre (CIMMYT) Apedo Mexico DF Mexico; ^3^ Agharkar Research Institute Pune India; ^4^ ICAR‐Indian Institute of Wheat and Barley Research Karnal India; ^5^ ICAR‐Indian Agricultural Research Institute, Regional Station Shimla India

## Abstract

The Septoria tritici blotch (STB) [*Zymoseptoria tritici* (Desm.)] of wheat (*Triticum aestivum* L.) is characterized by its polycyclic and hemibiotrophic nature. It is one of the most dangerous diseases affecting wheat production worldwide. Durable resistance is largely decided by the combined effect of several quantitative trait loci (QTLs) having a minor effect. Currently, STB is not important in South Asia. However, STB expanding and wider adaptability, changing climatic conditions, and agronomic practices can create a situation of concern. Therefore, dissection of the genetic architecture of adult‐plant resistance with genome‐wide association mapping and selection of resistant sources for adult plant STB resistance were carried out on a panel of South Asian germplasm. We discovered the 91 quantitative trait nucleotides (QTNs) associated with STB resistance; 23 QTNs were repetitive across the different years and models. Many of these QTNs could differentiate the mapping panel into resistant versus susceptible groups and were linked to candidate genes related to disease resistance functions within linkage disequilibrium blocks. The repetitive QTNs, namely, Q.CIM.stb.2DL.2, Q.CIM.stb_dh.2DL.3, Q.CIM.stb.2AL.5, and Q.CIM.stb.7BL.1, may be novel due to the absence of co‐localization of previously reported QTLs, meta‐quantitative trait loci, and STB genes. There was a perfect negative correlation between the stacking of favorable alleles and STB susceptibility, and STB resistance response was improved by ∼50% with the stacking of ≥60% favorable alleles. The genotypes, namely, CIM20, CIM56, CIM57, CIM18, CIM44, WK2395, and K1317, could be used as resistant sources in wheat breeding programs. Therefore, this study could aid in designing the breeding programs for STB resistance before the onset of the alarming situation of STB in South Asia.

## Core Ideas

- Resistant germplasm identification to Septoria tritici blotch of wheat was investigated.
- The authors identified molecular markers/genomic regions associated with Septoria tritici blotch resistance.
- A genome wide association study was used to analyze Septoria tritici blotch resistance in South Asian spring wheat panel.

**Abbreviations**
ANOVA: analysis of variance
APR: adult‐plant resistance
AUDPC: area under disease progressive curve
BLINK: Bayesian‐information and linkage‐disequilibrium iteratively nested keyway
BLUP: best linear unbiased prediction
CG: candidate gene
CIM: CIMMYT
DH: days to heading
FarmCPU: fixed and random model circulating probability unification
gBLUP: genomic best linear unbiased prediction
GEBV: genomic estimated breeding value
GWAS: genome wide association study
KASP: kompetitive allele‐specific primer
LD: linkage disequilibrium
MLM: mixed linear model
MQTL: meta‐quantitative trait locus
MTA: marker‐trait association
PH: plant height
QTL: quantitative trait locus
QTN: quantitative trait nucleotide
STB: Septoria tritici blotch
Stb: Septoria tritici blotch
STB_POOL: Septoria tritici blotch infection across years
STB2019: Septoria tritici blotch infection in year 2019
STB2020: Septoria tritici blotch infection in year 2020
STB2021: Septoria tritici blotch infection in year 2021

## 1 INTRODUCTION

Septoria tritici blotch (STB) is a foliar disease caused by the ascomycete's fungus *Zymoseptoria tritici* (Desm.) and considered one of the most important wheat diseases across the world (Yang et al., 2022). *Zymoseptoria* *tritici* is a polycyclic hemibiotrophic fungus that completes several life cycles during the growth cycle of wheat (*Triticum aestivum* L.). This disease impacts wheat production in Europe, the Mediterranean region, Africa, the Americas, and Australia (Dean et al., 2012; Fones & Gurr, 2015; Kosina et al., 2007), where, under favorable environmental conditions like high humidity and mild temperature, about 30%–50% yield losses could happen (Duveiller et al., 2007; Eyal, 1999; Eyal et al., 1987). There is no major concern about STB in South Asian countries, and reports on STB presence are unavailable. However, the chance for STB to become more important in South Asian countries might increase considering the below factors: (1) the changing climatic conditions, (2) the possible appearance of newly adapted pathogenic races, (3) wide cultivation of susceptible varieties, (4) large‐scale adoption of conservation agriculture with residue retention, and (5) transboundary movement of the pathogen like wheat blast (Singh et al., 2021). Various factors affect STB intensity, like wheat variety, plant population, weed density, planting date, growth stage, and soil type (Hailemariam et al., 2020). Host resistance and fungicide application are the two most used approaches to limit STB‐inflicted yield losses. However, fungicides are not environmentally friendly, and fungicide resistance has been reported (Cools & Fraaije, 2013; Torriani et al., 2015). Therefore, moderate to high levels of host resistance should be part of the fungicide‐based integrated disease management system (Yang et al., 2022). Identifying and utilizing genomic regions controlling STB resistance is of utmost importance to developing STB‐resistant wheat varieties based on a molecular breeding approach. STB resistance is governed by both race‐specific resistance with major genes (McCartney et al., 2002) and non‐race‐specific resistance with numerous small‐effect quantitative trait loci (QTLs) (Karisto et al., 2018). Over the past, 24 resistance genes (R) have been identified in wheat for STB resistance, including 12 isolate‐specific genes and 12 non‐isolate‐specific genes (Yang et al., 2018, 2022). Most known major *Stb* genes, however, have broken down in Europe (Brown et al., 2015), and likewise, *Stb4*, *Stb6*, *Stb2*/*11*/*WW*, and *Stb18* have been overcome in Australia (Yang et al., 2022). In contrast, QTLs with small‐to‐moderate effects on STB have shown more durable resistance and weaker specificity (Goudemand et al., 2013; Brown et al., 2015). On the basis of seven biparental mapping populations, 115 QTLs were redefined into 27 meta‐quantitative trait loci (MQTLs) for STB resistance at the seedling and/or adult stages (Goudemand et al., 2013). Besides biparental QTL mapping, genome‐wide association (GWAS) study is another popular approach that takes advantage of historic recombination events and often has a higher resolution for mapping QTL. Also, it is more likely to detect greater diversity of alleles faster and more efficiently (Yu & Buckler, 2006). In this regard, numerous GWAS for STB resistance at the adult plant stage have been conducted and reported (Alemu et al., 2021; Arraiano & Brown, 2017; Kidane et al., 2017; Louriki et al., 2021; Mahboubi et al., 2022; Muqaddasi et al., 2019; Riaz et al., 2020; Vagndorf et al., 2017; Würschum et al., 2017; Yates et al., 2019). Examples include QStb.NS‐2A associated with adult‐plant resistance (APR) (Vagndorf et al., 2017), QTL‐3 on the control of necrosis lesions (Yates et al., 2019), and QStb.wai.6A.1 linked with multi‐stage resistance (Yang et al., 2022). Presumably, over the last 30 years, progress in developing the STB‐resistance lines resulted from the gradual accumulation of minor genes (Torriani et al., 2015), and effectiveness might be durable (Krenz et al., 2008). Alternatively, combining *Stb* genes with resistance QTLs can effectively achieve durable resistance. A molecular breeding approach involving identifying and deploying major genes and QTLs is a good approach for selection.

Notably, late heading and tall height are responsible for disease escape by limiting the inoculum spread (Gerard et al., 2017; Riaz et al., 2020; Simón et al., 2004; Yang et al., 2022). However, late flowering and tall height generally make the genotypes prone to terminal heat stress and lodging. Hence, these traits are undesirable, especially for spring wheat under the timely sown condition in South Asian countries. Therefore, identifying and characterizing genomic regions governing the true resistance (no disease escape) are crucial in breeding for STB resistance by keeping the limits on plant height (PH) and days to heading (DH). However, reports of genomic regions governing STB resistance and resistant germplasm in South Asian wheat germplasm are currently unavailable. Therefore, this GWAS based on 173 South Asian genotypes with a 15,000 single nucleotide polymorphisms (SNPs) array was planned to identify quantitative trait nucleotides (QTNs) associated with STB resistance that could be subsequently utilized directly in local breeding programs without compromising the chances of introduction of undesirable traits like late flowering from winter wheat.

## 2 MATERIALS AND METHODS

### 2.1 Genome‐wide association mapping panel and Septoria tritici blotch phenotyping

A set of 173 bread wheat lines of spring type, which includes the germplasm of International Maize and Wheat Improvement Center (CIMMYT)‐Mexico (CIM), India (IND), Bangladesh, and Nepal (NPL) origin with great diversity in their pedigree, were evaluated for STB resistance under field conditions (Table S1). The experiment was conducted as a summer nursery for three consecutive years, that is, 2019, 2020, and 2021, respectively, at the Dr. Sanjaya Rajaram Experimentation Station, CIMMYT Toluca, which has the coordinates of 19.2 N, 99.6 W, and 2580 MSL. The cool and humid climate, with ∼800 mm annual average rainfall concentrated in the summer, is very conducive for establishing the artificial and natural epiphytotic condition of STB infection.

**Core Ideas**

- Resistant germplasm identification to Septoria tritici blotch of wheat was investigated.
- The authors identified molecular markers/genomic regions associated with Septoria tritici blotch resistance.
- A genome wide association study was used to analyze Septoria tritici blotch resistance in South Asian spring wheat panel.

The genotypes were sown in plots of double rows of 0.75 m spaced 25 cm apart in two replications in a randomized complete block design. For the creation of STB epiphytotic condition, a mixture of six aggressive isolates, namely, St1 (B1), St2 (P8), St5 (OT), St6 (KK), 64 (St 81.1), and 86 (St 133.4), having a concentration of 1 × 10^7^ spores/mL of Z. tritici, was prepared as per the procedure of Gilchrist et al. (2006). The initial inoculation was conducted with an ultra‐low volume applicator at the Zadoks growth stage (ZGS) 29 (ZADOKS et al., 1974), followed by two additional applications at weekly intervals. The data collection for STB infection in the field began at ZGS 60 and was performed thrice at weekly intervals. The double‐digit scale (00–99) for rating foliar diseases was used to evaluate STB severity (Saari & Prescott, 1975). According to this, the first digit of the scale denotes the relative height of the vertical disease spread, and the second number denotes the severity of the condition based on the amount of leaf area affected by the condition. The STB severity percentages were calculated with the following formula: STB severity (%) = (first digit/9) × (second digit/9) × 100, which was subsequently used to calculate the area under the disease progression curve (AUDPC). PH and DH were recorded over the years and replications, where PH was measured in centimeters, including the height from the ground to the spike tips, excluding awns, and DH was scored for each plot in the number of days from sowing to the day when approximately 50% of the spikes emerged.

### 2.2 Statistical analysis of the phenotypic data

The outliers of the data for all traits under study were identified with the *Z*‐score test, and genotypes with a *Z*‐score <− 3.0 or >3.0 were removed. The boxplots for STB, PH, and DH were generated over the years to describe the mean values and spread of variation with the ggplot2 package (Hadleyy, 2016) in R‐software version 4.0.1 (R Core Team, 2021). The simple and combined analysis of variance (ANOVA) was performed over the years for STB, PH, and DH, considering the genotypes and years as random effects. The analysis was performed with R‐based STAR software version 2.0.1 (STAR, 2014). The best linear unbiased predictions (BLUPs) with adjusted means were calculated considering the replication, genotypes, and years as random effects separately for each year and pool over the years using META‐R version 6.0 (Alvarado et al., 2020).

To draw information on the interrelationship among the STB, DH, and PH, Pearson's correlation coefficient analysis was carried out with the Psych package (William, 2023) in R‐software version 4.0.1 (R Core Team, 2021).

#### 2.2.1 Genotyping, population structure, and linkage disequilibrium analysis

Genomic DNA was extracted with the cetyltrimethylammonium bromide method (Doyle & Doyle, 1987) and the quantity and quality of DNA considering the UV absorbance ratio of A260/A280 and A260/A230 was estimated with a NanoDrop spectrophotometer. The GWAS panel was genotyped with Illumina Infinium 15 K Bead Chip of Trait Genetics GmbH. The GWAS analysis was performed on the filtered genotypic dataset of 10,775 highly informative SNP markers, with those having >30% missing value and minor allele frequency <5.0% removed. Population structure was determined using STRUCTURE v 2.3.4 (Pritchard et al., 2000) by keeping the 1,00,000 length of burn‐in period and 1,00,000 Markov Chain Monte Carlo reps with three iterations. The optimum population number (*K*) value was determined by the ad hoc ∆*K* method (Evanno et al., 2005) by keeping the range of *K* from 1 to 10. The ∆*K* approach was used to access the actual subpopulations (Earl & Vonholdt, 2012), which was confirmed by the Evanno method (Evanno et al., 2005) using the STRUCTURE HARVERSTER program (Earl & Vonholdt, 2012). The constellation plot and hierarchical cluster were generated with the Ward method in JMP v.16 (Lehman et al., 2013) to reconfirm the population structure. The principal component analysis (PCA) over the genotypic data was conducted by plotting the PC1 over PC2 using the R software (R Core Team, 2021). The linkage disequilibrium (LD) decay at the half‐life (*R* = 0.5) analysis was performed separately for each sub‐genome (A, B, and D) and across the genome. The LD parameters (*R* ^2^ value) among the SNP markers were calculated using the LD function by keeping the window size of 50 in trait analysis by association, evolution, and linkage (Bradbury et al., 2007). The LD plot was drawn by placing the *R* ^2^ value against the physical distance with the critical threshold line by taking the average of the *R*‐value of unlinked markers with R software version 4.0.1 (R Core Team, 2021).

### 2.3 Marker‐trait association analysis

The marker‐trait association (MTA) was performed with filtered genotypic data and the generated BLUP values separately for each year and pooled over the years. The analysis was performed with three association models, namely, mixed linear model (MLM), Bayesian‐information and linkage‐disequilibrium iteratively nested keyway (BLINK), and fixed and random model circulating probability unification (FarmCPU) algorithms in the GAPIT version 3.0 (J. Wang & Zhang, 2021). The −log *p* value = 3.5 was used as the threshold for significant MTAs for FarmCPU and BLINK. However, the −log *p* value = 3.0 was used for MLM to avoid false negative results due to overfitting the model. The adjusted p*‐value* threshold of significance was also calculated for multiple comparisons to avoid the false positive with a Bonferroni correction with a cut‐off ≤0.05 (Haynes, 2013).

The selection of resistant genotypes was accomplished by calculating the genomic estimated breeding value (GEBV) based on the main additive effect of loci with genomic best linear unbiased prediction (gBLUP) analysis (VanRaden, 2008). The GEBV values were calculated for STB, DH, and PH using the phenotypic BLUP values for each year and pooled over the years.

In gBLUP analysis, phenotypic data (*y*) are regressed over the marker genotypes in a linear model of the form *y *= 1*μ* + **X** **β** + **e**, where *μ* is a common intercept, **X** is an *n* × *p* incidence matrix of marker genotypes, **β** is a *p* × 1 vector of marker effects, and **e** is an *n* × 1 vector of error terms with the assumption that $\beta ∼N(0,I\sigma_{\beta }^{2})$ and $e∼N(0,I\sigma_{e}^{2})$ (Muqaddasi et al., 2019; VanRaden, 2008).

A two‐tailed *t*‐test of significance at a 95% confidence interval was conducted to understand the effect of favorable alleles and the non‐favorable alleles of identified QTLs based on the mean difference of AUDPC values and visualized with the “*ggpubr*” package (Alboukadel, 2023). The STB mean value differentiating QTLs was utilized to understand the frequency of favorable alleles in the genotypes. We compared the mean AUDPC value with the frequency of favorable alleles with regression and boxplot analysis of the mean value of STB, which was visualized with ggplot2 (Hadleyy, 2016).

### 2.4 Candidate gene identification and in silico expression analysis

The identified QTNs associated with STB were utilized in an in silico search of the putative candidate genes (CGs) against the IWGSC‐RefSeq version 1.1 in the Ensembl Wheat database (EnsemblPlants, n.d.) (http://plants.ensembl.org/index.html), and gene functions were annotated against the same database in the TGT database (Triticeae Gene Tribe, n.d.) (http://wheat.cau.edu.cn/TGT/m4/?navbar = GO Enrichment) considering the multi‐test adjustment with false discovery rate at *α* = 0.05. The block size adjacent to target SNPs for searching against the Ensembl Wheat database was decided based on the size of the block on LD decay at the half‐life.

## 3 RESULTS

### 3.1 Significant phenotypic variation and high heritability of Septoria tritici blotch, plant height, and days to heading

**FIGURE 1:**
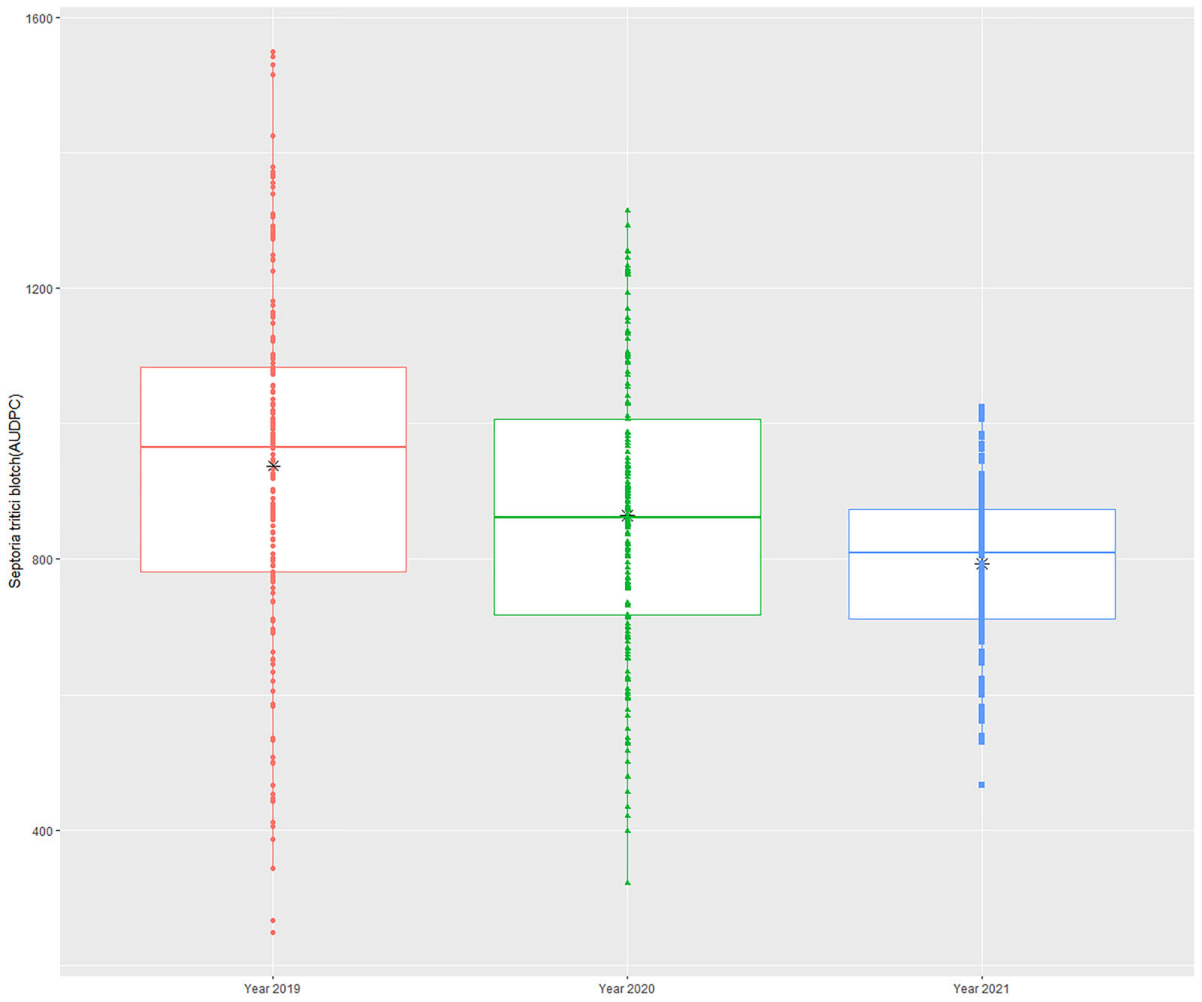
AUDPC boxplot Septoria tritici blotch across the years.

**FIGURE 2:**
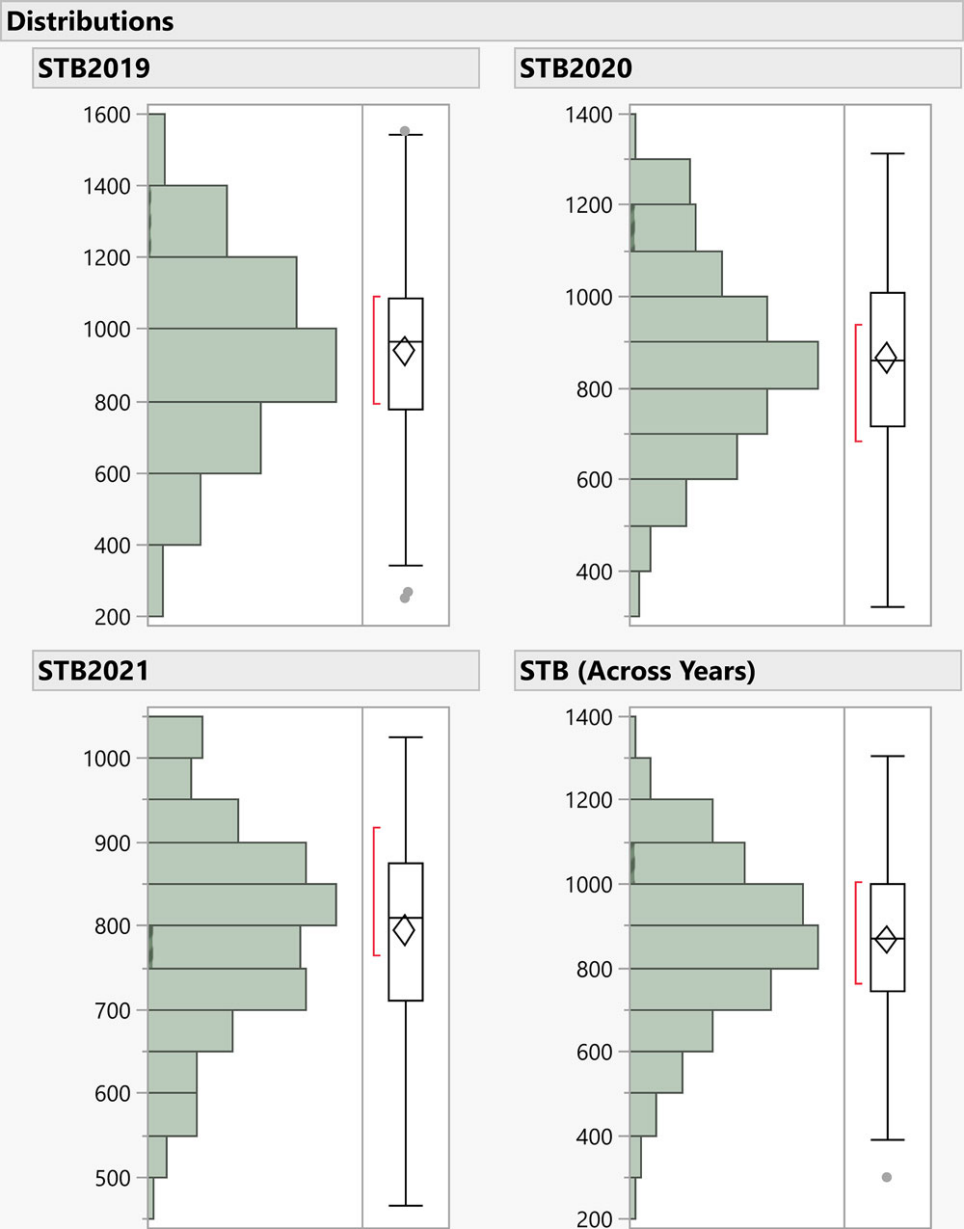
Normal distribution of Septoria tritici blotch (STB) infection across the years. STB2019, Septoria tritici blotch infection in year 2019; STB2020, Septoria tritici blotch infection in year 2020; STB2021, Septoria tritici blotch infection in year 2021.

The STB, DH, and PH boxplots over the years have been demonstrated in Figure 1 and Figure S1, with mean values as black “⁎.” The mean value and spread of variability between the first and third quartile for STB (Figure 1) were recorded as lowest in 2021, followed by the year 2020, indicating a less conducive year for STB spread despite the similar creation of epiphytotic conditions. Over the years, the BLUP values of STB infection had a normal distribution indicating the presence of minor effect genes in controlling STB resistance (Figure 2). The simple ANOVA indicated the presence of significant genotypic differences for STB, DH, and PH in each respective year (Table 1; Table S2). The heritability (*h* ^2^) of STB infection was recorded as 0.84 for the year 2019, 0.89 for the year 2020, and 0.62 for the year 2021 (Table 1), while the range of *h* ^2^ was recorded as 0.91–0.97 for DH and 0.92–0.97 for PH over the years (Table S2). The combined ANOVA revealed the significant differences for genotypic, year, and genotypic × year effects for STB, DH, and PH, and the *h* ^2^ was recorded as 0.87 for STB, 0.86 for DH, and 0.73 for PH (Table 1; Table S2).

**TABLE 1:** Statistical and genetic parameters for Septoria tritici blotch (STB) over the respective year and pool over years.

Statistical parameters	STB 2019	STB 2020	STB 2021	STB_POOL
Heritability	0.84	0.89	0.62	0.87
Genotype variance	81934.9	48571.8	22479.9	44398.7
Genotype × year variance	–	–	–	6596.8
Year variance	–	–	–	4932.5
Residual variance	31180.62	11949.09	27564.83	23564.8
Grand mean	937.11	864.61	793.58	865.1
LSD	319.47	203.61	258.03	207.7
CV	18.84	12.64	20.92	17.7
Genotype significance	0	0	4.78E‐10	7.05E‐61
Genotype × year significance	–	–	–	4.97E‐06
Year significance	–	–	–	0.017097

Abbreviations: STB_POOL, Septoria tritici blotch infection across years.

### 3.2 Negative relationship of STB with plant height and days to heading

Pearson's pairwise correlation coefficient based on the BLUP values indicated that DH and PH significantly negatively correlated with STB each year and pooled over the years (Figure S2). The *STB across years* had a higher negative correlation with *DH across years* (*r* = −0.46; *p* < 0.001) than with *PH across years* (*r* = −0.31; *p* < 0.001), while *DH across years *and* PH across years* had a weak positive correlation (*r* = 0.25; *p* < 0.001). The correlation of STB with DH and PH gives the idea to take care of the pleiotropic effect of the genes in interpreting the result and deployment of genes. The correlations among the different years for STB were significant, ranging from 0.72 to 0.79 (Figure S2).

### 3.3 Population structure and linkage disequilibrium analysis

**FIGURE 3:**
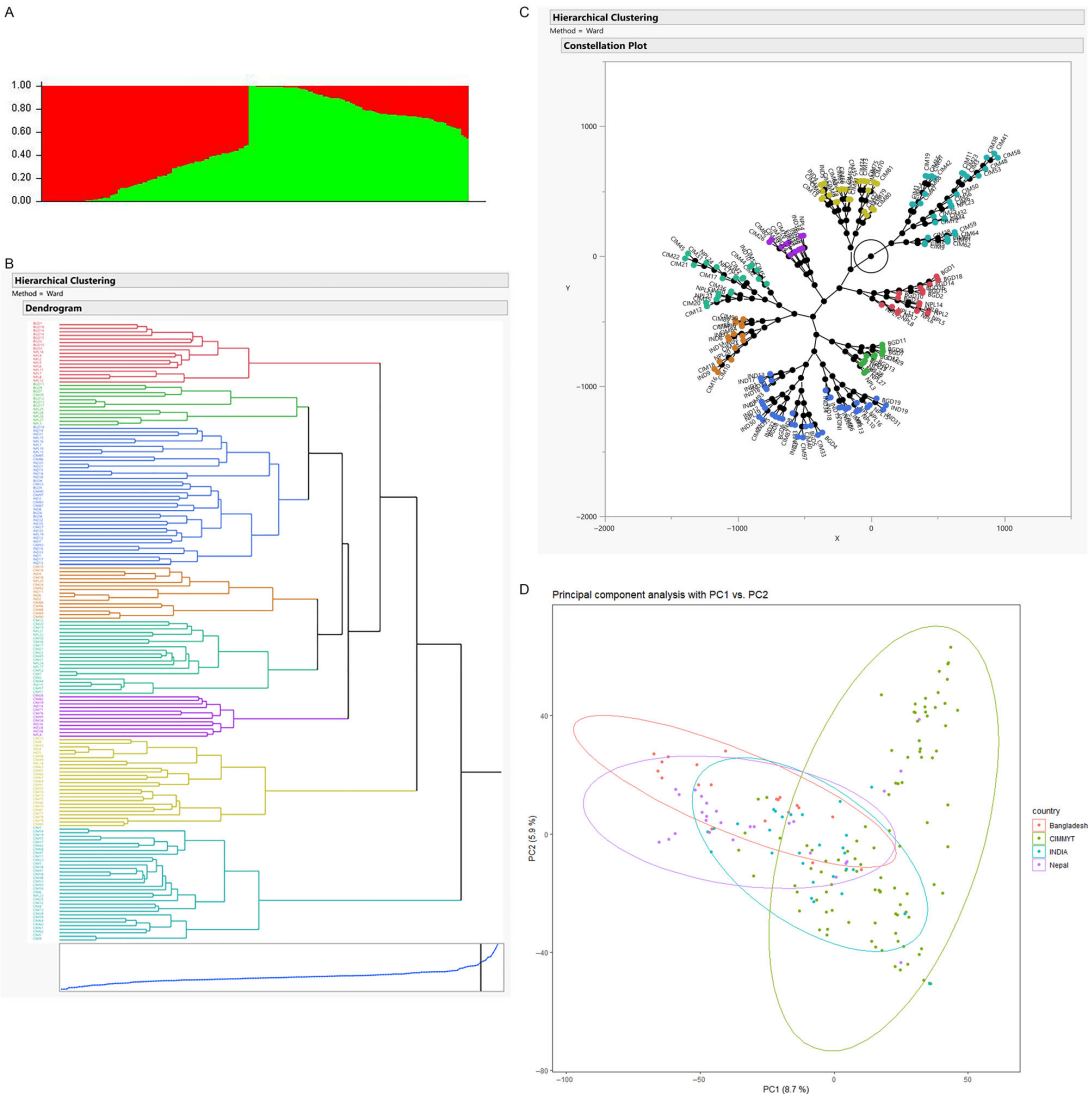
SNPs‐based population structure. (A) Clusters were plotted against ΔΚ. (B) Hierarchical cluster. (C) Constellation plot. (D) PCA plot.

**FIGURE 4:**
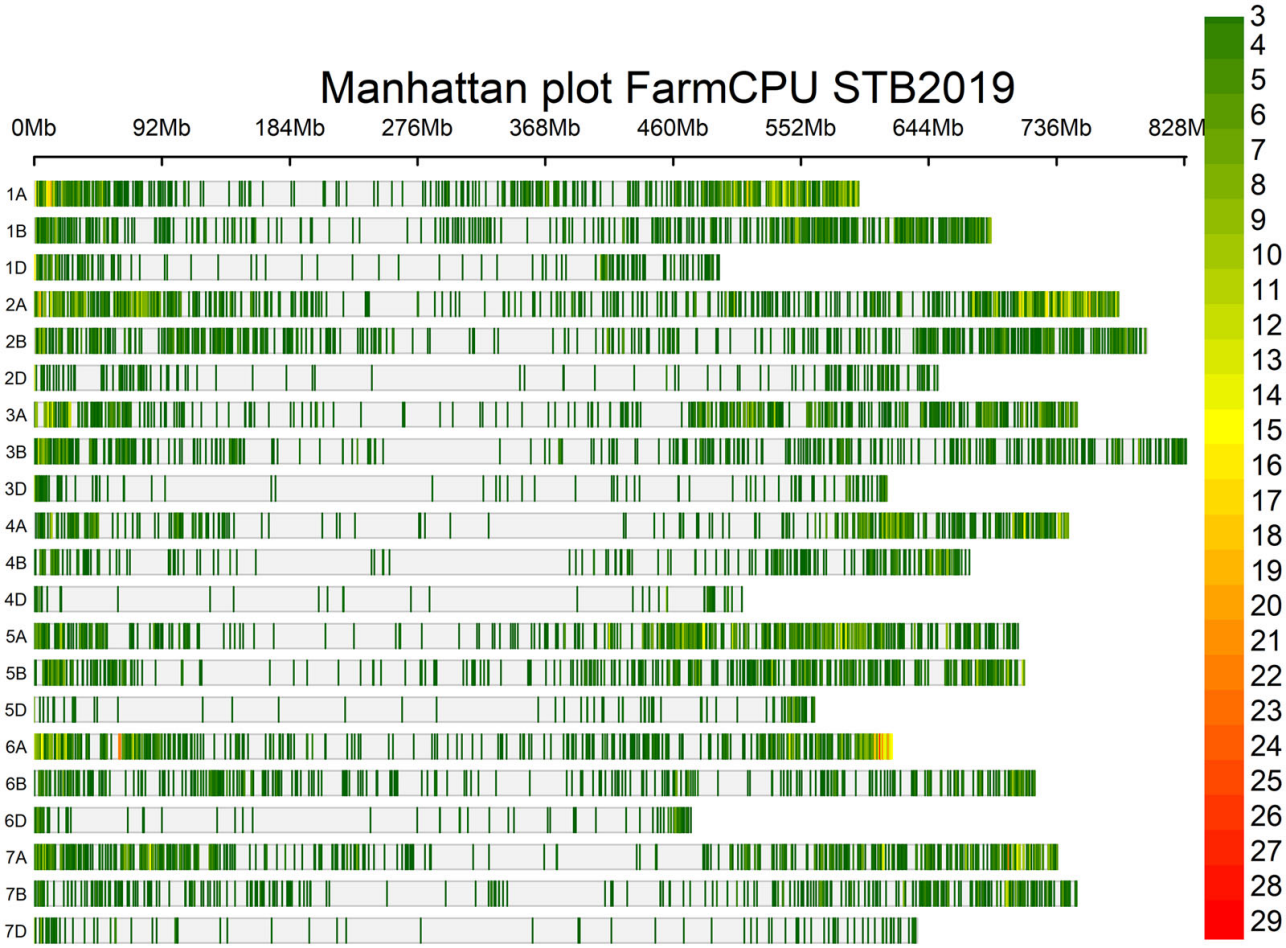
Single nucleotide polymorphism (SNPs) density across the genome. FarmCPU, fixed and random model circulating probability unification; STB2019, Septoria tritici blotch infection in year. 2019.

**FIGURE 5:**
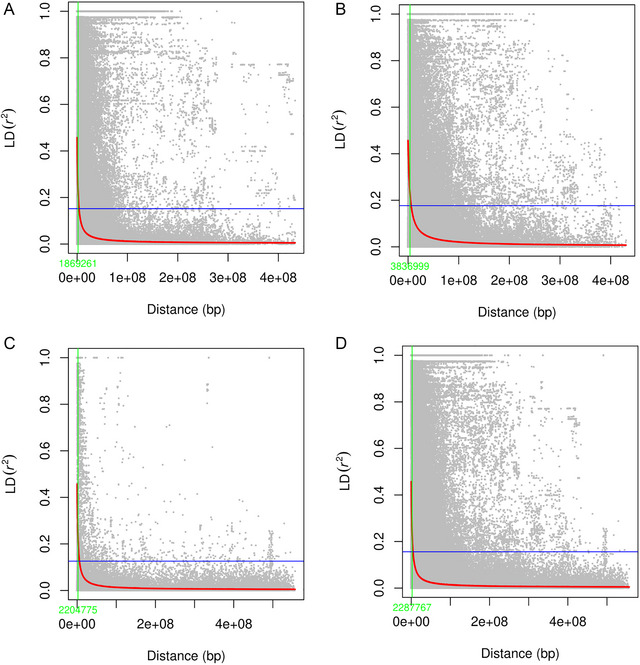
(A) Linkage disequilibrium (LD) decay_Genome A. (B) LD decay_Genome B. (C) LD decay_Genome D LD decay_Whole genome.

The GWAS panel was subdivided into two sub‐populations when the clusters were plotted against Δ*Κ* (Figure 3a), further confirmed by the hierarchical cluster and constellation plot (Figure 3b,c). The PCA based on genotypic data revealed that some lines of CIMMYT origin had fallen outside of the group even though PC1 and PC2 were controlling the small percentage of variation (Figure 3d). The filtered set of 10,775 high‐quality SNP markers was distributed across the genome in which A genome (6247) having the highest number of markers followed by the B genome (3660) and the D genome (868) (Figure 4). The LD‐decay of the whole genome to its half‐life was estimated to be 2.28 Mb, while B genome was having the highest LD‐decay length (3.83 Mb), followed by D genome (2.20 Mb) and A genome (1.86 Mb) (Figure 5A–D). LD decay plots of specific chromosomes were also constructed with the same principle (Figure S3A–F). The positioning of previously reported STB genes and MQTLs with QTLs identified in this study was accomplished based on the LD block size of the specific chromosome arm.

### 3.4 Marker‐trait associations for Septoria tritici blotch, plant height, and days to heading

A total of 91 QTNs for STB, 57 QTNs for PH, and 63 QTNs for DH were detected based on GWAS models being utilized, in which some of the QTNs for STB were also detected for PH and DH (Table 2; Tables S3 and S4). The different GWAS algorithm models have generated different QTNs for traits under study. The 40 QTNs were identified with BLINK, 45 with FarmCPU, and 44 with MLM model for STB over the respective year and pooled over the years. Some QTNs were common across the models and years, showing the high reliability of the marker for plymerase chain reaction (PCR)‐based SNPs markers like KASP (kompetitive allele specific primer) assay and their effective use in marker‐assisted selection. In this paper, the QTLs associated with STB are only focused on. However, specific QTNs associated with PH and DH having the LD and common with QTNs of STB are mentioned.

**TABLE 2:** The repetitive quantitative trait nucleotides (QTNs) controlling the Septoria tritici blotch resistance.

								Mean differentiating ability (*p*‐value)			
QTNs	SNP	Chromosome	Position	Effect	−log10 *p*‐value	Phenotypic variation (%)	Year/model	STB2019	STB2020	STB2021	STB_POOL
Q.CIM.stb.1AL.3	AX‐94584593	1A	5.28E+08	55.8–2240	6.65–4.14	7.96	BLINK_2020; FarmCPU_2020	3.2 E‐8	2.00E‐09	0.0002	3.40E‐08
Q.CIM.stb.1AL.4	wsnp_CAP8_rep_c3938_1936666	1A	5.47E+08	45–1664	5.52–5.8	2.29	BLINK_ POOL YEARS; FarmCPU_POOL YEARS	4.10E‐08	1.50E‐08	8.60E‐07	3.00E‐08
Q.CIM.stb.1AL.5	AX‐94967337	1A	5.63E+08	41.8–928	5.59–6.02	4.01–5.11	BLINK_2020; FarmCPU_2020	1.00E‐06	1.10E‐09	1.30E‐06	1.10E‐07
Q.CIM.stb.1BL.4	RAC875_c27939_335	1B	6.36E+08	−51.3 to −43.4	5.75–9.4	2.07	FarmCPU_POOL YEARS; FarmCPU_2020	0.097	0.024	0.031	0.024
Q.CIM.stb.2AL.5	GENE‐4029_80	2A	6.24E+08	66.8–448	3.6–9.89	1.2‐6.28	BLINK_2019; BLINK_ POOL YEARS; FarmCPU_POOL YEARS	7.20E‐11	8.50E‐09	1.70E‐08	3.20E‐09
Q.CIM.stb.2BL.3	RAC875_c10132_462	2B	7E+08	73.2–99.6	3.02–3.14	–	MLM_POOL YEARS; MLM_2019	0.00028	0.0017	0.00019	0.00031
Q.CIM.stb.2BL.6	Ra_c13298_783	2B	7.32E+08	30–114	3.83–6.32	1.74	FarmCPU_2021; MLM_POOL YEARS; MLM_2019; MLM_2021	0.1	0.28	0.35	0.1
Q.CIM.stb.2DL.2	AX‐94670144	2D	3.5E+08	33.7–640	3.16–10.34	4.3–9.11	BLINK_2020; BLINK_2019; BLINK_ POOL YEARS; FarmCPU_POOL YEARS; FarmCPU_2019; FarmCPU_2020; MLM_2019	2.20E‐12	9.20E‐09	2.70E‐08	2.80E‐10
Q.CIM.stb.3AL.2	BS00048031_51	3A	5.35E+08	−100.3 to −91.2	3.01–3.08	–	MLM_POOL YEARS; MLM_2020	0.042	0.013	0.0033	0.01
Q.CIM.stb.3AL.3	BobWhite_c18408_199	3A	5.35E+08	−111.1 to −94.9	3.09–3.55	–	MLM_POOL YEARS; MLM_2020	0.043	0.014	0.0032	0.011
Q.CIM.stb.3AL.4	Excalibur_c52446_519	3A	5.38E+08	51.3–123.4	3.68–4.36	–	FarmCPU_2020; MLM_POOL YEARS; MLM_2020	0.043	0.013	0.0031	0.01
Q.CIM.stb.3AL.5	AX‐94495697	3A	5.56E+08	−76.5 to −77.5	3.12–3.55	–	MLM_POOL YEARS; MLM_2020				
Q.CIM.stb.3DL.1	Ra_c9608_183	3D	5.03E+08	83.3–608	3.27–3.69	–	BLINK_2019; MLM_2019				
Q.CIM.stb.4BL.6	BobWhite_c4256_213	4B	6.57E+08	−36.7 to 1120	5.53–3.71	1.53	BLINK_2019; FarmCPU POOL YEARS	0.0017	0.01	0.00021	0.0013
Q.CIM.stb.5AL.13	Excalibur_c26671_57	5A	5.91E+08	−38.5 to 592	3.53–6.27	5.62	BLINK_2020; BLINK_ POOL YEARS; FarmCPU_POOL YEARS	1.10E‐09	3.10E‐11	1.20E‐07	1.70E‐10
Q.CIM.stb.5AL.5	AX‐94406443	5A	5.71E+08	−105.3 to 528	3.18–6.58	4.07–6.14	BLINK_2020; FarmCPU_2019; MLM_2019; MLM_2020	5.1 E‐10	1.30E‐07	0.00043	4.80E‐07
Q.CIM.stb.6AS.1	AX‐94653030	6A	2178375	45.4–448	6.05–6.23	6.21–16.65	BLINK_2021; FarmCPU_2021	0.0069	0.00013	7.60E‐06	0.00042
Q.CIM.stb.6AS.2	Tdurum_contig62941_85	6A	23410186	−121.2 to −88.5	3.71–6.38	5.32	FarmCPU_2019; MLM_POOL YEARS; MLM_2019	5.50E‐05	1.20E‐05	0.0013	1.70E‐05
Q.CIM.stb.7AL.3	Excalibur_c95707_285	7A	6.72E+08	59.8–528	3.03–8.42	3.84	BLINK_2019; MLM_POOL YEARS; MLM_2019	2.20E‐07	4.30E‐05	0.004	8.20E‐06
Q.CIM.stb.7BL.1	BS00101087_51	7B	6.78E+08	−26.6 to 1728	4.13–4.27	–	BLINK_2021; FarmCPU_2021	0.00023	1.50E‐06	9.40E‐09	2.20E‐06
Q.CIM.stb_dh.2BL.9	AX‐95084761	2B	7.85E+08	−90.1 to −60.8	3.01–3.48	–	MLM_POOL YEARS; MLM_2019	0.12	0.13	0.055	0.056
Q.CIM.stb_dh.2DL.3	AX‐94787485	2D	5.91E+08	73.4–98.6	3.01–3.05	–	MLM_POOL YEARS; MLM_2019	0.0003	0.0089	0.0014	0.0006
Q.CIM.stb_dh.3AL.6	RAC875_c61934_186	3A	7.34E+08	−57.1 to −50.4	5.53–6.07	3.39–5.91	FarmCPU_2019; FarmCPU_2020	1.40E‐05	0.0053	0.0092	0.00076

Abbreviations: BLINK, Bayesian‐information and linkage‐disequilibrium iteratively nested keyway; CIm, CImmYT; FarmCPU, fixed and random model circulating probability unification; MLM, mixed linear model; SNp, single nucleotide polymorphism; STB2019, Septoria tritici blotch infection in year 2019; STB2020, Septoria tritici blotch infection in year 2020; STB2021, Septoria tritici blotch infection in year 2021; STB_POOL, Septoria tritici blotch infection across years.

### 3.5 Twenty‐three repetitive and 68 unique marker‐trait associations for STB

The 23 associated QTNs with STB were common across the different models and years (Table 2), while 68 QTNs were unique for respective models and years (Table S3). The QTN, Q.CIM.stb.2DL.2, located on chromosome 2D with associated SNPs AX‐94670144, was identified across the years and in different GWAS models with the −log10 *p*‐value ranging from 3.16 to 10.34. The QTN, Q.CIM.stb.2AL.5, located on 5A with associated SNPs, GENE‐4029_80 was identified in 2019 and pooled over the years under BLINK and FarmCPU models with the −log10 *p*‐value ranging from 3.60 to 9.88 (Figure 6A,B). Likewise, the two QTNs, namely, Q.CIM.stb.2BL.3 and Q.CIM.stb.2BL.6, located on 2B with associated SNPs RAC875_c10132_462 and Ra_c13298_783, were picked up in 2019 and pooled over the years under MLM and FarmCPU models with the −log10 *p*‐value ranging from 3.01 to 6.32 (Figure 6A; Figure S5). The QTN, Q.CIM.stb.5AL.5, located on 5A with associated SNPs AX‐94406443, was identified in 2019 and 2020, respectively, with all three models with the −log10 *p*‐value ranging from 3.17 to 6.57. Similarly, the QTN, Q.CIM.stb.5AL.13, located on 5A with associated SNPs, Excalibur_c26671_57, was identified in 2020 and pooled over the years with BLINK and FarmCPU models with the −log10 *p*‐value ranging from 3.53 to 6.27. The two QTNs, namely, Q.CIM.stb.6AS.2 and Q.CIM.stb.7AL.3, located on 6A and 7A with associated SNPs Tdurum_contig62941_85 and Excalibur_c95707_285 were identified in the year 2019 and pooled over the years with repeated models with the −log10 *p*‐value ranging from 3.71 to 6.37 for Q.CIM.stb.6AS.2 and 3.03 to 8.42 for Q.CIM.stb.7AL.3, respectively (Table 2; Figure 6A,B; Figure S5). Other repetitive QTNs across the different years and algorithm models have been presented in Table 2.

FIGURE 6(A) Manhattan Plots FarmCPU_Septoria tritici blotch. (B) Manhattan Plots BLINK_Septoria tritici blotch. BLINK, Bayesian‐information and linkage‐disequilibrium iteratively nested keyway; FarmCPU, fixed and random model circulating probability unification. STB2019, Septoria tritici blotch infection in year 2019; STB2020, Septoria tritici blotch infection in year 2020; STB2021, Septoria tritici blotch infection in year 2021.

**Figure:**
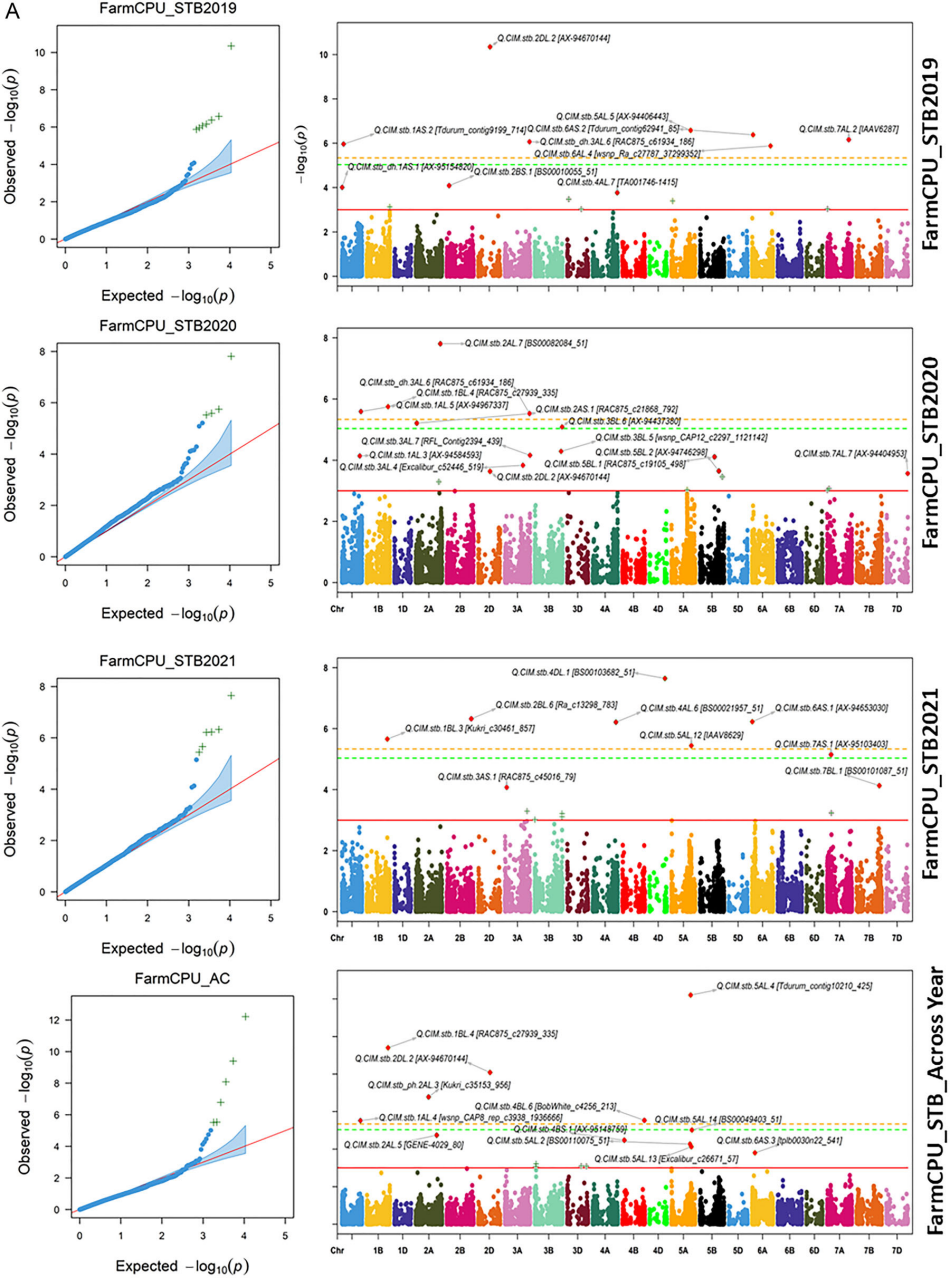

**Figure:**
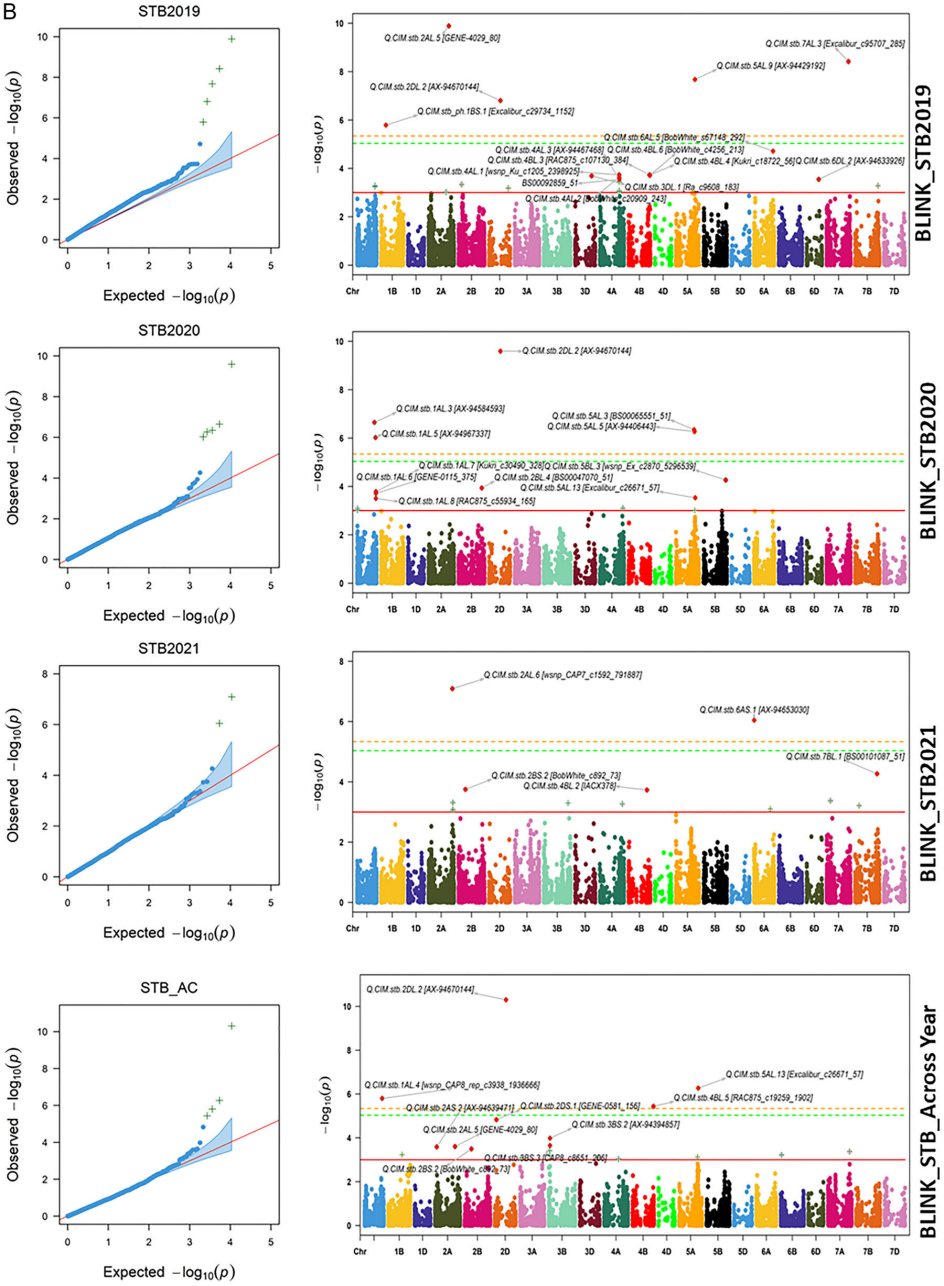

Some of the unique QTNs over the respective year with different algorithm models were associated with STB, which is relevant in the presence of significant effects of year and genotype × year interaction. In 2019, QTNs, namely, Q.CIM.stb.1AS.2, Q.CIM.stb.6AL.4, and Q.CIM.stb.7AL.2 with FarmCPU model, and Q.CIM.stb_ph.1BS.1 and Q.CIM.stb.5AL.9 with BLINK model, were identified surpassing the strict threshold of Bonferroni correction (−log10 *p*‐value 5.33 at *p* < 0.05). Likewise, QTNs, namely, Q.CIM.stb.2AL.7 with FarmCPU and Q.CIM.stb.5AL.3 with BLINK, were identified for 2020. The QTNs, namely, Q.CIM.stb.1BL.3, Q.CIM.stb.4AL.6, Q.CIM.stb.4DL.1, and Q.CIM.stb.5AL.12 with FarmCPU; and Q.CIM.stb.2AL.6 were identified with BLINK model for year 2021. Meanwhile, the QTNs, Q.CIM.stb.4BL.5 surpassing the Bonferroni threshold level for pool over the years was identified (Table S3; Figure 6A,B; Figure S5).

### 3.6 Common QTNs for Septoria tritici blotch, plant height, and days to heading

The DH and PH being negatively correlated with AUDPC values of STB infection indicate some common genomic regions responsible for all these traits. Some QTNs governing the STB were also associated with DH and PH. The QTNs, namely, Q.CIM.stb_ph.7AL.4, Q.CIM.stb_ph.2AL.3, and Q.CIM.stb_ph.1BS.1, associated with SNPs wsnp_JD_c20555_18262260, Kukri_c35153_956, and Excalibur_c29734_1152, respectively, were common for STB and PH (Table S3; Figure S4), whereas the QTNs, namely, Q.CIM.stb_dh.2BL.9, Q.CIM.stb_dh.2DL.3, and Q.CIM.stb_dh.2AL.6, were repeatable QTNs (Table 2), and Q.CIM.stb_dh.7AL.5 and Q.CIM.stb_dh.1AS.1 were the unique QTNs governing the STB and DH (Table S3). However, the common QTNs responsible for DH and PH were not observed in the present study.

### 3.7 Linkage disequilibrium among the QTNs of Septoria tritici blotch, plant height, days to heading, and STB genes

**FIGURE 7:**
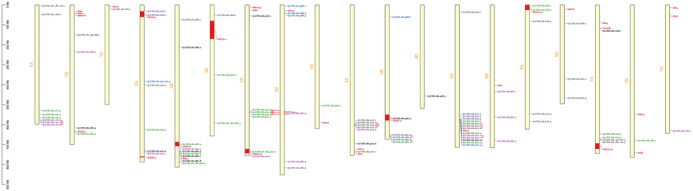
Newly identified quantitative trait nucleotides (QTNs) along with previously reported quantitative trait loci (QTLs) and Septoria tritici blotch (STB) genes across the genome.

Many identified QTNs about STB, PH, and DH were in the LD with each other with *R* ^2^ > 0.80. Some of the QTNs of STB resistance were in the haplotype block with themselves rather than the QTNs of PH and DH. The QTNs pertaining to STB and the reported STB genes across the genome based on physical positions have been depicted in Figure 7. The QTNs, namely, Q.CIM.stb.1AL.6, Q.CIM.stb.1AL.7, and Q.CIM.stb.1AL.8 located on 1A; Q.CIM.stb.3AL.2, Q.CIM.stb.3AL.3, and Q.CIM.stb.3AL.4 located on 3A; Q.CIM.stb.4AL.2, Q.CIM.stb.4AL.3, and Q.CIM.stb.4AL. located on 4A; and Q.CIM.stb.5AL.6, Q.CIM.stb.5AL.7, Q.CIM.stb.5AL.8, Q.CIM.stb.5AL.10, and Q.CIM.stb.5AL.11 located on 5A were in the haplotype blocks (Table S5). Interestingly, a QTL, that is, Q.CIM.stb.3BL.4 located on 3B, was in complete LD (LD = 1.0) with three QTLs, namely, Q.CIM.stb.3AL.2, Q.CIM.stb.3AL.3, and Q.CIM.stb.3AL.4 located on 3A, while Q.CIM.stb_ph.2AL.3 associated with STB and PH had the complete LD with Q.CIM.stb.4AL.4 located on 4A (Table S5). Some of the QTLs associated with DH and PH were also in haplotype blocks and LD with each other.

#### 3.7.1 The effects of 2NS/2AS translocation on STB resistance

**FIGURE 8:**
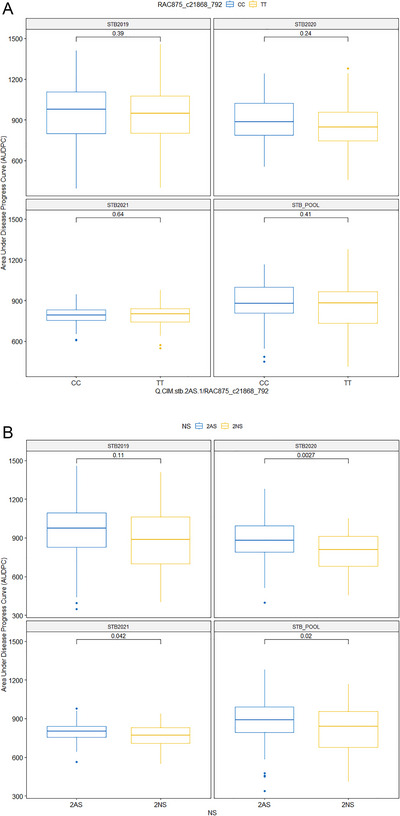
(A) Mean differentiating ability of Q.CIM.stb.2AS.1. (B) Mean differentiating ability of 2AS/NS translocation. STB2019, Septoria tritici blotch infection in year 2019; STB2020, Septoria tritici blotch infection in year 2020; STB2021, Septoria tritici blotch infection in year 2021; STB_POOL, Septoria tritici blotch infection across years.

Two QTNs, namely, Q.CIM.stb.2AS.1 at 32 Mb and Q.CIM.stb.2AS.2 at 52 Mb, were identified close to 2NS/2AS translocation regions (0–24 Mb) on the short arm of 2A. In our previous finding, we reported that multiple SNPs forming an LD block size of 0–35.4 Mb were associated with wheat blast resistance (He et al., 2021). The Q.CIM.stb.2AS.1 located within the LD block governing the blast resistance (He et al., 2021) could not differentiate the GWAS panel into resistant versus susceptible groups. However, 2AS/2NS translocations segment, which was verified with STS markers, namely, Ventriup, cslVrgal3, WGGB156, and WGGB159 (He et al., 2021), differentiated the resistant and susceptible groups in STB infection in year 2020, STB infection in year 2021, and pool over the years (Figure 8A,B).

### 3.8 Additive effect of favorable alleles in gaining STB resistance and resistant germplasm

**FIGURE 9:**
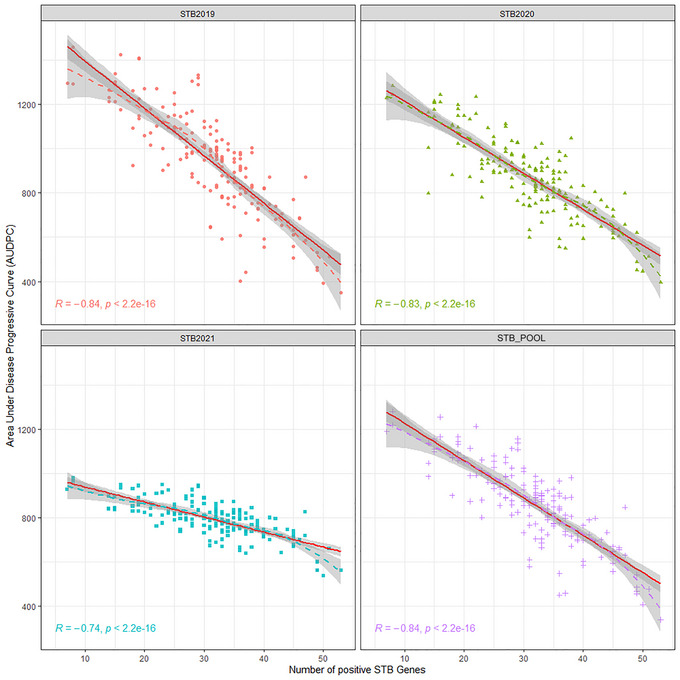
Negative relationship of Septoria tritici blotch (STB) infection with increasing the number of favorable alleles. STB2019, Septoria tritici blotch infection in year 2019; STB2020, Septoria tritici blotch infection in year 2020; STB2021, Septoria tritici blotch infection in year 2021; STB_POOL, Septoria tritici blotch infection across years.

**FIGURE 10:**
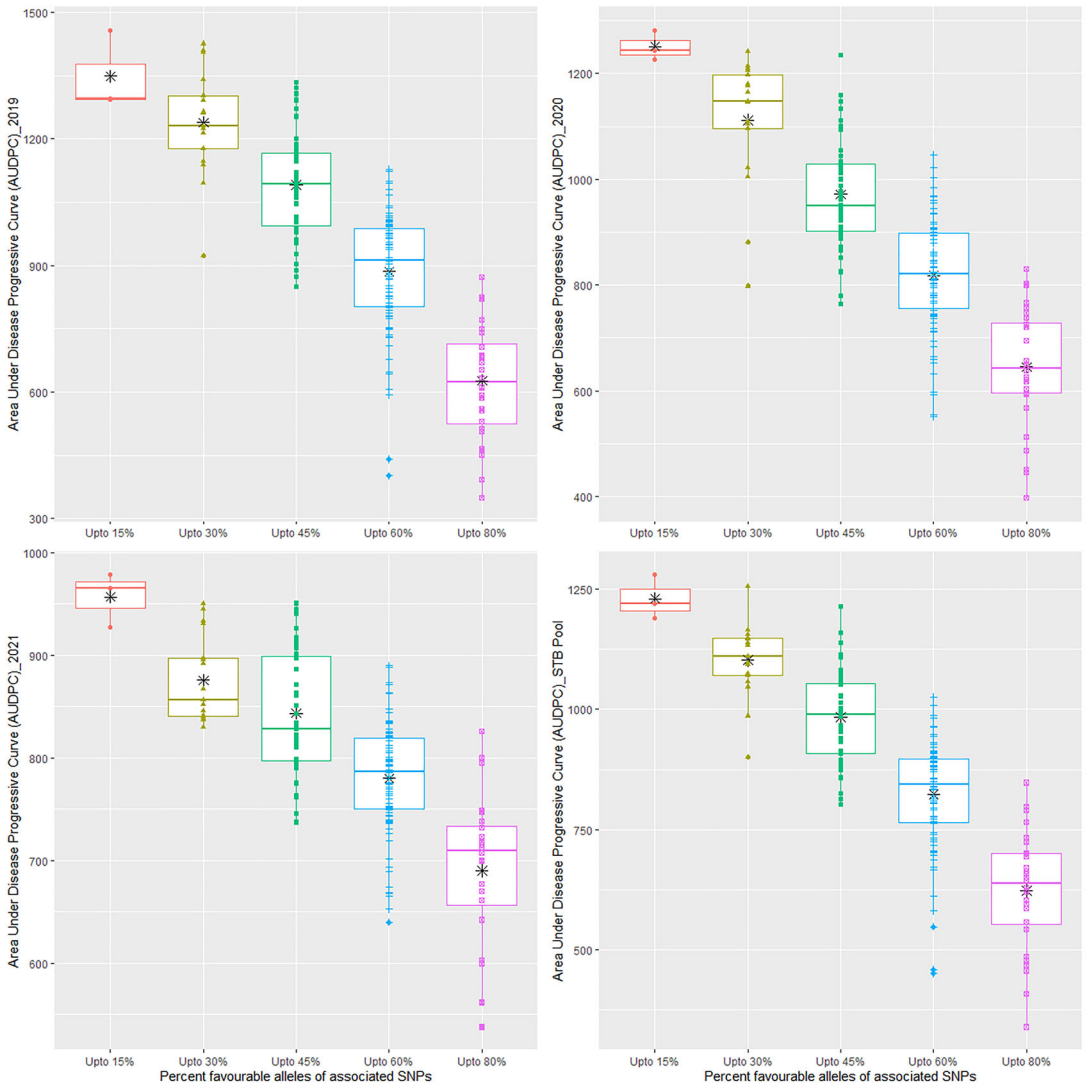
Decreasing trend of Septoria tritici blotch (STB) infection with stacking of favorable alleles of quantitative trait nucleotides (QTNs). SNP, single nucleotide polymorphism.

**FIGURE 11:**
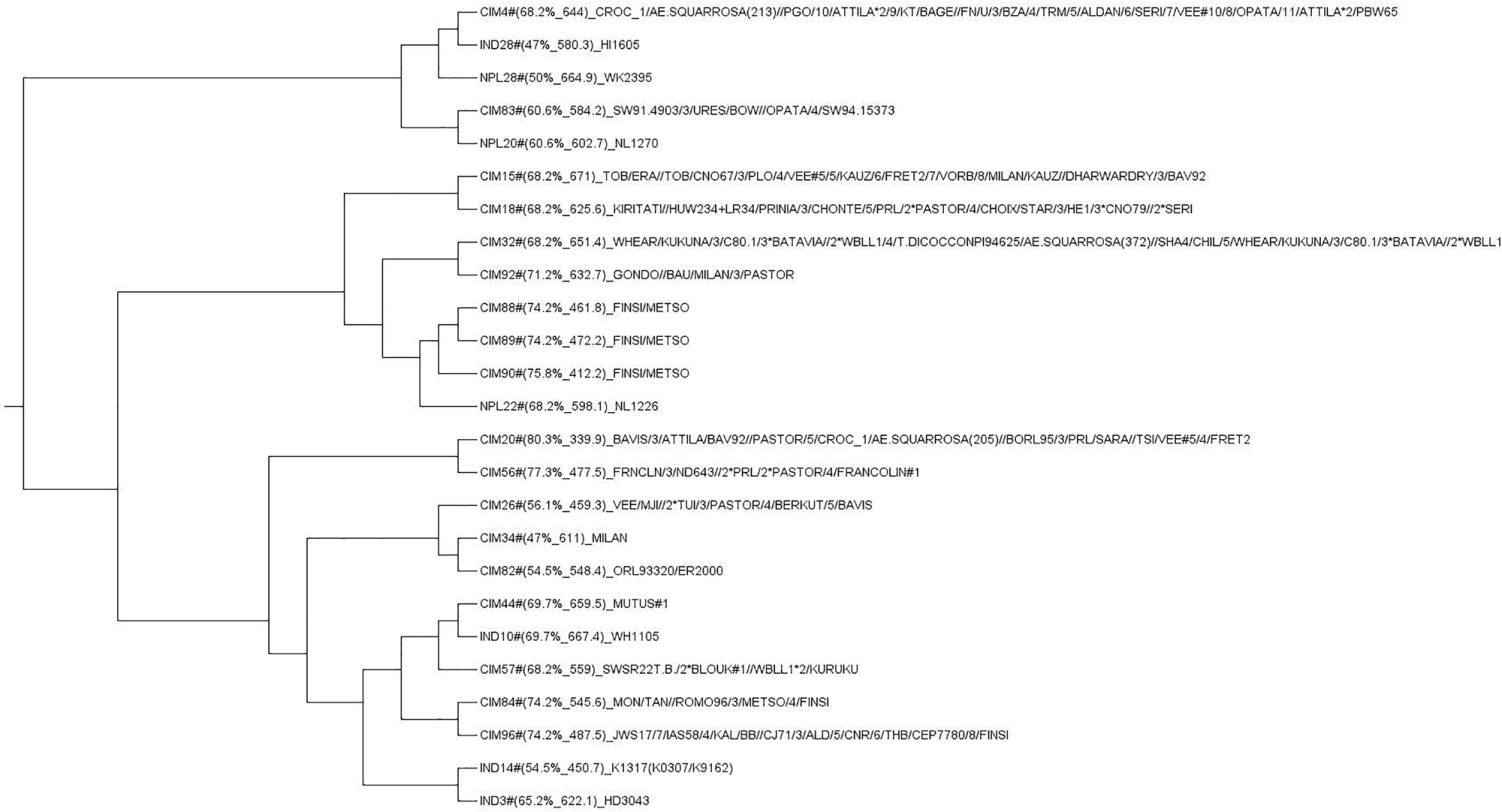
Neighbor‐joining‐based hierarchical cluster analysis with quantitative trait nucleotides (QTNs) able to differentiate the resistant versus susceptible (percentage of favorable alleles and disease score in parentheses). CIM, CIMMYT; IND, India; NPL, Nepal.

We found that the favorable and non‐favorable alleles of only 66 QTNs out of 91 could differentiate the mean values of AUDPC for more than three environments, including the pooled over the years, at a *p*‐value < 0.001. These QTLs' mean differentiating ability indicates a slightly high solitary contribution to trait expression. Therefore, these QTLs were considered to understand the frequency of favorable alleles and their effect on STB resistance. Over the population, the frequency of favorable alleles was ∼42.25%. The highest percentage of favorable alleles were present in genotypes of CIMMYT origin (53.61%) followed by India (47.92%), Nepal (42.42%), and Bangladesh (25.03%), and mean AUDPC pattern was resembling the percentage of favorable alleles present in the subpopulations, which was lowest in genotypes of CIMMYT (∼801.6), followed by India (871.6), Nepal (905.4), and Bangladesh (1116.0). Figure 9 illustrates the significant negative relationship between the AUDPC and the number of favorable alleles in the genotypes. The negative relationship was observed for 2019 and 2020 and pooled over the years >0.83 at *p*‐value < 2.2 E‐16, and for the year 2021, R = −0.74 at *p*‐value < 2.2 E‐16. This relationship is further reconfirmed by the decrease in the mean value of AUDPC over the gradual staking of the favorable alleles in the genotypes across the years (Figure 10). The topmost 25 resistant genotypes having the lower for STB were selected based on gBLUP‐based GEBV (Table 4). The list of resistant sources was dominated by genotypes coming from the CIMMYT program. However, genotypes from India and Nepal origin were also qualified. The CIM20 (BAVIS/3/ATTILA/BAV92//PASTOR/5/CROC1/AE.SQUARROSA(205)//BORL95/3/PRL/SARA//TSI/VEE#5/4/FRET2) carrying the ∼80.2% favorable alleles was having the lowest AUDPC across the years along with shorter PH and DH (Table 4). Therefore, this genotype having the true resistance (least chances of disease escape) is worth breeding for STB resistance because lower PH and DH are preferable traits in the current wheat improvement strategy of CIMMYT to control the lodging and terminal heat stress. The other genotypes, namely, CIM56, CIM57, CIM18, CIM44, NPL28, and IND14, can be utilized as resistant sources for the direct breeding program without the fear of linkage drag of longer DH and PH (Table 4). Neighbor‐joining‐based hierarchical cluster analysis considering the only QTNs that were able to differentiate the mapping panel based on mean indicated that genotypes fall in the different clusters carrying the different set of genes governing the STB resistance (Figure 10). Like, genotypes, namely, CIM88, CIM89, and CIM90, carrying a large percentage of favorable alleles, ∼75% with a high level of STB resistance, and fell together in different sub‐clusters other than the cluster of genotypes, that is, CIM20 and CIM56 having the highest percentage of favorable alleles (>75%) and STB resistance (Figure 11).

### 3.9 Putative candidate gene identification and functional annotation

Putative CGs were identified with in silico mining of reference genome, that is, IWGSC RefSeq v1.1, using the Ensembl Plant database for all MTA covering the flanking region of ±LD block size. Several putative CGs were identified during the search. They were sorted into 77 putative CGs for repetitive QTNs and 104 for unique CGs based on their involvement in disease response as per literature search and domain type (Table 3; Table S3). These genes are involved in different biological activities like protein kinase‐like domain, Cytochrome P450, leucine‐rich repeat (LRR) domain superfamily, F‐box domain, and Homeobox‐like domain superfamily (Mahboubi et al., 2022). Cell‐surface localized receptor‐like kinases (RLK) and receptor‐like proteins are divided into several different sub‐families including the LRR‐RLK and the wall‐associated kinases (WAK), nucleotide‐binding and LRR domains (NLRs), and cysteine‐rich receptor‐like kinase (CRK) (Saintenac et al., 2018). Some putative CGs falling in the LD block of repetitive QTNs related to disease resistance have been given below. For instance, putative CGs like TraesCS2D02G277300, TraesCS2D02G278100, and TraesCS2D02G276600 in Q.CIM.stb.2DL.2; TraesCS2A02G380500 and TraesCS2A02G381400 in Q.CIM.stb.2AL.5; TraesCS3A02G302600, TraesCS3A02G301500, and TraesCS3A02G301600 in Q.CIM.stb.3AL.2 to Q.CIM.stb.3AL.5; TraesCS5A02G372100 and TraesCS5A02G368200 in Q.CIM.stb.5AL.5; TraesCS5A02G394400, TraesCS5A02G399100, TraesCS5A02G399200, TraesCS5A02G396800, TraesCS5A02G396300, and TraesCS5A02G397700 linked to Q.CIM.stb.5AL.13; TraesCS6A02G005000, TraesCS6A02G008700, and TraesCS6A02G005200 linked to Q.CIM.stb.6AS.1; TraesCS6A02G045100, TraesCS6A02G048700, and TraesCS6A02G047200 linked to Q.CIM.stb.6AS.2; and TraesCS7B02G407900 and TraesCS7B02G410100 linked with Q.CIM.stb.7BL.1 were located in the LD block of the genomic region of respective QTNs (Table 3). Likewise, unique QTNs concerning years and models surpassing the strict criteria of the Bonferroni corrections threshold also harbor the CGs having the relevant molecular and biological function (Table S3).

**TABLE 3:** Putative candidate genes covering the flanking region of repetitive quantitative trait nucleotides (QTNs).

QTNs	Position	Gene ID	Chromosome position and size (bp)	Domain/family	Description	Biological function
Q.CIM.stb.1AL.3	527955152 ± 1869261	TraesCS1A02G338200	chr1A:527950264‐527955845(+)	Glycosyltransferase 2NA family	Probable alpha, alpha‐trehalose‐phosphate synthase (UDP‐forming) 7 (UniProtKB/Swiss‐Prot:Q9LMI0)	Trehalose biosynthetic process
		TraesCS1A02G338700	chr1A:528102140‐528103155(−)	NA	VQ motif‐containing protein 4 (UniProtKB/Swiss‐Prot:Q5M750)	Negative regulation of cellular defense response (GO:0051245)
		TraesCS1A02G338600	chr1A:528064927‐528067271(−)	Phosphatidylethanolamine‐binding protein family	Protein RICE FLOWERING LOCUS T 1 (UniProtKB/Swiss‐Prot:Q8VWH2)	Regulation of timing of transition from vegetative to reproductive phase (GO:0048510)
Q.CIM.stb.1AL.4	547215683 ± 1869261	TraesCS1A02G370100	chr1A:547391832‐547395833(−)	Zinc‐containing alcohol dehydrogenase family	Alcohol dehydrogenase 3 (UniProtKB/Swiss‐Prot:P10848)	Alcohol dehydrogenase (NAD) activity (GO:0004022)
		TraesCS1A02G366900	chr1A:545647584‐545649340(−)	Protein kinase superfamily. Ser/Thr protein kinase family	Mitogen‐activated protein kinase kinase kinase 17 (UniProtKB/Swiss‐Prot:O80888)	Abscisic acid‐activated signaling pathway (GO:0009738)
		TraesCS1A02G370400	chr1A:547863784‐547865124(+)	AP2/ERF transcription factor family. ERF subfamily	Ethylene‐responsive transcription factor ERF109 (UniProtKB/Swiss‐Prot:Q9SZ06)	Ethylene‐activated signaling pathway (GO:0009873)
		TraesCS1A02G369300	chr1A:547016778‐547022195(−)	Coiled coil	Protein HEAT INTOLERANT 4 (UniProtKB/Swiss‐Prot:A2RVJ8)	Regulation of cellular response to heat (GO:1900034)
		TraesCS1A02G371900	chr1A:548674143‐548676077(+)	Threonine synthase family	Threonine synthase, chloroplastic (UniProtKB/Swiss‐Prot:Q9MT28)	Threonine biosynthetic process (GO:0009088)
		TraesCS1A02G366900	chr1A:545647584‐545649340(−)	Protein kinase superfamily. Ser/Thr protein kinase family	Mitogen‐activated protein kinase kinase kinase 17 (UniProtKB/Swiss‐Prot:O80888)	Stress‐activated protein kinase signaling cascade (GO:0031098)
Q.CIM.stb.1AL.5	563183474 ± 1869261	TraesCS1A02G397500	chr1A:563183471‐563184200(−)	Signal_peptidase C1 family	Ubiquitin‐40S ribosomal protein S27a (UniProtKB/Swiss‐Prot:P0CG86)	Protein ubiquitination with ligase binding; structural constituent of ribosome
		TraesCS1A02G396000	chr1A:561967125‐561976767(+)	Protein kinase superfamily. CMGC Ser/Thr protein kinase family. CDC2/CDKX subfamily	Guanylate‐binding protein 2 (UniProtKB/Swiss‐Prot:P32456)	Type I interferon signaling pathway (GO:0060337)
Q.CIM.stb.1BL.4	635831373 ± 2436999	TraesCS1B02G408200	chr1B:635920024‐635926285(+)	Redox‐active center_ glutaredoxin family. CC‐type subfamily	Asparagine synthetase (glutamine‐hydrolyzing) 2 (UniProtKB/Swiss‐Prot:Q43011)	Asparagine synthase (glutamine‐hydrolyzing) activity (GO:0004066)
		TraesCS1B02G410500	chr1B:637387602‐637388581(−)	Coiled coil_TRAFAC class dynamin‐like GTPase superfamily	Putative cyclin‐dependent kinase F‐2 (UniProtKB/Swiss‐Prot:Q2QSL4)	Cyclin‐dependent protein serine/threonine kinase activity (GO:0004693)
		TraesCS1B02G409100	chr1B:636725519‐636727081(−)	Zinc‐finger_ubiquitin family	Caricain (UniProtKB/Swiss‐Prot:P10056)	Cysteine‐type peptidase activity (GO:0008234)
		TraesCS1B02G404700	chr1B:633715202‐633715792(+)	Zinc‐finger, SINA (Seven in absentia) family	ATP synthase subunit a, chloroplastic (UniProtKB/Swiss‐Prot:A1EA02)	–
		TraesCS1B02G405400	chr1B:633726802‐633727122(‐)	Peptidase C14B family	NAD(P)H‐quinone oxidoreductase subunit 4L, chloroplastic (UniProtKB/Swiss‐Prot:P69379)	Photosynthesis, light reaction (GO:0019684)
Q.CIM.stb.2AL.5	623943280 ± 1869261	TraesCS2A02G380500	chr2A:623941108‐623946240(−)	Protein kinase superfamily. Ser/Thr protein kinase family	UDP‐xylose transporter 1 (UniProtKB/Swiss‐Prot:F4IHS9)	Regulation of pectin biosynthetic process (GO:1900030)
		TraesCS2A02G380600	chr2A:624039136‐624042480(−)	Signal_peptidase M2NA family.	Probable cytokinin riboside 5′‐monophosphate phosphoribohydrolase LOGL6 (UniProtKB/Swiss‐Prot:Q0JBP5)	Cytokinin biosynthetic process (GO:0009691)
		TraesCS2A02G381400	chr2A:624941477‐624944012(−)	Transmembrane helix_TPT transporter family. TPT (TC 2.A.7.9) subfamily	Wall‐associated receptor kinase 5 (UniProtKB/Swiss‐Prot:Q9LMN7)	Cell surface receptor signaling pathway (GO:0007166)
		TraesCS2A02G380500	chr2A:623941108‐623946240(−)	Protein kinase superfamily. Ser/Thr protein kinase family	UDP‐xylose transporter 1 (UniProtKB/Swiss‐Prot:F4IHS9)	Regulation of pectin biosynthetic process (GO:1900030)
Q.CIM.stb.2BL.3	700208622 ± 2436999	TraesCS2B02G505600	chr2B:700204619‐700209000(+)	Transit peptide_ transketolase family. DXPS subfamily	Importin subunit beta‐1 (UniProtKB/Swiss‐Prot:Q9FJD4)	Small GTPase binding; intracellular protein transport; intracellular anatomical structure
		TraesCS2B02G504700	chr2B:699039998‐699049444(+)	Transmembrane helix_complex I subunit 4L family	Metacaspase‐4 (UniProtKB/Swiss‐Prot:O64517)	Positive regulation of programmed cell death (GO:0043068)
Q.CIM.stb.2BL.6	732344454 ± 2436999	TraesCS2B02G536900	chr2B:732342397‐732344660(+)	Leucine‐rich repeat_disease resistance NB‐LRR family	Putative disease resistance protein At1g50180 (UniProtKB/Swiss‐Prot:Q9SX38)	Defense response with ADP binding
		TraesCS2B02G536400	chr2B:732017353‐732020895(−)	Wall‐associated receptor kinase‐like 9	Wall‐associated receptor kinase‐like 9 (UniProtKB/Swiss‐Prot:Q9C9L5)	Cell surface receptor signaling pathway (GO:0007166)
		TraesCS2B02G538200	chr2B:733713197‐733716418(−)	Leucine‐rich repeat_ protein kinase superfamily	Probable inactive receptor kinase At1g48480 (UniProtKB/Swiss‐Prot:Q9LP77)	N/A
		TraesCS2B02G534200	chr2B:730562324‐730569849(−)	NADPH–cytochrome P45NA reductase family	NADPH–cytochrome P450 reductase (UniProtKB/Swiss‐Prot:Q05001)	N/A
		TraesCS2B02G539300	chr2B:734452993‐734457716(−)	Transit peptide_Protein COFACTOR ASSEMBLY OF COMPLEX C SUBUNIT B CCB4, chloroplastic	Protein COFACTOR ASSEMBLY OF COMPLEX C SUBUNIT B CCB4, chloroplastic (UniProtKB/Swiss‐Prot:Q6NQK9)	Cytochrome b6f complex assembly (GO:0010190)
Q.CIM.stb_dh.2BL.9	784545663 ± 2436999	TraesCS2B02G602000	chr2B:784543948‐784548017(+)	Leucine‐rich repeat_ protein kinase superfamily. Ser/Thr protein kinase family	Receptor‐like kinase TMK3 (UniProtKB/Swiss‐Prot:Q9SIT1)	Protein phosphorylation with ATP binding; integral component of membrane
		TraesCS2B02G603000	chr2B:785208360‐785210039(+)	Transmembrane_NDR1/HIN1‐like protein 1	NDR1/HIN1‐like protein 1 (UniProtKB/Swiss‐Prot:Q9SRN0)	Defense response to virus (GO:0051607)
Q.CIM.stb.2DL.2	349559679 ± 2204775	TraesCS2D02G278300	chr2D:349559498‐349562247(−)	Protein XRI1	Protein XRI1 (UniProtKB/Swiss‐Prot:Q6NLW5)	Female meiotic nuclear division (GO:0007143)
		TraesCS2D02G277300	chr2D:347466158‐347476481(−)	U‐box domain‐containing protein 9	U‐box domain‐containing protein 9 (UniProtKB/Swiss‐Prot:Q9SRT0)	Abscisic acid‐activated signaling pathway (GO:0009738)
		TraesCS2D02G276300	chr2D:345835751‐345870707(−)	Transmembrane_ cation transport ATPase (P‐type) (TC 3.A.3) family. Type IV subfamily	Phospholipid‐transporting ATPase 2 (UniProtKB/Swiss‐Prot:P98205)	Phospholipid translocation (GO:0045332)
		TraesCS2D02G276600	chr2D:346099542‐346101686(+)	Zinc‐containing alcohol dehydrogenase family. Quinone oxidoreductase subfamily	Chloroplast envelope quinone oxidoreductase homolog (UniProtKB/Swiss‐Prot:Q9SV68)	
		TraesCS2D02G278100	chr2D:348723583‐348725398(−)	Glycosyl hydrolase 18 family. Chitinase class V subfamily	Class V chitinase CHIT5b (UniProtKB/Swiss‐Prot:A0A072UR65)	
Q.CIM.stb_dh.2DL.3	590930925 ± 2204775	TraesCS2D02G493400	chr2D:590929808‐590931652(−)	Iron/ascorbate‐dependent oxidoreductase family	Naringenin,2‐oxoglutarate 3‐dioxygenase (UniProtKB/Swiss‐Prot:P28038)	Metal ion binding
		TraesCS2D02G491200	chr2D:589972466‐589978865(+)	ARF family	Auxin response factor 11 (UniProtKB/Swiss‐Prot:Q8S983)	Auxin‐activated signaling pathway (GO:0009734)
		TraesCS2D02G493600	chr2D:591019752‐591023458(+)	SHMT family	Serine hydroxymethyltransferase 4 (UniProtKB/Swiss‐Prot:O23254)	Cellular response to tetrahydrofolate (GO:1904482)
		TraesCS2D02G495700	chr2D:592410871‐592411878(+)	bZIP family	Protein FD (UniProtKB/Swiss‐Prot:Q84JK2)	Photoperiodism (GO:0009648)
		TraesCS2D02G495300	chr2D:592092285‐592092689(+)	ARG7 family	Indole‐3‐acetic acid‐induced protein ARG7 (UniProtKB/Swiss‐Prot:P32295)	Response to auxin (GO:0009733)
		TraesCS2D02G490500	chr2D:589315866‐589318638(−)	Cytochrome P45NA family	Cytochrome P450 84A1 (UniProtKB/Swiss‐Prot:Q42600)	Response to UV‐B (GO:0010224)
Q.CIM.stb.3AL.2	535194590 ± 1869261	TraesCS3A02G301500	chr3A:535190771‐535194807(+)	Isopentenyl phosphate kinase family	Isopentenyl phosphate kinase (UniProtKB/Swiss‐Prot:Q8H1F7)	Terpenoid biosynthetic process with ATP binding; cytosol
		TraesCS3A02G302600	chr3A:536626275‐536629711(−)	EXO7NA family	Exocyst complex component EXO70E2 (UniProtKB/Swiss‐Prot:Q9FNR3)	Defense response by callose deposition (GO:0052542)
Q.CIM.stb.3AL.3 to Q.CIM.stb.3AL.5	537508944 ± 1869261	TraesCS3A02G301600	chr3A:535195059‐535199817(+)	Coiled coil_	Clp protease adapter protein ClpF, chloroplastic (UniProtKB/Swiss‐Prot:Q67Y99)	Chloroplastic endopeptidase Clp complex (GO:0009840)
		TraesCS3A02G302600	chr3A:536626275‐536629711(−)	EXO7NA family	Exocyst complex component EXO70E2 (UniProtKB/Swiss‐Prot:Q9FNR3)	Defense response by callose deposition (GO:0052542)
		TraesCS3A02G302100	chr3A:535323539‐535326920(+)	GST superfamily. Phi family	Glutathione S‐transferase 1 (UniProtKB/Swiss‐Prot:P12653)	Glutathione metabolic process (GO:0006749)
		TraesCS3A02G303200	chr3A:537507387‐537509322(+)	RGP family	UDP‐arabinopyranose mutase 1 (UniProtKB/Swiss‐Prot:Q8H8T0)	Plant‐type cell wall organization or biogenesis
		TraesCS3A02G315100	chr3A:556354721‐556361160(−)	Protein kinase superfamily. Ser/Thr protein kinase family	G‐type lectin S‐receptor‐like serine/threonine‐protein kinase B120 (UniProtKB/Swiss‐Prot:O81906)	Recognition of pollen; protein phosphorylation; ATP binding; integral component of membrane
Q.CIM.stb_dh.3AL.6	733995575 ± 1869261	TraesCS3A02G516400	chr3A:733991852‐734002806(+)	ABC transporter superfamily.	ABC transporter D family member 1 (UniProtKB/Swiss‐Prot:Q94FB9)	ABC‐type transporter activity; integral component of membrane
		TraesCS3A02G517800	chr3A:734838436‐734843412(+)	Diphosphomevalonate decarboxylase family	Diphosphomevalonate decarboxylase MVD2, peroxisomal (UniProtKB/Swiss‐Prot:F4JCU3)	Diphosphomevalonate decarboxylase activity (GO:0004163)
		TraesCS3A02G513700	chr3A:732769617‐732780010(+)	Zinc‐finger_ TFIIE alpha subunit family	General transcription factor IIE subunit 1 (UniProtKB/Swiss‐Prot:Q557M8)	–
		TraesCS3A02G513600	chr3A:732698012‐732701223(+)	Zinc‐finger_class V‐like SAM‐binding methyltransferase superfamily	Probable Histone‐lysine N‐methyltransferase ATXR5 (UniProtKB/Swiss‐Prot:B9RU15)	Histone acetyltransferase activity (GO:0004402)
Q.CIM.stb.4BL.6	657172009 ± 2436999	TraesCS4B02G371700	chr4B:657171824‐657176698(‐)	Major facilitator superfamily. Sugar transporter (TC 2.A.1.1) family	Hexose carrier protein HEX6 (UniProtKB/Swiss‐Prot:Q07423)	Carbohydrate transmembrane transporter activity; integral component of membrane
		TraesCS4B02G375300	chr4B:659481853‐659485452(−)	EGF‐like domain_protein kinase superfamily. Ser/Thr protein kinase family	G‐type lectin S‐receptor‐like serine/threonine‐protein kinase B120 (UniProtKB/Swiss‐Prot:O81906)	Recognition of pollen (GO:0048544)
		TraesCS4B02G372100	chr4B:657243113‐657246017(+)	Leucine‐rich repeat_protein kinase superfamily. Ser/Thr protein kinase family	Probable leucine‐rich repeat receptor‐like protein kinase At1g35710 (UniProtKB/Swiss‐Prot:Q9LP24)	Hormone‐mediated signaling pathway (GO:0009755)
Q.CIM.stb.5AL.5	570714677 ± 1869261	TraesCS5A02G372100	chr5A:570712990‐570718437(+)	Coiled coil_ethyl‐CpG‐binding domain (MBD)	Methyl‐CpG‐binding domain‐containing protein 10 (UniProtKB/Swiss‐Prot:Q9XI36)	Methyl‐CpG‐binding domain; Nucleus
		TraesCS5A02G369800	chr5A:569677389‐569678193(+)	Plant dehydrin family	Dehydrin DHN2 (UniProtKB/Swiss‐Prot:P12952)	Response to water (GO:0009415)
Q.CIM.stb.5AL.13	591319097 ± 1869261	TraesCS5A02G396500	chr5A:591313846‐591320402(+)	NA	NA	
		TraesCS5A02G399000	chr5A:592681433‐592687421(−)	NA	Protein arginine N‐methyltransferase 2 (UniProtKB/Swiss‐Prot:Q2TZM9)	Protein‐arginine N‐methyltransferase activity (GO:0016274)
		TraesCS5A02G399100	chr5A:592832477‐592833646(+)	Peroxidase family. Classical plant (class III) peroxidase subfamily	Peroxidase 57 (UniProtKB/Swiss‐Prot:Q43729)	Hydrogen peroxide catabolic process (GO:0042744)
		TraesCS5A02G399200	chr5A:592833978‐592834289(+)	Transmembrane	NAD(P)H‐quinone oxidoreductase subunit 3, chloroplastic (UniProtKB/Swiss‐Prot:P26303)	Photosynthesis, light reaction (GO:0019684)
		TraesCS5A02G395300	chr5A:590664252‐590669685(+)	NA	Cyclic nucleotide‐gated ion channel 2 (UniProtKB/Swiss‐Prot:O65718)	Calcium ion import (GO:0070509)
		TraesCS5A02G396800	chr5A:591442651‐591446929(+)	WRKY domain	WRKY transcription factor WRKY24 (UniProtKB/Swiss‐Prot:Q6B6R4)	Gibberellic acid mediated signaling pathway (GO:0009740)
		TraesCS5A02G396300	chr5A:591133078‐591142446(+)	Protein kinase superfamily. TKL Ser/Thr protein kinase family. RAF subfamily	Serine/threonine‐protein kinase EDR1 (UniProtKB/Swiss‐Prot:Q9FPR3)	Negative regulation of abscisic acid‐activated signaling pathway (GO:0009788)
		TraesCS5A02G397700	chr5A:591992402‐591994192(−)	Cytochrome P45NA family	Cytochrome P450 81Q32 (UniProtKB/Swiss‐Prot:W8JMU7)	Oxidoreductase activity, acting on paired donors (GO:0016705)
Q.CIM.stb.6AS.1	2178375 ± 1869261	TraesCS6A02G005600	chr6A:2177764‐2180753(−)	NA	Tricetin 3′,4′,5′‐O‐trimethyltransferase (UniProtKB/Swiss‐Prot:Q38J50)	Protein dimerization activity
		TraesCS6A02G005000	chr6A:1942409‐1948879(+)	Transmembrane_Protein ENHANCED DISEASE RESISTANCE 2‐like	Protein ENHANCED DISEASE RESISTANCE 2 (UniProtKB/Swiss‐Prot:F4JSE7)	–
		TraesCS6A02G001700	chr6A:811604‐816032(+)	NA	Protein SUPPRESSOR OF NPR1‐1 CONSTITUTIVE 4 (UniProtKB/Swiss‐Prot:D7SFH9)	Cellular defense response (GO:0006968)
		TraesCS6A02G005200	chr6A:1972751‐1975657(+)	Coiled coil_ TRAFAC class TrmE‐Era‐EngA‐EngB‐Septin‐like GTPase superfamily	Immune‐associated nucleotide‐binding protein 9 (UniProtKB/Swiss‐Prot:F4HT21)	Negative regulation of defense response to bacterium, incompatible interaction (GO:1902478)
Q.CIM.stb.6AS.2	23410186 ± 1869261	TraesCS6A02G045100	chr6A:23409174‐23410981(+)	Transcription factor ILR3‐like	Transcription factor ILR3 (UniProtKB/Swiss‐Prot:Q9FH37)	DNA‐binding transcription factor activity with ILR 3 transcription factor
		TraesCS6A02G048700	chr6A:24838784‐24842716(+)	Glutamate‐gated ion channel (TC 1.A.1NA.1) family	Glutamate receptor 3.1 (UniProtKB/Swiss‐Prot:Q7XP59)	Signaling receptor activity (GO:0038023)
		TraesCS6A02G047200	chr6A:23895381‐23896865(−)	Signal_ peroxidase family. Classical plant (class III) peroxidase subfamily	Peroxidase 70 (UniProtKB/Swiss‐Prot:A5H452)	Peroxidase activity (GO:0004601)
Q.CIM.stb.7AL.3	672042193 ± 1869261	TraesCS7A02G479900	chr7A:672041945‐672046257(−)	NA	rRNA 2′‐O‐methyltransferase fibrillarin 2 (UniProtKB/Swiss‐Prot:Q94AH9)	RNA binding; rRNA processing
		TraesCS7A02G480100	chr7A:672069735‐672075854(+)	NA	Cation‐transporting ATPase HMA5 (UniProtKB/Swiss‐Prot:A0A0P0X004)	Copper transmembrane transporter activity, phosphorylative mechanism (GO:0043682)
Q.CIM.stb.7BL.1	678030488 ± 2436999	TraesCS7B02G407900	chr7B:677303946‐677304822(+)	Basic helix‐loop‐helix (bHLH) domain	Transcription factor IBH1‐like 1 (UniProtKB/Swiss‐Prot:Q9M0B9)	Gibberellic acid mediated signaling pathway (GO:0009740)
		TraesCS7B02G408600	chr7B:677881629‐677883530(−)	NA	Two‐component response regulator ORR22 (UniProtKB/Swiss‐Prot:Q5SML5)	Cytokinin‐activated signaling pathway (GO:0009736)
		TraesCS7B02G410700	chr7B:679681774‐679684199(−)	SWEET sugar transporter family	Bidirectional sugar transporter SWEET6b (UniProtKB/Swiss‐Prot:A2WSD3)	Sugar transmembrane transporter activity (GO:0051119)

Abbreviation: CIm, CImmYT.

## 4 DISCUSSION

STB is one of the most important foliar diseases across continents. In the present study, we found significant differences in the main effects of genotype (G) and year (Y) and G × Y interaction for STB, PH, and DH. The high correlation over the years in this study aligns with our previous reports published for a biparental (He et al., 2021). Due to variations in moisture, temperature, and pathogen population, STB responses varied year to year (Riaz et al., 2020), which was also observed in our study where STB infection was the highest in 2019 than in the other 2 years. The moderately high broad sense heritability of STB infection over years (h^2^ = 0.62 to 0.84) indicated that the heritable genetic factors largely determine STB resistance and that reliable QTLs can be identified with GWAS analysis. Our findings support fairly high STB response heritability reported by other studies (Goudemand et al., 2013; Juliana et al., 2017; Kollers et al., 2013; Yang et al., 2022). The normal distribution of adjusted STB (BLUP) values suggests that multiple minor genes were responsible for STB resistance in the panel.

Based on Pearson's pairwise correlation coefficient, STB severity had a significant negative correlation with DH and PH over years, which suggests that longer DH and taller PH may help the plants to escape the disease, or there were pleiotropic effects of the phenological genes or their tightly linkage with STB resistance genes (Goudemand et al., 2013; He et al., 2021; Juliana et al., 2017; Kollers et al., 2013; Yang et al., 2022). The identification of QTNs for multiple traits, like Q.CIM.stb_dh.1AS.1 on 1AS and Q.CIM.stb_ph.7AL.4 on 7AL, corroborates the reported associations of the STB response with DH and PH.

### 4.1 Populations stratification and marker trait associations

The mapping panel was subdivided into two sub‐populations, with the small one comprising genotypes of CIMMYT origin and the rest forming the big sub‐population. Further analysis of the pedigree information indicated that the grouping was mostly based on pedigree lineage, as well as geographical origin, in agreement with previous studies (Gorafi et al., 2018).

In our study, 91 QTNs were associated with STB resistance over the three GWAS algorithm models and years. Of them, 23 QTNs were repetitive across the models or years, while 68 QTNs were unique due to the strong effect of Y and G × Y interaction on the expression of the STB resistance. This agrees with the previous reports that STB resistance is quantitatively inherited and highly influenced by the environmental factor (Dreisigacker et al., 2015) and genotype by environment interaction (Gerard et al., 2017; Riaz et al., 2020). The repetitive QTNs over the years and models are more reliable in representing the genetic architecture of STB resistance and converting them into KASP assays for the introgression of the STB resistance. However, the unique QTNs identified in single model and years could also be used due to their ability to differentiate the GWAS panel into resistant versus susceptible groups over the GEBV with gBLUP. Example of the former type includes Q.CIM.stb.2DL.2 that is located near the centromere on 2D, which was able to differentiate the mapping panel into resistant and susceptible groups over the years. This QTN might be novel because of its unique physical position without co‐localization of previously reported STB genes or QTLs (Figure 6). Likewise, two additional repetitive QTNs, Q.CIM.stb.2AL.5 and Q.CIM.stb.7BL.1, were also able to divide the mapping panel into two groups and might be the novel.

The repeated QTNs on the long arm of 2B were co‐localized with additional unique QTNs identified in our study, as well as previously known STB genes, QTNs, and MQTLs, forming a QTL hotspot governing STB resistance (Figure 6). This region from 700.20 to 784.54 Mb contains Stb9 (Chartrain et al., 2009) and MQTL8 (Goudemand et al., 2013) for APR; QTLs, namely, QStb.wai.2B.1 and QTL‐2BL, for multi‐stage resistance (Aouini, 2018; Yang et al., 2022); and QTNs for APR, Q.CIM.stb.2BL.3 within this region differentiated the mapping panel into resistant and susceptible groups, but not Q.CIM.stb.2BL.6. Thus, Q.CIM.stb.2BL.6 might represent a minor gene on STB resistance only when combined with other genes, just like the *Lr46* gene that works better with other slow rusting genes (Singh et al., 1998).

Similarly, a QTL complex having the QTNs from Q.CIM.stb.5AL.2 to Q.CIM.stb.5AL.14 spanning from 549 to 591 Mb was located on the long arm of 5A, in which Q.CIM.stb.5AL.5 and Q.CIM.stb.5AL.13 were the repetitive QTNs. Some of the previously reported genes like Stb17 for APR, QStb.wai.5A.1 for multi‐stage resistance (Yang et al., 2022), Qstb.iau‐10 for APR (Mahboubi et al., 2022), and QTL associated with Tdurum_contig10210_425 (Kidane et al., 2017) were co‐localized within this QTL hotspot. It is noteworthy, however, that this region might be associated with disease escape because a QTN for DH, Q.CIM.dh.5AL.5 at 598 Mb, was also identified in this region.

Q.CIM.stb.6AS.1 and Q.CIM.stb.6AS.2 on the short arm of 6A were co‐localized with QStb.teagasc‐6A.1 (Riaz et al., 2020), MQTL20 (1.20–25.80 Mb) for multi‐stage STB resistance (Goudemand et al., 2013), Qstb.iau‐14 (Mahboubi et al., 2022), Stb15 (Arraiano et al., 2007), QStb.wai.6A.1 (Yang et al., 2022), and a major QTL linked to Excalibur_c12085_276 (Würschum et al., 2017). Q.CIM.stb.2AS.2 fell within the MQTL4 responsible for APR (Goudemand et al., 2013), which is only 0.1 cM away from the marker, *Xwmc177* linked to *Ppd‐1A* (Wilhelm et al., 2009). The remaining repetitive and unique QTNs identified in this study were located within LD blocks with reported QTLs or *Stb* genes; for example, Q.CIM.stb.4AL.7 and Stb12 were within a LD block (∼0.92 Mb) with a physical distance of 0.80 Mb, and Q.CIM.stb.2BL.3 to Q.CIM.stb.2BL.8 was found to be close to Stb9. Refer to Table S6 for more such examples.

The QTNs, namely, Q.CIM.stb.2AS.1 and Q.CIM.stb.2AS.2, were very close to the 2NS/2AS translocation region (0–35.4 Mb) that were associated with wheat blast resistance (He et al., 2021). Although no marker within this region was identified to be significant in GWAS for STB resistance, the 2AS/2NS translocation segment did exhibit significant phenotypic effects in 2 of the 3 years, implying some minor genes within the segment conferred resistance against STB. This is an encouraging finding because the segment has already been associated with many favorable traits like lodging tolerance, high yielding, resistance to rusts, nematodes, spot blotch, and wheat blast (Bariana & McIntosh, 1993; Doussinault et al., 1983; Gao et al., 2021; Jahier et al., 2001; Juliana et al., 2020, 2018, 2022; Williamson et al., 2013).

Great caution should be taken on the utilization of QTNs for both STB and DH/PH because they are often associated with disease escape (Riaz et al., 2020). Examples of such QTNs include Q.CIM.stb_dh.2DL.3 and Q.CIM.stb_dh.2BL.9 for both STB and DH, and Q.CIM.stb_ph.7AL.4, Q.CIM.stb_ph.2AL.3 and Q.CIM.stb_ph.1BS.1 for both STB and PH. At these loci, low STB infection was always associated with late heading or tall stature, implying that disease escape, rather than close linkage or pleiotropic, was the underlying mechanism (Riaz et al., 2020). Interestingly, Q.CIM.stb_ph.2AL.3 located on 2A had the complete LD (*R* ^2^ = 1.0) with Q.CIM.stb.4AL.4 located on 4A. Therefore, there is a very high chance these QTLs inherit together in an LD block, and this LD block would have formed with selection or evolutionary constraints over the past and works in complementation with each other.

### 4.2 Resistant germplasm selection and effect of favorable alleles over the STB resistance

The high percentage of favorable alleles in CIMMYT germplasm used in our study indicates the systematic and continuous accumulation of favorable alleles with minor effects over time. The significant negative relationship between the number of favorable alleles and AUDPC value and the decrease in the mean value of AUDPC over the gradual staking of the favorable alleles (Figures 8 and 9) indicate quantitative genetic control of STB resistance as previously reported (Langlands‐Perry et al., 2022; Louriki et al., 2021; Mahboubi et al., 2022; Mekonnen et al., 2021; Riaz et al., 2020; Yang et al., 2022). In our study, genotypes with high percentage of favorable alleles, as well as good resistance in field trials were identified, including CIM20, CIM56, CIM57, CIM18, CIM44, NPL28, and IND14, which can be used as resistant sources for direct breeding without linkage drag of longer DH and PH (Table 4).

**TABLE 4:** Resistant genotypes with their STB resistance response (gBLUP values) and percentage of favorable/resistant alleles.

Taxa	DH_POOL	PH_POOL	STB2019	STB2020	STB2021	STB_POOL	2NS/2AS	Favorable alleles	Favorable alleles (%)	Parentage
CIM15	69.0	105.1	634.4	726.1	713.4	671.0	2NS	45	68.18	TOB/ERA//TOB/CNO67/3/PLO/4/VEE#5/5/KAUZ/6/FRET2/7/VORB/8/MILAN/KAUZ//DHARWARDRY/3/BAV92
CIM18	69.1	100.1	615.4	641.0	727.5	625.6	2AS	45	68.18	KIRITATI//HUW234+LR34/PRINIA/3/CHONTE/5/PRL/2*PASTOR/4/CHOIX/STAR/3/HE1/3*CNO79//2*SERI
CIM20	66.6	100.8	347.7	397.5	563.6	339.9	2AS	53	80.30	BAVIS/3/ATTILA/BAV92//PASTOR/5/CROC_1/AE.SQUARROSA(205)//BORL95/3/PRL/SARA//TSI/VEE#5/4/FRET2
CIM26	68.6	105.0	439.9	551.5	641.7	459.3	2AS	37	56.06	VEE/MJI//2*TUI/3/PASTOR/4/BERKUT/5/BAVIS
CIM32	70.4	104.0	775.3	626.8	669.5	651.4	2AS	45	68.18	WHEAR/KUKUNA/3/C80.1/3*BATAVIA//2*WBLL1/4/T.DICOCCONPI94625/AE.SQUARROSA(372)//SHA4/CHIL/5/WHEAR/KUKUNA/3/C80.1/3*BATAVIA//2*WBLL1
CIM34	72.1	107.0	641.4	632.4	695.0	611.0	2NS	31	46.97	MILAN
CIM4	66.8	106.7	606.4	690.9	735.4	644.0	2AS	45	68.18	CROC_1/AE.SQUARROSA(213)//PGO/10/ATTILA*2/9/KT/BAGE//FN/U/3/BZA/4/TRM/5/ALDAN/6/SERI/7/VEE#10/8/OPATA/11/ATTILA*2/PBW65
CIM44	67.3	99.2	690.8	644.1	706.2	659.5	2NS	46	69.70	MUTUS#1
CIM56	67.0	102.6	510.0	511.8	661.1	477.5	2AS	51	77.27	FRNCLN/3/ND643//2*PRL/2*PASTOR/4/FRANCOLIN#1
CIM57	68.7	102.3	560.8	600.3	682.7	559.0	2NS	45	68.18	SWSR22T.B./2*BLOUK#1//WBLL1*2/KURUKU
CIM82	70.3	104.5	606.8	593.5	667.4	548.4	2NS	36	54.55	ORL93320/ER2000
CIM83	75.9	107.1	586.7	590.7	713.7	584.2	2AS	40	60.61	SW91.4903/3/URES/BOW//OPATA/4/SW94.15373
CIM84	72.6	105.6	535.4	627.7	608.3	545.6	2NS	49	74.24	MON/TAN//ROMO96/3/METSO/4/FINSI
CIM88	73.5	107.1	462.3	461.4	574.0	461.8	2NS	49	74.24	FINSI/METSO
CIM89	73.0	106.5	474.8	496.9	574.0	472.2	2NS	49	74.24	FINSI/METSO
CIM90	73.8	107.3	400.5	455.7	550.1	412.2	2NS	50	75.76	FINSI/METSO
CIM92	72.9	105.1	592.1	662.2	724.9	632.7	2AS	47	71.21	GONDO//BAU/MILAN/3/PASTOR
CIM96	74.2	105.1	463.0	572.7	610.4	487.5	2NS	49	74.24	JWS17/7/IAS58/4/KAL/BB//CJ71/3/ALD/5/CNR/6/THB/CEP7780/8/FINSI
IND10	68.6	99.2	694.2	608.5	705.3	667.4	2NS	46	69.70	WH1105
IND14	68.8	107.5	395.2	554.0	653.9	450.7	2AS	36	54.55	K1317 (K0307/K9162)
IND28	66.2	104.7	646.5	597.2	675.3	580.3	2NS	31	46.97	HI1605
IND3	69.9	105.9	709.5	649.3	644.5	622.1	2NS	43	65.15	HD3043
NPL20	66.8	103.9	550.9	746.2	730.9	602.7	2NS	40	60.61	NL1270
NPL22	68.0	103.4	512.6	621.8	712.3	598.1	2AS	45	68.18	NL1226
NPL28	67.8	100.2	771.2	708.5	701.2	664.9	2AS	33	50.00	WK2395

Abbreviations: CIm, CImmYT; DH_POOL, days to heading in across years; gBLUP, genomic best linear unbiased prediction; IND, India; NPL, Nepal; PH_POOL, plant height across years; STB, Septoria tritici blotch; STB2019, Septoria tritici blotch infection in year 2019; STB2020, Septoria tritici blotch infection in year 2020; STB2021, Septoria tritici blotch infection in year 2021; STB_POOL, Septoria tritici blotch infection across years.

### 4.3 In silico functional annotation of putative candidate genes

The genomic regions within the LD block size of significant QTLs/MTA were screened for putative CGs with annotated functions to better understand their functional role (Mahboubi et al., 2022) and strengthen their relevance in designing SNPs‐based PCR assays for marker‐assisted selection, gene cloning, and gene editing. The Cytochrome P450 superfamily proteins of the three CGs, TraesCS2D02G490500, TraesCS5A02G397700, and TraesCS2B02G534200, synthesize hormones, defense compounds, and fatty acids in plants. TraesCS1A02G369300 and TraesCS6A02G005200 proteins are coiled. They contribute to plant heat tolerance (L. C. Wang et al., 2013) and the immune‐associated nucleotide gene family in *Arabidopsis thaliana* (Liu et al., 2008). The glycosyl hydrolase 18 family's putative CG, TraesCS2D02G278100, creates chitinase protein, which stimulates Medicago's defense response with Nod factor hydrolysis (Zhang et al., 2016). TraesCS5A02G399100 and TraesCS6A02G047200, putative CG proteins, are peroxidases and signal between the salicylate and jasmonate pathways in plant defense (Schenk et al., 2000). Calcium‐dependent protein kinases and mitogen‐activated protein kinases signal pathogen identification and plant defense activation (Romeis, 2001). In our investigation, TraesCS1A02G396000, TraesCS1A02G366900, TraesCS2A02G380500, TraesCS5A02G396300, TraesCS1B02G410500, and TraesCS2A02G381600 were protein kinases superfamily CGs in the LD block of respective QTLs. SWEET6b is encoded by TraesCS7B02G410700. Sugars deliver sweet immunity with plant defense and immune signaling molecules (Jeandet et al., 2022). TraesCS2B02G536400 and TraesCS5A02G396800 proteins enter the wall‐associated receptor kinase domain, connected to plant defense. LRR protein superfamily and zinc‐finger superfamily CGs are proteins. These potential genes have protein kinase‐like domains, Cytochrome P450, LRR domain superfamily, F‐box domain, WAK, NLRs, and CRKs.

## 5 CONCLUSION

A panel with wheat genotypes from CIMMYT and South Asia was used to identify STB‐resistant germplasms using gBLUP‐based GEBV and to identify genomic regions affecting traits of interest. In this study, we found 91 STB resistance QTNs across years and models, most of which were located near reported QTL hotspots, MQTLs, or *Stb* genes. However, Q.CIM.stb.2DL.2, Q.CIM.stb_dh.2DL.3, Q.CIM.stb.2AL.5, and Q.CIM.stb.7BL.1 may be novel based on their unique locations. PCR‐based SNP markers like KASP could be useful for tracking and utilization of such QTNs. The top most resistant genotypes having a high percentage of favorable alleles (>50%) could be used in breeding programs as resistant donors. The identified CGs could be targets for future research.

## AUTHOR CONTRIBUTIONS

**Manjeet Kumar**: Formal analysis; investigation; writing—original draft. **Xinyao He**: Conceptualization; data curation; formal analysis; supervision; writing—review and editing. **Sudhir Navathe**: Formal analysis; investigation; writing—review and editing. **Umesh Kamble**: Formal analysis; investigation; writing—review and editing. **Madhu Patial**: Formal analysis; investigation; writing—review and editing. **Pawan Kumar Singh**: Conceptualization; funding acquisition; project administration; resources; supervision; writing—review and editing.

## CONFLICT OF INTEREST STATEMENT

The authors declare no conflicts of interest.

## Supporting information




**Table S1** List of genotypes along with parentage and gBLUP values.
**Table S2** Statistical and genetic parameters for days to heading and plant height over the respective year and pool over years.
**Table S3** The unique quantitative trait nucleotides (QTNs) controlling the Septoria tritici blotch resistance.
**Table S4** The unique quantitative trait nucleotides (QTNs) controlling the plant height (PH) and days to heading (DH).
**Table S5** Unique quantitative trait nucleotides (QTNs) in complete linkage across the genome.
**Table S6** The physical position of reported *Stb* genes and meta‐QTLs.

Supporting Information

## Data Availability

Raw genotypic data are accessible at https://hdl.handle.net/11529/10548559, and raw phenotypic data are available in Table S1.
