# Supplementary material for: Identification of resistance sources and genomic regions regulating Septoria tritici blotch resistance in South Asian bread wheat germplasm
Source: Plant Genome. 2024 Nov 27;18(1):e20531. doi: 10.1002/tpg2.20531 (PMC11726422; doi:10.1002/tpg2.20531)
Supplement: Supplementary file 1 [file TPG2-18-e20531-s002.docx]

**Table S1:** List of genotypes along with parentage and gBLUP values

| **GID** | **TAXA** | **Parentage** | **STB2019** | **STB2020** | **STB2021** | **STB_POOL** | **2NS/2AS** |
| --- | --- | --- | --- | --- | --- | --- | --- |
| 7890088 | BGD1 | BIJOY | 1211.5 | 1142.4 | 891.5 | 1096.0 | 2AS |
| 7890112 | BGD10 | BAW1195 | 1263.9 | 1163.9 | 896.9 | 1138.6 | 2AS |
| 7890115 | BGD11 | BAW1200 | 1261.5 | 1109.3 | 831.5 | 1074.7 | 2AS |
| 7890118 | BGD12 | BAW1202 | 1301.9 | 1216.2 | 849.6 | 1157.0 | 2AS |
| 7890120 | BGD13 | BAW1203 | 1340.9 | 1206.9 | 844.3 | 1148.2 | 2AS |
| 7890122 | BGD14 | BAW1208 | 1173.3 | 1240.4 | 946.7 | 1164.8 | 2AS |
| 7890125 | BGD15 | BAW1209 | 1132.9 | 1196.2 | 855.4 | 1056.3 | 2AS |
| 7890128 | BGD16 | BAW1219 | 1292.3 | 1242.3 | 976.6 | 1218.9 | 2AS |
| 7890134 | BGD17 | BAW1222 | 1289.7 | 1177.7 | 841.0 | 1110.2 | 2AS |
| 7890135 | BGD18 | BAW1249 | 1458.7 | 1278.2 | 959.8 | 1279.6 | 2AS |
| 7890137 | BGD19 | BAW1260 | 1406.7 | 1019.3 | 888.7 | 1164.8 | 2NS |
| 7890090 | BGD2 | PRODIP | 1293.8 | 1225.3 | 924.1 | 1188.9 | 2AS |
| 7890092 | BGD3 | BARIGOM25 | 1066.7 | 866.3 | 805.8 | 871.9 | 2AS |
| 7890096 | BGD4 | BARIGOM26 | 1274.6 | 1236.7 | 912.5 | 1214.2 | 2AS |
| 7890101 | BGD5 | BARIGOM28 | 1013.8 | 1044.8 | 845.5 | 991.8 | 2AS |
| 7890103 | BGD6 | BARIGOM29 | 1220.7 | 1143.3 | 859.1 | 1091.6 | 2AS |
| 7890104 | BGD7 | BARIGOM30 | 1099.3 | 1158.2 | 760.1 | 990.9 | 2AS |
| 7890106 | BGD8 | BAW1182 | 1092.9 | 1089.8 | 814.3 | 989.5 | 2AS |
| 7890110 | BGD9 | BAW1194 | 1428.9 | 1209.5 | 935.9 | 1256.2 | 2AS |
| 6681676 | CIM1 | QUAIU#1/SUP152 | 1002.8 | 842.0 | 801.5 | 880.6 | 2AS |
| 6680982 | CIM10 | FRET2*2/SHAMA//KIRITATI/2*TRCH/3/BAJ#1 | 759.3 | 784.3 | 750.1 | 745.0 | 2AS |
| 6567313 | CIM11 | PAURAQ/5/KIRITATI/4/2*SERI.1B*2/3/KAUZ*2/BOW//KAUZ/6/PAURAQUE#1 | 826.2 | 766.1 | 801.7 | 806.4 | 2AS |
| 6692346 | CIM12 | SOKOLL/3/PASTOR//HXL7573/2*BAU/5/CROC_1/AE.SQUARROSA(205)//BORL95/3/PRL/SARA//TSI/VEE#5/4/FRET2 | 756.7 | 655.1 | 669.9 | 674.1 | 2AS |
| 6679711 | CIM13 | KENYASUNBIRD/2*KACHU | 1124.1 | 905.6 | 859.2 | 960.2 | 2NS |
| 6690981 | CIM15 | TOB/ERA//TOB/CNO67/3/PLO/4/VEE#5/5/KAUZ/6/FRET2/7/VORB/8/MILAN/KAUZ//DHARWARDRY/3/BAV92 | 634.4 | 726.1 | 713.4 | 671.0 | 2NS |
| 6566828 | CIM16 | KIRITATI//2*PRL/2*PASTOR/3/CHONTE/5/PRL/2*PASTOR/4/CHOIX/STAR/3/HE1/3*CNO79//2*SERI | 714.7 | 800.6 | 826.3 | 798.0 | 2AS |
| 6566596 | CIM17 | FRET2*2/BRAMBLING//KIRITATI/2*TRCH/3/FRET2/TUKURU//FRET2 | 880.9 | 856.6 | 825.9 | 883.2 | 2AS |
| 6681164 | CIM18 | KIRITATI//HUW234+LR34/PRINIA/3/CHONTE/5/PRL/2*PASTOR/4/CHOIX/STAR/3/HE1/3*CNO79//2*SERI | 615.4 | 641.0 | 727.5 | 625.6 | 2AS |
| 6568165 | CIM19 | SUP152/FRNCLN | 801.9 | 728.5 | 740.3 | 724.6 | 2AS |
| 6683477 | CIM2 | KIRITATI//HUW234+LR34/PRINIA/3/BAJ#1 | 675.3 | 743.0 | 752.5 | 736.5 | 2NS |
| 6691721 | CIM20 | BAVIS/3/ATTILA/BAV92//PASTOR/5/CROC_1/AE.SQUARROSA(205)//BORL95/3/PRL/SARA//TSI/VEE#5/4/FRET2 | 347.7 | 397.5 | 563.6 | 339.9 | 2AS |
| 6681777 | CIM21 | ND643/2*WBLL1//VILLAJUAREZF2009 | 989.6 | 888.4 | 834.5 | 913.2 | 2AS |
| 6085788 | CIM22 | QUAIU#1 | 1000.0 | 770.3 | 777.2 | 858.6 | 2AS |
| 6567425 | CIM23 | FRANCOLIN#1/CHONTE//FRNCLN | 984.5 | 799.3 | 833.7 | 898.3 | 2AS |
| 6678574 | CIM24 | TAM200/PASTOR//TOBA97/3/FRNCLN/4/WHEAR//2*PRL/2*PASTOR | 682.3 | 696.8 | 759.2 | 698.8 | 2AS |
| 5398434 | CIM25 | FRANCOLIN#1 | 851.6 | 786.0 | 780.3 | 804.7 | 2AS |
| 6691523 | CIM26 | VEE/MJI//2*TUI/3/PASTOR/4/BERKUT/5/BAVIS | 439.9 | 551.5 | 641.7 | 459.3 | 2AS |
| 6566821 | CIM27 | DANPHE/PAURAQUE#1//MUNAL#1 | 952.7 | 899.4 | 828.0 | 914.5 | 2AS |
| 6681493 | CIM28 | BAJ#1*2/KISKADEE#1 | 994.2 | 909.0 | 849.4 | 944.1 | 2AS |
| 4747362 | CIM29 | SHATABDI | 1091.1 | 941.6 | 793.8 | 932.5 | 2AS |
| 6681464 | CIM3 | FRANCOLIN#1*2//ND643/2*WBLL1 | 941.7 | 847.7 | 821.2 | 898.3 | 2AS |
| 6001093 | CIM30 | SOKOLL/ROLF07 | 835.3 | 683.2 | 737.0 | 716.3 | 2AS |
| 6001439 | CIM31 | ATTILA/BAV92//PASTOR/3/ATTILA*2/PBW65 | 830.3 | 745.4 | 732.0 | 733.0 | 2AS |
| 6564531 | CIM32 | WHEAR/KUKUNA/3/C80.1/3*BATAVIA//2*WBLL1/4/T.DICOCCONPI94625/AE.SQUARROSA(372)//SHA4/CHIL/5/WHEAR/KUKUNA/3/C80.1/3*BATAVIA//2*WBLL1 | 775.3 | 626.8 | 669.5 | 651.4 | 2AS |
| 27001 | CIM33 | TEPOCAT89 | 1003.7 | 1099.3 | 818.0 | 983.9 | 2AS |
| 12725 | CIM34 | MILAN | 641.4 | 632.4 | 695.0 | 611.0 | 2NS |
| 7806808 | CIM35 | BORLAUG100F2014 | 994.4 | 838.0 | 766.0 | 870.7 | 2NS |
| 4905617 | CIM36 | ROELFSF2007 | 971.9 | 807.3 | 808.5 | 910.8 | 2AS |
| 6333158 | CIM37 | SUP152/BAJ#1 | 941.5 | 1023.8 | 783.8 | 911.5 | 2AS |
| 14337 | CIM38 | ATTILA | 977.6 | 985.2 | 807.9 | 966.4 | 2AS |
| 3855011 | CIM39 | VOROBEY | 587.3 | 859.1 | 734.8 | 686.6 | 2AS |
| 6692031 | CIM4 | CROC_1/AE.SQUARROSA(213)//PGO/10/ATTILA*2/9/KT/BAGE//FN/U/3/BZA/4/TRM/5/ALDAN/6/SERI/7/VEE#10/8/OPATA/11/ATTILA*2/PBW65 | 606.4 | 690.9 | 735.4 | 644.0 | 2AS |
| 1396784 | CIM40 | MILAN/MUNIA | 792.1 | 668.7 | 673.9 | 705.7 | 2NS |
| 5398434 | CIM41 | FRANCOLIN#1 | 905.4 | 804.5 | 792.5 | 833.2 | 2AS |
| 5390612 | CIM42 | SUPER152 | 883.2 | 957.3 | 813.5 | 923.6 | 2AS |
| 4755014 | CIM43 | KACHU#1 | 1330.0 | 1023.9 | 939.0 | 1156.3 | 2NS |
| 4754390 | CIM44 | MUTUS#1 | 690.8 | 644.1 | 706.2 | 659.5 | 2NS |
| 5794351 | CIM45 | AKEPA | 751.0 | 847.4 | 787.0 | 811.6 | 2AS |
| 6174889 | CIM46 | BOKOTA | 807.9 | 740.3 | 788.3 | 766.4 | 2NS |
| 5106304 | CIM47 | BAJ#1 | 947.8 | 968.8 | 811.8 | 927.1 | 2AS |
| 6177828 | CIM48 | FRNCLN/ROLF07 | 1014.1 | 906.5 | 864.1 | 948.7 | 2AS |
| 6175067 | CIM49 | NADI#2 | 1069.4 | 968.6 | 868.8 | 989.1 | 2NS |
| 6682922 | CIM5 | BAJ#1*2//ND643/2*WBLL1 | 1095.7 | 932.1 | 887.1 | 1008.6 | 2AS |
| 6337034 | CIM50 | FRNCLN*2/TECUE#1 | 870.6 | 718.4 | 799.4 | 789.7 | 2NS |
| 6341870 | CIM51 | MUCUY | 824.9 | 652.9 | 716.5 | 700.6 | 2NS |
| 6464471 | CIM52 | KACHU/BECARD//WBLL1*2/BRAMBLING | 1003.9 | 858.7 | 827.1 | 932.2 | 2AS |
| 6415882 | CIM53 | KIDEA | 867.8 | 780.0 | 751.6 | 772.1 | 2AS |
| 6681676 | CIM54 | QUAIU#1/SUP152 | 949.6 | 846.6 | 795.7 | 875.6 | 2AS |
| 6568165 | CIM55 | SUP152/FRNCLN | 805.7 | 746.3 | 741.3 | 740.5 | 2AS |
| 6682903 | CIM56 | FRNCLN/3/ND643//2*PRL/2*PASTOR/4/FRANCOLIN#1 | 510.0 | 511.8 | 661.1 | 477.5 | 2AS |
| 6684333 | CIM57 | SWSR22T.B./2*BLOUK#1//WBLL1*2/KURUKU | 560.8 | 600.3 | 682.7 | 559.0 | 2NS |
| 6679899 | CIM58 | FRANCOLIN#1*2//ND643/2*WBLL1 | 869.9 | 817.3 | 798.9 | 822.3 | 2AS |
| 6932283 | CIM59 | DANPHE/2*BAJ#1 | 831.5 | 821.5 | 773.1 | 771.3 | 2AS |
| 6679852 | CIM6 | PAURAQ/4/WHEAR/KUKUNA/3/C80.1/3*BATAVIA//2*WBLL1/5/PAURAQUE#1 | 1085.7 | 1005.5 | 808.4 | 989.6 | 2AS |
| 6932418 | CIM60 | BAJ#1*2/BECARD | 916.0 | 809.0 | 750.0 | 775.2 | 2AS |
| 6932429 | CIM61 | BAJ#1*2/5/SW89.5277/BORL95//SKAUZ/3/PRL/2*PASTOR/4/HEILO | 917.4 | 900.6 | 816.3 | 898.9 | 2AS |
| 6931516 | CIM62 | FRANCOLIN#1/BAJ#1 | 960.8 | 903.7 | 833.6 | 901.6 | 2AS |
| 6933207 | CIM63 | BAJ#1/CIRO16 | 1076.2 | 1019.0 | 858.7 | 1027.4 | 2NS |
| 6934570 | CIM64 | PBW343*2/KUKUNA//PBW343*2/KUKUNA/3/2*BAJ#1 | 994.1 | 917.1 | 794.4 | 882.9 | 2AS |
| 7175990 | CIM65 | BAJ#1/5/ATTILA/3*BCN//BAV92/3/TILHI/4/SHA7/VEE#5//ARIV92 | 1046.7 | 960.1 | 816.7 | 965.8 | 2NS |
| 7175993 | CIM66 | BAJ#1/CIRO16 | 966.2 | 877.4 | 821.2 | 883.3 | 2NS |
| 7176068 | CIM67 | SUP152/3/INQALAB91*2/TUKURU//WHEAR | 1039.7 | 915.5 | 871.7 | 964.9 | 2NS |
| 7176119 | CIM68 | SUP152/CIRO16 | 955.4 | 900.8 | 873.9 | 930.2 | 2NS |
| 7398595 | CIM69 | ATTILA*2/PBW65//KACHU/3/UP2338*2/KKTS*2//YANAC | 887.0 | 820.5 | 831.6 | 847.5 | 2NS |
| 6464471 | CIM7 | KACHU/BECARD//WBLL1*2/BRAMBLING | 1007.3 | 831.7 | 822.5 | 888.4 | 2AS |
| 7396039 | CIM70 | KASUKO | 1016.0 | 840.6 | 764.9 | 855.9 | 2NS |
| 7396072 | CIM71 | KFA/2*KACHU/3/ATTILA*2/PBW65//MURGA | 844.4 | 762.3 | 831.6 | 824.0 | 2NS |
| 7396125 | CIM72 | ATTILA*2/PBW65*2//KACHU/3/TRCH/HUIRIVIS#1 | 873.6 | 799.7 | 826.5 | 848.0 | 2NS |
| 7396133 | CIM73 | BORL14//KFA/2*KACHU | 1032.2 | 862.4 | 794.0 | 879.2 | 2NS |
| 7396135 | CIM74 | BORL14//KFA/2*KACHU | 977.8 | 815.5 | 794.3 | 892.1 | 2NS |
| 7396139 | CIM75 | BORL14//KFA/2*KACHU | 1101.2 | 744.0 | 750.7 | 855.2 | 2NS |
| 7396142 | CIM76 | BORL14//KFA/2*KACHU | 985.9 | 741.2 | 759.0 | 810.1 | 2NS |
| 7310905 | CIM77 | KACHU/KIRITATI//BORL14 | 775.2 | 765.1 | 769.5 | 743.4 | 2NS |
| 7400913 | CIM78 | KASUKO | 1152.0 | 889.7 | 815.6 | 956.7 | 2NS |
| 7400914 | CIM79 | KASUKO | 1100.3 | 901.5 | 821.6 | 953.2 | 2NS |
| 6679708 | CIM8 | MUTUS//KIRITATI/2*TRCH/3/WHEAR/KRONSTADF2004 | 1166.7 | 972.3 | 895.7 | 1024.0 | 2NS |
| 7400915 | CIM80 | KASUKO | 957.8 | 904.6 | 796.0 | 893.1 | 2NS |
| 7400916 | CIM81 | KASUKO | 956.6 | 819.8 | 745.8 | 835.2 | 2NS |
| 4836044 | CIM82 | ORL93320/ER2000 | 606.8 | 593.5 | 667.4 | 548.4 | 2NS |
| 4911209 | CIM83 | SW91.4903/3/URES/BOW//OPATA/4/SW94.15373 | 586.7 | 590.7 | 713.7 | 584.2 | 2AS |
| 4940678 | CIM84 | MON/TAN//ROMO96/3/METSO/4/FINSI | 535.4 | 627.7 | 608.3 | 545.6 | 2NS |
| 5090596 | CIM85 | MUU | 726.2 | 657.5 | 767.4 | 701.8 | 2AS |
| 5090672 | CIM86 | PFAU/WEAVER*2/3/WEAVER/ESDA//BORL95 | 734.2 | 801.3 | 797.3 | 776.8 | 2AS |
| 5106391 | CIM87 | SW8488*2/KURUKU | 874.2 | 918.2 | 718.8 | 839.0 | 2AS |
| 4936520 | CIM88 | FINSI/METSO | 462.3 | 461.4 | 574.0 | 461.8 | 2NS |
| 4936526 | CIM89 | FINSI/METSO | 474.8 | 496.9 | 574.0 | 472.2 | 2NS |
| 6569147 | CIM9 | BAJ#1*2/TINKIO#1 | 1122.7 | 959.1 | 888.0 | 1024.4 | 2AS |
| 4936533 | CIM90 | FINSI/METSO | 400.5 | 455.7 | 550.1 | 412.2 | 2NS |
| 4935134 | CIM91 | BAV92//IRENA/KAUZ/3/HUITES | 818.2 | 827.9 | 743.2 | 763.7 | 2AS |
| 4885822 | CIM92 | GONDO//BAU/MILAN/3/PASTOR | 592.1 | 662.2 | 724.9 | 632.7 | 2AS |
| 4756015 | CIM93 | ATTILA/3*BCN//BAV92/3/TILHI | 868.8 | 885.5 | 740.9 | 817.2 | 2NS |
| 4755014 | CIM94 | KACHU#1 | 1301.2 | 1029.5 | 920.5 | 1110.0 | 2NS |
| 4944857 | CIM95 | TEG/MIANYANG20//CHUM18/5*BCN | 823.1 | 749.9 | 687.7 | 704.7 | 2AS |
| 4937343 | CIM96 | JWS17/7/IAS58/4/KAL/BB//CJ71/3/ALD/5/CNR/6/THB/CEP7780/8/FINSI | 463.0 | 572.7 | 610.4 | 487.5 | 2NS |
| 4960851 | CIM97 | MILAN/ARA90//TNMU/TUI | 754.7 | 760.6 | 704.2 | 726.5 | 2NS |
| 7025946 | IND1 | HD2967 | 781.2 | 716.0 | 771.1 | 762.3 | 2NS |
| 6927036 | IND10 | WH1105 | 694.2 | 608.5 | 705.3 | 667.4 | 2NS |
| 7895088 | IND11 | WH1124 | 927.4 | 813.2 | 829.9 | 840.3 | 2AS |
| 7895089 | IND12 | WH1142 | 916.6 | 896.7 | 798.4 | 891.8 | 2AS |
| 7895090 | IND13 | HD3171 | 770.4 | 765.6 | 750.2 | 722.9 | 2NS |
| 7895091 | IND14 | K1317 | 395.2 | 554.0 | 653.9 | 450.7 | 2AS |
| 3575624 | IND15 | C306 | 1087.4 | 917.6 | 732.8 | 905.4 | 2AS |
| 7025950 | IND16 | DBW39 | 965.2 | 934.9 | 771.3 | 900.2 | 2NS |
| 4048863 | IND17 | HD2733 | 866.2 | 962.6 | 718.9 | 811.3 | 2AS |
| 7025907 | IND18 | HD2888 | 1143.3 | 945.2 | 741.1 | 936.6 | 2AS |
| 152830 | IND19 | MAGHAR | 978.2 | 796.9 | 764.8 | 815.2 | 2AS |
| 7895083 | IND2 | DBW90 | 855.4 | 868.2 | 828.1 | 879.0 | 2AS |
| 7025921 | IND20 | K0307 | 974.1 | 891.4 | 793.7 | 888.4 | 2AS |
| 7522443 | IND21 | K1006 | 945.1 | 887.3 | 789.9 | 853.4 | 2AS |
| 7895097 | IND28 | HI1605 | 646.5 | 597.2 | 675.3 | 580.3 | 2NS |
| 7895084 | IND3 | HD3043 | 709.5 | 649.3 | 644.5 | 622.1 | 2NS |
| 7895098 | IND30 | DBW93 | 737.2 | 723.1 | 746.7 | 693.5 | 2AS |
| 7025931 | IND31 | HD2932 | 1410.4 | 1093.4 | 824.8 | 1144.5 | 2AS |
| 7025955 | IND32 | MACS6222 | 981.5 | 1043.9 | 839.9 | 981.3 | 2AS |
| 7895099 | IND33 | MACS6478 | 1252.8 | 1051.3 | 816.1 | 1080.4 | 2NS |
| 23028 | IND34 | NI5439 | 1178.3 | 1132.6 | 861.2 | 1112.9 | 2AS |
| 7895100 | IND35 | NIAW1415 | 955.2 | 860.8 | 749.3 | 838.6 | 2AS |
| 7895101 | IND36 | NIAW34 | 921.2 | 880.9 | 854.2 | 900.2 | 2AS |
| 7025915 | IND38 | RAJ4083 | 1214.0 | 1003.7 | 838.3 | 1046.6 | 2AS |
| 7895085 | IND4 | DBW88 | 1284.4 | 1024.9 | 934.2 | 1105.5 | 2NS |
| 6927038 | IND5 | HD3059 | 1315.9 | 1020.5 | 936.2 | 1135.9 | 2NS |
| 7895086 | IND6 | HD3086 | 826.2 | 901.8 | 841.6 | 915.6 | 2AS |
| 7895087 | IND7 | PBW644 | 1008.3 | 794.0 | 796.9 | 875.3 | 2AS |
| 7025929 | IND8 | WH1021 | 1043.5 | 1109.2 | 787.4 | 988.4 | 2AS |
| 7025960 | IND9 | WH1080 | 690.0 | 810.3 | 802.0 | 802.0 | 2AS |
| 264591 | NPL1 | BHRIKUTI | 1088.6 | 1098.6 | 923.5 | 1068.2 | 2AS |
| 7895108 | NPL10 | BL4407 | 1116.2 | 923.6 | 906.8 | 1011.8 | 2AS |
| 7895109 | NPL11 | BL4725 | 867.5 | 902.2 | 761.0 | 811.5 | 2AS |
| 7895110 | NPL12 | BL4707 | 985.7 | 864.1 | 849.6 | 908.1 | 2AS |
| 7895111 | NPL13 | BL4406 | 1111.4 | 941.9 | 910.2 | 999.0 | 2AS |
| 7895112 | NPL14 | BL4682 | 979.8 | 823.9 | 774.8 | 858.7 | 2AS |
| 7895113 | NPL15 | BL4621 | 1141.9 | 1176.0 | 938.9 | 1130.2 | 2AS |
| 7895114 | NPL16 | BL4622 | 1056.2 | 1144.1 | 901.7 | 1069.8 | 2AS |
| 7895115 | NPL17 | NL1164 | 887.8 | 937.0 | 793.6 | 876.8 | 2NS |
| 7895116 | NPL18 | NL1202 | 1112.1 | 999.2 | 894.6 | 1050.5 | 2NS |
| 7895117 | NPL19 | NL1249 | 731.4 | 736.5 | 787.4 | 730.0 | 2AS |
| 5956817 | NPL2 | GAUTAM | 996.2 | 847.8 | 808.9 | 878.1 | 2AS |
| 7895118 | NPL20 | NL1270 | 550.9 | 746.2 | 730.9 | 602.7 | 2NS |
| 7895119 | NPL21 | NL1178 | 658.6 | 772.7 | 722.4 | 703.1 | 2AS |
| 7895120 | NPL22 | NL1226 | 512.6 | 621.8 | 712.3 | 598.1 | 2AS |
| 7895121 | NPL23 | NL1231 | 1012.1 | 827.7 | 769.7 | 894.0 | 2AS |
| 7895122 | NPL24 | WK2432 | 792.9 | 946.1 | 791.7 | 848.8 | 2AS |
| 7895123 | NPL25 | WK2375 | 895.7 | 776.6 | 776.0 | 800.1 | 2AS |
| 7895124 | NPL26 | WK2123 | 1158.9 | 986.8 | 888.7 | 1074.8 | 2AS |
| 7895125 | NPL27 | WK2286 | 1020.9 | 712.2 | 742.3 | 790.7 | 2AS |
| 7895126 | NPL28 | WK2395 | 771.2 | 708.5 | 701.2 | 664.9 | 2AS |
| 7895104 | NPL3 | WK1204 | 1063.8 | 775.9 | 789.8 | 876.1 | 2AS |
| 6561770 | NPL4 | ADITYA | 1233.0 | 795.5 | 838.4 | 986.4 | 2AS |
| 5218199 | NPL5 | VIJAY | 922.3 | 929.5 | 923.2 | 959.7 | 2AS |
| 7895105 | NPL6 | DHAWALAGIRI | 1270.8 | 1007.9 | 819.3 | 1052.3 | 2AS |
| 6308951 | NPL7 | BL3623 | 1145.4 | 948.3 | 826.1 | 993.0 | 2AS |
| 7895106 | NPL8 | BL3629 | 1199.9 | 1027.0 | 848.9 | 1053.6 | 2AS |
| 7895107 | NPL9 | BL4341 | 1185.3 | 904.6 | 946.0 | 1060.6 | 2AS |

**Table S2:** Statistical and genetic parameters for Days to heading and plant height over the respective year and pool over years

| **Statistical parameters** | **DH 2019** | **DH 2020** | **DH 2021** | **DH_POOL** | **PH 2019** | **PH 2020** | **PH 2021** | **PH_POOL** |
| --- | --- | --- | --- | --- | --- | --- | --- | --- |
| Heritability | 0.95 | 0.91 | 0.97 | 0.86 | 0.95 | 0.97 | 0.92 | 0.74 |
| Genotype Variance | 14.20 | 11.70 | 17.90 | 10.44 | 75.26 | 52.53 | 52.04 | 30.85 |
| Genotype × Year Variance |  |  |  | 4.15 |  |  |  | 29.09 |
| Year variance |  |  |  | 5.69 |  |  |  | 46.28 |
| Residual variance | 1.53 | 2.37 | 1.09 | 1.66 | 8.50 | 3.73 | 9.52 | 7.24 |
| Grand Mean | 67.53 | 64.44 | 69.15 | 67.04 | 94.75 | 103.09 | 108.27 | 102.04 |
| LSD | 2.38 | 2.90 | 2.03 | 3.39 | 5.60 | 3.75 | 5.83 | 7.96 |
| CV | 1.83 | 2.39 | 1.51 | 1.92 | 3.08 | 1.87 | 2.85 | 2.64 |
| Genotype significance | 0 | 0 | 0 | 1.88E-54 | 0 | 0 | 0 | 3.9204E-26 |
| Genotype × Year significance |  |  |  | 2.84E-73 |  |  |  | 8.30E-107 |
| Year significance |  |  |  | 0.000686 |  |  |  | 1.27592E-05 |

**Table S3:** The unique quantitative trait nucleotides (QTNs) controlling the Septoria tritici blotch resistance

| **QTNs** | **SNP** | **Chr** | **Pos** | **Effect** | **LOG P VALUE** | **Percent Variation** | **Year** | **Gene ID** | **Position** | **Description with Prot ID** | **Biological function** |
| --- | --- | --- | --- | --- | --- | --- | --- | --- | --- | --- | --- |
| Q.CIM.stb.1AL.6 | GENE-0115_375 | 1A | 5.75E+08 | 81.30624 | 3.710828 |  | BLINKSTB2020 | TraesCS1A02G416500 | chr1A:574826362-574832131(-) | NA |  |
| Q.CIM.stb.1AL.7 | Kukri_c30490_328 | 1A | 5.75E+08 | -81.1384 | 3.785564 |  | BLINKSTB2020 | TraesCS1A02G416600 | chr1A:574933843-574935896(+) | Transcription elongation factor 1 homolog [UniProtKB/Swiss-Prot:Q8LHP0] | transcription elongation from RNA polymerase II promoter |
| Q.CIM.stb.1AL.8 | RAC875_c55934_165 | 1A | 5.75E+08 | -78.5204 | 3.505254 |  | BLINKSTB2020 | TraesCS1A02G416600 | chr1A:574933843-574935896(+) | Transcription elongation factor 1 homolog [UniProtKB/Swiss-Prot:Q8LHP0] | transcription elongation from RNA polymerase II promoter |
| Q.CIM.stb.1AS.2 | Tdurum_contig9199_714 | 1A | 45796934 | -55.9255 | 5.963495 | 1.354226 | FarmCPUSTB2019 | TraesCS1A02G064100 | chr1A:45793606-45797826(+) | Protein IQ-DOMAIN 1 [UniProtKB/Swiss-Prot:Q9SF32] |  |
| Q.CIM.stb.1BL.3 | Kukri_c30461_857 | 1B | 6.15E+08 | 23.78835 | 5.660014 | 1.322307 | FarmCPUSTB2021 | TraesCS1B02G382600 | chr1B:614788759-614794654(+) | Phospholipase D beta 1 [UniProtKB/Swiss-Prot:P93733] | phosphatidylcholine metabolic process |
|  |  |  |  |  |  |  |  | TraesCS1B02G379700 | chr1B:612893037-612894939(+) | Probable cytokinin riboside 5'-monophosphate phosphoribohydrolase LOGL7 [UniProtKB/Swiss-Prot:Q5TKP8] | cytokinin biosynthetic process [GO:0009691] |
|  |  |  |  |  |  |  |  | TraesCS1B02G383300 | chr1B:615117570-615125086(+) | Protein ANTHESIS POMOTING FACTOR 1 [UniProtKB/Swiss-Prot:Q9LYK6] | histone H3-K4 trimethylation [GO:0080182] |
|  |  |  |  |  |  |  |  | TraesCS1B02G380000 | chr1B:613020322-613022195(+) | Transcription factor LATE FLOWERING [UniProtKB/Swiss-Prot:A0A0P0WQ90] | regulation of long-day photoperiodism, flowering [GO:0048586] |
| Q.CIM.stb.1BS.2 | Ra_c4899_609 | 1B | 2.34E+08 | 79.80508 | 3.145206 |  | MLMSTB2020 PC6 | TraesCS1B02G150900 | chr1B:234251618-234254193(-) | Ras-related protein RGP2 [UniProtKB/Swiss-Prot:Q40723] |  |
| Q.CIM.stb.1DS.1 | D_contig13475_402 | 1D | 20045042 | 88.93567 | 3.153394 |  | MLMSTB2020 PC6 | TraesCS5A02G396500 | chr5A:591313846-591320402(+) | NA |  |
| Q.CIM.stb.2AL.4 | RAC875_c19328_236 | 2A | 4.01E+08 | -99.8754 | 3.421572 |  | MLMSTB2019 PC6 | NA | #N/A | #N/A |  |
| Q.CIM.stb.2AL.6 | wsnp_CAP7_c1592_791887 | 2A | 7.31E+08 | 128 | 7.091825 | 7.559225 | BLINKSTB2021 | TraesCS2A02G502400 | chr2A:731156504-731163306(-) | Plasma membrane ATPase [UniProtKB/Swiss-Prot:P83970] | ion transmembrane transport, regulation of intracellular pH |
|  |  |  |  |  |  |  |  | TraesCS2A02G501900 | chr2A:729852393-729858636(+) | Farnesyl pyrophosphate synthase [UniProtKB/Swiss-Prot:Q4JHN6] | farnesyl diphosphate biosynthetic process [GO:0045337] |
|  |  |  |  |  |  |  |  | TraesCS2A02G501000 | chr2A:729576096-729579455(+) | Ribosomal RNA processing protein 1 homolog B [UniProtKB/Swiss-Prot:Q14684] | negative regulation of GTPase activity [GO:0034260] |
|  |  |  |  |  |  |  |  | TraesCS2A02G501300 | chr2A:729687927-729689703(+) | GTPase activating protein 1 [UniProtKB/Swiss-Prot:Q6YWF1] | positive regulation of defense response to bacterium, incompatible interaction [GO:1902479] |
|  |  |  |  |  |  |  |  | TraesCS2A02G502400 | chr2A:731156504-731163306(-) | Plasma membrane ATPase [UniProtKB/Swiss-Prot:P83970] | proton export across plasma membrane [GO:0120029] |
| Q.CIM.stb.2AL.7 | BS00082084_51 | 2A | 7.33E+08 | 93.23496 | 7.806079 | 9.861082 | FarmCPUSTB2020 | TraesCS2A02G503000 | chr2A:732569049-732570858(-) | NA | defense response, negative regulation of translation |
|  |  |  |  |  |  |  |  | TraesCS2A02G504400 | chr2A:733287115-733289358(-) | Subtilisin-like protease 4 [UniProtKB/Swiss-Prot:A9QY38] | arbuscular mycorrhizal association [GO:0036377] |
|  |  |  |  |  |  |  |  | TraesCS2A02G505000 | chr2A:733560806-733563422(+) | Sucrose transport protein SUT5 [UniProtKB/Swiss-Prot:A2X6E6] | #N/A |
|  |  |  |  |  |  |  |  | TraesCS2A02G506200 | chr2A:734148608-734153261(-) | Vacuolar cation/proton exchanger 3 [UniProtKB/Swiss-Prot:Q6K1C4] | cellular calcium ion homeostasis [GO:0006874] |
|  |  |  |  |  |  |  |  | TraesCS2A02G504600 | chr2A:733394610-733398877(-) | COP9 signalosome complex subunit 5 [UniProtKB/Swiss-Prot:Q8H936] | #N/A |
|  |  |  |  |  |  |  |  | TraesCS2A02G503100 | chr2A:732580211-732581104(+) | Ethylene-responsive transcription factor ERF003 [UniProtKB/Swiss-Prot:Q94AW5] | ethylene-activated signaling pathway [GO:0009873] |
|  |  |  |  |  |  |  |  | TraesCS2A02G505800 | chr2A:733921860-733929336(-) | NADPH--cytochrome P450 reductase [UniProtKB/Swiss-Prot:Q05001] | #N/A |
| Q.CIM.stb.2AS.1 | RAC875_c21868_792 | 2A | 30452856 | -42.695 | 5.211511 | 0.388925 | FarmCPUSTB2020 | TraesCS2A02G069400 | chr2A:30451134-30464943(-) | Acetyl-CoA carboxylase 2 [UniProtKB/Swiss-Prot:B9FK36] |  |
|  |  |  |  |  |  |  |  | TraesCS2A02G069900 | chr2A:30764411-30766386(+) | 2-alkenal reductase (NADP(+)-dependent) [UniProtKB/Swiss-Prot:Q9SLN8] | #N/A |
|  |  |  |  |  |  |  |  | TraesCS2A02G069400 | chr2A:30451134-30464943(-) | Acetyl-CoA carboxylase 2 [UniProtKB/Swiss-Prot:B9FK36] | malonyl-CoA biosynthetic process [GO:2001295] |
|  |  |  |  |  |  |  |  | TraesCS2A02G071000 | chr2A:31648562-31686564(-) | Arginine decarboxylase [UniProtKB/Swiss-Prot:P22220] | arginine catabolic process [GO:0006527] |
|  |  |  |  |  |  |  |  | TraesCS2A02G066400 | chr2A:29933733-29935186(+) | Probable cinnamyl alcohol dehydrogenase 6 [UniProtKB/Swiss-Prot:Q7XWU3] | lignin biosynthetic process [GO:0009809] |
|  |  |  |  |  |  |  |  | TraesCS2A02G072100 | chr2A:32026318-32028546(-) | Two-component response regulator ORR42 [UniProtKB/Swiss-Prot:Q7XN30] | cytokinin-activated signaling pathway [GO:0009736] |
|  |  |  |  |  |  |  |  | TraesCS2A02G068400 | chr2A:30197044-30199142(-) | LRR receptor-like serine/threonine-protein kinase FLS2 [UniProtKB/Swiss-Prot:Q9FL28] | detection of bacterium [GO:0016045] |
|  |  |  |  |  |  |  |  | TraesCS2A02G069100 | chr2A:30305253-30308602(-) | LRR receptor-like serine/threonine-protein kinase EFR [UniProtKB/Swiss-Prot:C0LGT6] | innate immune response-activating signal transduction [GO:0002758] |
|  |  |  |  |  |  |  |  | TraesCS2A02G066700 | chr2A:30020633-30021500(-) | Ribulose bisphosphate carboxylase small chain PWS4.3, chloroplastic [UniProtKB/Swiss-Prot:P00871] | photorespiration [GO:0009853] |
| Q.CIM.stb.2AS.2 | AX-94639471 | 2A | 52129997 | -160.887 | 3.589311 |  | BLINKSTB ACROSS YEARS | TraesCS2A02G099200 | chr2A:52126850-52131904(+) | NA |  |
| Q.CIM.stb.2BL.4 | BS00047070_51 | 2B | 7.15E+08 | 464 | 3.936493 |  | BLINKSTB2020 | TraesCS2B02G520100 | chr2B:714779662-714782762(-) | U11/U12 small nuclear ribonucleoprotein 25 kDa protein [UniProtKB/Swiss-Prot:Q84WS8] | mRNA splicing, via spliceosome |
| Q.CIM.stb.2BL.5 | BS00065327_51 | 2B | 7.31E+08 | 41.74597 | 3.201556 |  | MLMSTB2021 PC6 | TraesCS2B02G535600 | chr2B:731480831-731482520(-) | 3-ketoacyl-CoA synthase 6 [UniProtKB/Swiss-Prot:Q9XF43] | fatty acid biosynthetic process |
| Q.CIM.stb.2BL.7 | RAC875_c50422_299 | 2B | 7.34E+08 | 39.25921 | 3.074288 |  | MLMSTB2021 PC6 | TraesCS2B02G538100 | chr2B:733642201-733647003(-) | Probable inactive receptor kinase RLK902 [UniProtKB/Swiss-Prot:Q9LVI6] | protein phosphorylation and cell surface receptor signaling pathway |
| Q.CIM.stb.2BL.8 | AX-94945263 | 2B | 7.49E+08 | -40.2838 | 3.085764 |  | MLMSTB2021 PC6 | TraesCS2B02G553700 | chr2B:749043905-749044536(-) | NA |  |
| Q.CIM.stb.2BS.1 | BS00010055_51 | 2B | 72610501 | 57.93331 | 4.087254 |  | FarmCPUSTB2019 | TraesCS2B02G110800 | chr2B:72608545-72619945(-) | Lon protease homolog, mitochondrial [UniProtKB/Swiss-Prot:Q69UZ3] | cellular response to oxidative stress |
| Q.CIM.stb.2BS.2 | BobWhite_c892_73 | 2B | 2.12E+08 | -5120 | 3.75108 |  | BLINKSTB2021 | TraesCS2B02G221600 | chr2B:211525650-211535423(+) | Importin-5 [UniProtKB/Swiss-Prot:Q8BKC5] | intracellular protein transport |
| Q.CIM.stb.2DS.1 | GENE-0581_156 | 2D | 49881735 | 384 | 4.831081 | 8.595425 | BLINKSTB ACROSS YEARS | TraesCS2D02G097700 | chr2D:49879249-49884545(-) | CBL-interacting protein kinase 31 [UniProtKB/Swiss-Prot:Q6X4A2] | protein phosphorylation;intracellular signal transduction |
| Q.CIM.stb.3AL.7 | RFL_Contig2394_439 | 3A | 7.49E+08 | 49.40193 | 4.160511 |  | FarmCPUSTB2020 | TraesCS3A02G537600 | chr3A:749276696-749281113(+) | DnaJ protein P58IPK homolog B [UniProtKB/Swiss-Prot:Q5JNB5] |  |
| Q.CIM.stb.3AS.1 | RAC875_c45016_79 | 3A | 53837058 | -24.9983 | 4.07456 |  | FarmCPUSTB2021 | TraesCS3A02G083800 | chr3A:53838186-53842020(-) | NA |  |
| Q.CIM.stb.3BL.4 | wsnp_RFL_Contig3396_3515504 | 3B | 5.41E+08 | -104.6 | 3.073965 |  | MLMSTB2020 PC6 | TraesCS3B02G335000 | chr3B:541080846-541085261(-) | Clp protease adapter protein ClpF, chloroplastic [UniProtKB/Swiss-Prot:Q67Y99] | chloroplastic endopeptidase Clp complex |
| Q.CIM.stb.3BL.5 | wsnp_CAP12_c2297_1121142 | 3B | 7.83E+08 | 33.7796 | 4.287921 |  | FarmCPUSTB2020 | TraesCS3B02G547900 | chr3B:783465998-783469436(-) | Uridine 5'-monophosphate synthase [UniProtKB/Swiss-Prot:Q9LDN2] | UMP biosynthetic process, pyrimidine nucleotide biosynthetic process, metabolic process |
| Q.CIM.stb.3BL.6 | AX-94437380 | 3B | 8.15E+08 | -54.3757 | 5.087521 | 0.338167 | FarmCPUSTB2020 | TraesCS3B02G589300 | chr3B:814795775-814800995(-) | Disease resistance protein Piks-2 [UniProtKB/Swiss-Prot:P0DO10] |  |
| Q.CIM.stb.3BS.1 | Kukri_c88294_60 | 3B | 3904458 | 82.60869 | 3.026654 |  | MLMSTB PC6 ACROSS YEARS | TraesCS3B02G007210 | NA | NA |  |
| Q.CIM.stb.3BS.2 | AX-94394857 | 3B | 39910845 | -49.0643 | 3.984068 |  | BLINKSTB ACROSS YEARS | TraesCS3B02G067400 | chr3B:39905297-39910826(-) | NA |  |
| Q.CIM.stb.3BS.3 | CAP8_c8651_206 | 3B | 40246061 | 41.189 | 3.650904 |  | BLINKSTB ACROSS YEARS | TraesCS3B02G068000 | chr3B:40243799-40247161(-) | 14-3-3-like protein B [UniProtKB/Swiss-Prot:Q43470] |  |
| Q.CIM.stb.4AL.1 | wsnp_Ku_c1205_2398925 | 4A | 5.98E+08 | -61.0043 | 3.73299 |  | BLINKSTB2019 | TraesCS4A02G299700 | chr4A:597905176-597910407(+) | NA |  |
| Q.CIM.stb.4AL.2 | BobWhite_c20909_243 | 4A | 5.99E+08 | -66.7936 | 3.621641 |  | BLINKSTB2019 | TraesCS4A02G301600 | chr4A:598590265-598592384(+) | 2-methyl-6-phytyl-1,4-hydroquinone methyltransferase 2, chloroplastic [UniProtKB/Swiss-Prot:Q2QM69] |  |
| Q.CIM.stb.4AL.3 | AX-94467468 | 4A | 5.99E+08 | 70.86701 | 3.543723 |  | BLINKSTB2019 | TraesCS4A02G303400 | chr4A:599321764-599326140(+) | Uncharacterized oxidoreductase At1g06690, chloroplastic [UniProtKB/Swiss-Prot:Q94A68] |  |
| *Q.CIM.stb.4AL.4* | BS00092859_51 | 4A | 5.99E+08 | 0.21 | -70.87 | 3.54 | BLINKSTB2019 | NA |  |  |  |
| Q.CIM.stb.4AL.5 | RAC875_c78248_154 | 4A | 6.02E+08 | -76.087 | 3.059717 |  | MLMSTB2020 PC6 | TraesCS4A02G308800 | chr4A:602269460-602272127(-) | Cytochrome P450 72A14 [UniProtKB/Swiss-Prot:Q9LUC6] |  |
| Q.CIM.stb.4AL.6 | BS00021957_51 | 4A | 6.93E+08 | 34.57209 | 6.215447 | 2.03706 | FarmCPUSTB2021 | TraesCS4A02G422900 | chr4A:693274378-693278701(+) | Probable aminotransferase ACS10 [UniProtKB/Swiss-Prot:Q9LQ10] | 1-aminocyclopropane-1-carboxylate biosynthetic process |
|  |  |  |  |  |  |  |  | TraesCS4A02G422400 | chr4A:692545985-692551526(+) | Spastin [UniProtKB/Swiss-Prot:Q6AZT2] | axonal transport of mitochondrion [GO:0019896] |
|  |  |  |  |  |  |  |  | TraesCS4A02G422100 | chr4A:692382875-692386116(-) | 26S proteasome non-ATPase regulatory subunit 8 homolog A [UniProtKB/Swiss-Prot:Q9SGW3] | regulation of anthocyanin biosynthetic process [GO:0031540] |
|  |  |  |  |  |  |  |  | TraesCS4A02G423900 | chr4A:693783398-693785810(+) | Subtilisin-like protease 4 [UniProtKB/Swiss-Prot:A9QY38] | response to symbiotic bacterium [GO:0009609] |
|  |  |  |  |  |  |  |  | TraesCS4A02G422900 | chr4A:693274378-693278701(+) | Probable aminotransferase ACS10 [UniProtKB/Swiss-Prot:Q9LQ10] | #N/A |
| Q.CIM.stb.4AL.7 | TA001746-1415 | 4A | 7.34E+08 | 51.71376 | 3.767748 |  | FarmCPUSTB2019 | TraesCS4A02G474200 | chr4A:733705236-733711789(+) | NA |  |
| Q.CIM.stb.4BL.2 | IACX378 | 4B | 5.69E+08 | 960 | 3.727603 |  | BLINKSTB2021 | TraesCS4B02G285000 | chr4B:568555627-568559531(+) | Ribulose-phosphate 3-epimerase, chloroplastic [UniProtKB/Swiss-Prot:Q9ZTP5] | cellular carbohydrate metabolic process |
| Q.CIM.stb.4BL.3 | RAC875_c107130_384 | 4B | 6.49E+08 | -66.6332 | 3.733272 |  | BLINKSTB2019 | TraesCS4B02G358800 | chr4B:649469807-649475248(+) | Respiratory burst oxidase homolog protein B [UniProtKB/Swiss-Prot:Q5ZAJ0] |  |
| Q.CIM.stb.4BL.4 | Kukri_c18722_56 | 4B | 6.5E+08 | 69.42575 | 3.727149 |  | BLINKSTB2019 | TraesCS4B02G359300 | chr4B:649602163-649607404(-) | WD repeat-containing protein 44 [UniProtKB/Swiss-Prot:Q9XSC3] |  |
| Q.CIM.stb.4BL.5 | RAC875_c19259_1902 | 4B | 6.57E+08 | 1024 | 5.43806 | 1.329759 | BLINKSTB ACROSS YEARS | TraesCS4B02G371400 | chr4B:656903504-656911031(-) | Protein ARABIDILLO 1 [UniProtKB/Swiss-Prot:O22161] |  |
|  |  |  |  |  |  |  |  | TraesCS4B02G369200 | chr4B:654892610-654894282(+) | Anthranilate N-benzoyltransferase protein 1 [UniProtKB/Swiss-Prot:O24645] | #N/A |
|  |  |  |  |  |  |  |  | TraesCS4B02G375100 | chr4B:659154074-659173043(-) | CCR4-NOT transcription complex subunit 1 [UniProtKB/Swiss-Prot:Q6ZQ08] | gene silencing by miRNA [GO:0035195] |
|  |  |  |  |  |  |  |  | TraesCS4B02G371500 | chr4B:657159331-657160442(+) | BTB/POZ and MATH domain-containing protein 4 [UniProtKB/Swiss-Prot:Q9SRV1] | cellular response to salt stress [GO:0071472] |
|  |  |  |  |  |  |  |  | TraesCS4B02G372100 | chr4B:657243113-657246017(+) | Probable leucine-rich repeat receptor-like protein kinase At1g35710 [UniProtKB/Swiss-Prot:Q9LP24] | hormone-mediated signaling pathway [GO:0009755] |
|  |  |  |  |  |  |  |  | TraesCS4B02G374000 | chr4B:658166283-658167487(+) | DNA-directed RNA polymerases II, IV and V subunit 9A [UniProtKB/Swiss-Prot:Q6NLH0] | maintenance of transcriptional fidelity during DNA-templated transcription elongation from RNA polymerase II promoter [GO:0001193] |
|  |  |  |  |  |  |  |  | TraesCS4B02G371200 | chr4B:656891495-656893325(+) | UDP-glycosyltransferase 73C1 [UniProtKB/Swiss-Prot:Q9ZQ99] | #N/A |
| Q.CIM.stb.4BS.1 | AX-95148759 | 4B | 59232947 | -32.029 | 4.473369 |  | FarmCPU ACROSS YEARS | TraesCS4B02G066400 | chr4B:59231238-59234415(+) | Formate dehydrogenase, mitochondrial [UniProtKB/Swiss-Prot:Q9ZRI8] |  |
| Q.CIM.stb.4DL.1 | BS00103682_51 | 4D | 4.55E+08 | 33.18977 | 7.647112 | 1.340224 | FarmCPUSTB2021 | TraesCS4D02G283600 | chr4D:455341941-455346185(-) | Aluminum-activated malate transporter 1 [UniProtKB/Swiss-Prot:Q76LB1] | ion transmembrane transport |
| Q.CIM.stb.5AL.10 | BS00022071_51 | 5A | 5.87E+08 | -95.5077 | 3.349168 |  | MLMSTB2020 PC6 | NA | #N/A | #N/A |  |
| Q.CIM.stb.5AL.11 | wsnp_AJ612027A_Ta_2_1 | 5A | 5.89E+08 | 95.50774 | 3.349168 |  | MLMSTB2020 PC6 | TraesCS5A02G392300 | chr5A:588548725-588552987(+) | Solute carrier family 25 member 44 [UniProtKB/Swiss-Prot:Q8BGF9] | transmembrane transport |
| Q.CIM.stb.5AL.12 | IAAV8629 | 5A | 5.91E+08 | 36.84204 | 5.442344 | 4.896963 | FarmCPUSTB2021 | TraesCS5A02G396500 | chr5A:591313846-591320402(+) | NA |  |
| Q.CIM.stb.5AL.14 | BS00049403_51 | 5A | 6.07E+08 | -48.597 | 5.015898 | 1.699275 | FarmCPU ACROSS YEARS | TraesCS5A02G419900 | chr5A:606810994-606811710(+) | NA |  |
| Q.CIM.stb.5AL.2 | BS00110075_51 | 5A | 5.5E+08 | -39.9263 | 4.27292 |  | FarmCPU ACROSS YEARS | TraesCS5A02G347300 | chr5A:549797658-549800976(+) | Subtilisin-like protease SBT3.8 [UniProtKB/Swiss-Prot:Q9SZY3] | proteolysis |
| Q.CIM.stb.5AL.3 | BS00065551_51 | 5A | 5.57E+08 | 544 | 6.3446 | 5.270172 | BLINKSTB2020 | TraesCS5A02G353500 | chr5A:556529046-556531643(+) | NAC domain-containing protein 86 [UniProtKB/Swiss-Prot:Q9FFI5] | regulation of transcription, DNA-templated |
|  |  |  |  |  |  |  |  | TraesCS5A02G355000 | chr5A:557126728-557129781(-) | Protein PLASTID MOVEMENT IMPAIRED 1 [UniProtKB/Swiss-Prot:Q9C8E6] | abscisic acid homeostasis [GO:1902265] |
|  |  |  |  |  |  |  |  | TraesCS5A02G354500 | chr5A:557076709-557077548(+) | RING-H2 finger protein ATL80 [UniProtKB/Swiss-Prot:Q9LM69] | cellular response to cold [GO:0070417] |
|  |  |  |  |  |  |  |  | TraesCS5A02G355900 | chr5A:558304063-558305554(-) | Cytosolic sulfotransferase 5 [UniProtKB/Swiss-Prot:Q9M1V2] | flavonoid metabolic process [GO:0009812] |
|  |  |  |  |  |  |  |  | TraesCS5A02G356000 | chr5A:558321388-558331318(-) | Actin-related protein 4 [UniProtKB/Swiss-Prot:Q6ZJW9] | histone H4 acetylation [GO:0043967] |
|  |  |  |  |  |  |  |  | TraesCS5A02G355300 | chr5A:557319488-557323790(-) | Nicotinate-nucleotide pyrophosphorylase [carboxylating], chloroplastic [UniProtKB/Swiss-Prot:Q0IZS0] | NAD biosynthetic process [GO:0009435] |
|  |  |  |  |  |  |  |  | TraesCS5A02G353700 | chr5A:556675929-556684951(+) | UTP--glucose-1-phosphate uridylyltransferase [UniProtKB/Swiss-Prot:Q43772] | UDP-glucose metabolic process [GO:0006011] |
| Q.CIM.stb.5AL.4 | Tdurum_contig10210_425 | 5A | 5.7E+08 | -97.8193 | 12.21014 | 3.441174 | FarmCPU ACROSS YEARS | TraesCS5A02G370500 | chr5A:569863642-569869224(+) | Rhomboid-like protein 15 [UniProtKB/Swiss-Prot:Q8LB17] |  |
| Q.CIM.stb.5AL.6 | RAC875_c23775_406 | 5A | 5.72E+08 | 93.08433 | 3.640867 |  | MLMSTB2019PC6 | NA | #N/A | #N/A |  |
| Q.CIM.stb.5AL.7 | AX-94432465 | 5A | 5.73E+08 | -90.0279 | 3.406062 |  | MLMSTB2019 PC6 | TraesCS5A02G375000 | chr5A:573039922-573045194(+) | Ornithine aminotransferase, mitochondrial [UniProtKB/Swiss-Prot:Q10G56] | response to oxidative stress, response to water deprivation, defense response to bacterium, arginine catabolic process to glutamate, L-proline biosynthetic process, |
| Q.CIM.stb.5AL.8 | Tdurum_contig44343_1039 | 5A | 5.74E+08 | -89.1542 | 3.349322 |  | MLMSTB2019 PC6 | TraesCS5A02G375500 | chr5A:573586786-573591315(+) | BTB/POZ domain-containing protein At1g30440 [UniProtKB/Swiss-Prot:Q9S9Q9] | transcriptional regulation, cytoskeleton dynamics, ion channel assembly and gating, and targeting proteins for ubiquitination |
| Q.CIM.stb.5AL.9 | AX-94429192 | 5A | 5.81E+08 | 1664 | 7.678301 | 3.646174 | BLINKSTB2019 | TraesCS5A02G382700 | chr5A:580794356-580798659(+) | Probable histone H2A variant 3 [UniProtKB/Swiss-Prot:Q84MP7] |  |
|  |  |  |  |  |  |  |  | TraesCS5A02G381300 | chr5A:579242640-579243506(+) | BTB/POZ and MATH domain-containing protein 1 [UniProtKB/Swiss-Prot:Q8L765] | cellular response to salt stress [GO:0071472] |
|  |  |  |  |  |  |  |  | TraesCS5A02G381600 | chr5A:579409334-579410364(-) | Two-component response regulator ORR41 [UniProtKB/Swiss-Prot:Q75KW7] | cytokinin-activated signaling pathway [GO:0009736] |
|  |  |  |  |  |  |  |  | TraesCS5A02G383900 | chr5A:581572707-581574621(-) | UDP-glucose flavonoid 3-O-glucosyltransferase 7 [UniProtKB/Swiss-Prot:Q2V6J9] | #N/A |
|  |  |  |  |  |  |  |  | TraesCS5A02G382900 | chr5A:580802616-580806190(-) | ATP sulfurylase 4, chloroplastic [UniProtKB/Swiss-Prot:Q9S7D8] | hydrogen sulfide biosynthetic process [GO:0070814] |
|  |  |  |  |  |  |  |  | TraesCS5A02G384200 | chr5A:582099388-582104496(+) | Serine/threonine-protein kinase STY13 [UniProtKB/Swiss-Prot:Q9ZQ31] | #N/A |
|  |  |  |  |  |  |  |  | TraesCS5A02G383100 | chr5A:580944043-580947242(+) | General transcription factor 3C polypeptide 3 [UniProtKB/Swiss-Prot:Q9Y5Q9] | #N/A |
| Q.CIM.stb.5AS.1 | AX-94958984 | 5A | 34526445 | 101.603 | 4.12179 |  | MLMSTB2019PC6 | NA | #N/A | #N/A |  |
| Q.CIM.stb.5BL.1 | RAC875_c19105_498 | 5B | 4.32E+08 | 52.1403 | 4.108635 |  | FarmCPUSTB2020 | TraesCS5B02G249700 | chr5B:432174789-432178198(-) | Rhamnogalacturonan I rhamnosyltransferase 1 [UniProtKB/Swiss-Prot:Q4V398] | fucose metabolic process |
| Q.CIM.stb.5BL.2 | AX-94746298 | 5B | 5.61E+08 | -35.3584 | 3.647536 |  | FarmCPUSTB2020 | TraesCS5B02G382600 | chr5B:561061500-561065007(-) | Linoleate 9S-lipoxygenase 2 [UniProtKB/Swiss-Prot:P29250] | protein binding and metal ion binding; |
| Q.CIM.stb.5BL.3 | wsnp_Ex_c2870_5296539 | 5B | 6.81E+08 | 0 | 4.264746 |  | BLINKSTB2020 | TraesCS5B02G516400 | chr5B:680604273-680606613(-) | Glycine-rich RNA-binding protein RZ1A [UniProtKB/Swiss-Prot:Q9LIN3] |  |
| Q.CIM.stb.6AL.4 | wsnp_Ra_c27787_37299352 | 6A | 5.44E+08 | -57.4459 | 5.877371 | 2.342159 | FarmCPUSTB2019 | TraesCS6A02G309100 | chr6A:544204000-544213402(-) | NA |  |
| Q.CIM.stb.6AL.5 | BobWhite_s67148_292 | 6A | 5.82E+08 | -256 | 4.71436 |  | BLINKSTB2019 | TraesCS6A02G350400 | chr6A:582248516-582254826(+) | Protein DETOXIFICATION 19 [UniProtKB/Swiss-Prot:Q9LUH2] |  |
| Q.CIM.stb.6AS.3 | tplb0030n22_541 | 6A | 80690723 | -49.3172 | 3.801858 |  | FarmCPU ACROSS YEARS | TraesCS6A02G111200 | chr6A:80686000-80691297(+) | Protein ABC transporter 1, mitochondrial [UniProtKB/Swiss-Prot:Q9SBB2] |  |
| Q.CIM.stb.6DL.2 | AX-94633926 | 6D | 3.7E+08 | 62.4236 | 3.543652 |  | BLINKSTB2019 | TraesCS6D02G261300 | chr6D:369657989-369660932(+) | NA |  |
| Q.CIM.stb.6DL.3 | AX-94519170 | 6D | 4.65E+08 | -74.1355 | 3.166026 |  | MLMSTB2019 PC6 | TraesCS6D02G388300 | chr6D:464735277-464737794(-) | Rop guanine nucleotide exchange factor 3 [UniProtKB/Swiss-Prot:A4IJ27] |  |
| Q.CIM.stb.6DS.1 | RFL_Contig4626_873 | 6D | 91819922 | -121.281 | 3.331273 |  | MLMSTB2019 PC6 | TraesCS6D02G127700 | chr6D:91819732-91820932(-) | Probable glutathione S-transferase GSTU6 [UniProtKB/Swiss-Prot:Q06398] | glutathione metabolic process |
| Q.CIM.stb.7AL.2 | IAAV6287 | 7A | 6.45E+08 | 100.6087 | 6.161142 | 7.533103 | FarmCPUSTB2019 | NA | #N/A | #N/A |  |
| Q.CIM.stb.7AL.7 | AX-94404953 | 7D | 6.3E+08 | -56.6594 | 3.567555 |  | FarmCPUSTB2020 | TraesCS7D02G539500 | chr7D:629823548-629826613(+) | Aquaporin NIP1-3 [UniProtKB/Swiss-Prot:Q0DK16] | transmembrane transport |
| Q.CIM.stb.7AS.1 | AX-95103403 | 7A | 1.18E+08 | -24.2719 | 5.148811 | 4.760621 | FarmCPUSTB2021 | TraesCS7A02G161600 | chr7A:118184773-118187573(-) | Putative pentatricopeptide repeat-containing protein At5g08310, mitochondrial [UniProtKB/Swiss-Prot:P0C8Q6] |  |
| Q.CIM.stb_dh.1AS.1 | AX-95154820 | 1A | 3120139 | 56.69181 | 4.008231 |  | FarmCPUSTB2019 | TraesCS1A02G005500 | chr1A:3119378-3122276(-) | NA |  |
| Q.CIM.stb_dh.7AL.5 | wsnp_Ex_c3611_6599309 | 7A | 6.75E+08 | -84.5661 | 3.127973 |  | MLMSTB2019 PC6 | TraesCS7A02G483600 | chr7A:675112899-675115741(-) | NA |  |
| Q.CIM.stb_ph.1BS.1 | Excalibur_c29734_1152 | 1B | 1.49E+08 | 1504 | 5.791748 | 1.997294 | BLINKSTB2019 | NA | #N/A | #N/A |  |
| Q.CIM.stb_ph.2AL.3 | Kukri_c35153_956 | 2A | 3.82E+08 | -48.254 | 6.780147 | 1.280588 | FarmCPU ACROSS YEARS | NA_A | #N/A | #N/A |  |
| Q.CIM.stb_ph.7AL.4 | wsnp_JD_c20555_18262260 | 7A | 6.74E+08 | 79.27304 | 3.279815 |  | MLMSTB2019 PC6 | TraesCS7A02G482200 | chr7A:674276643-674280959(-) | ALA-interacting subunit 3 [UniProtKB/Swiss-Prot:Q9SLK2] |  |

**Table S4:** The unique quantitative trait nucleotides (QTNs) controlling the plant height (PH) and days to heading (DH)

| **Trait** | **QTNs** | **SNP** | **Chr** | **Pos** | **P.value** | **MAF** | **nobs** | **H&B.P.Value** | **Effect** | **LOG P VALUE** | **Years/Models** |
| --- | --- | --- | --- | --- | --- | --- | --- | --- | --- | --- | --- |
| PH | Q.CIM.ph.1AS.1 | Tdurum_contig13095_97 | 1A | 149352649 | 1.24E-07 | 0.112717 | 173 | 0.000668 | 1.880259 | 6.906436 | FarmCPU_ POOL |
| PH | Q.CIM.ph.1AL.2 | Ex_c5759_628 | 1A | 345108212 | 0.000465 | 0.092486 | 173 | 0.676153 | 4.283705 | 3.332682 | MLM_ POOL |
|  |  |  |  |  | 2.91E-05 | 0.092486 | 173 | 0.156731 | 7.638126 | 4.536231 | MLM_2020 |
| PH | Q.CIM.ph.1AL.3 | RFL_Contig5001_1099 | 1A | 353916288 | 0.00058 | 0.098266 | 173 | 0.676153 | -4.46737 | 3.236564 | MLM_ POOL |
|  |  |  |  |  | 9.03E-05 | 0.098266 | 173 | 0.243121 | -7.53957 | 4.044535 | MLM_2020 |
| PH | Q.CIM.ph.1BS.1 | BS00087784_51 | 1B | 50777928 | 0.000904 | 0.054913 | 173 | 0.856474 | 6.577153 | 3.043832 | MLM_2019 |
| PH | Q.CIM.ph.1BS.2 | BS00084305_51 | 1B | 90992706 | 5.24E-06 | 0.101156 | 173 | 0.014108 | 400 | 5.280902 | BLINK_2020 |
|  |  |  |  |  | 1.92E-11 | 0.101156 | 173 | 2.06E-07 | -4.32435 | 10.71779 | FarmCPU_2020 |
| PH | Q.CIM.stb_ph.1BS.1 | Excalibur_c29734_1152 | 1B | 148507500 | 0.000289 | 0.375723 | 173 | 0.472279 | -2.42513 | 3.539315 | FarmCPU_2021 |
| PH | Q.CIM.ph.1DS.1 | wsnp_Ra_c2633_5017265 | 1D | 8610041 | 0.000744 | 0.196532 | 173 | 0.676153 | 1.82527 | 3.128191 | MLM_ POOL |
|  |  |  |  |  | 7.76E-05 | 0.196532 | 173 | 0.836386 | 3.182755 | 4.110011 | MLM_2021 |
| PH | Q.CIM.ph.2AL.1 | wsnp_Ra_c14112_22155451 | 2A | 130174835 | 1.62E-07 | 0.086705 | 173 | 0.000438 | 4.606443 | 6.789156 | FarmCPU_2019 |
| PH | Q.CIM.ph.2AL.2 | AX-95249962 | 2A | 364479573 | 1.49E-07 | 0.104046 | 173 | 0.000438 | 4.093891 | 6.825759 | FarmCPU_2019 |
| PH | Q.CIM.stb_ph.2AL.3 | Kukri_c35153_956 | 2A | 381722087 | 0.000312 | 0.352601 | 173 | 0.472279 | 2.43194 | 3.505172 | FarmCPU_2021 |
| PH | Q.CIM.ph.2AL.3 | BS00067806_51 | 2A | 564620529 | 1.06E-05 | 0.234104 | 173 | 0.018146 | -1.17649 | 4.976201 | FarmCPU_ POOL |
| PH | Q.CIM.ph.2AL.4 | Jagger_c1810_163 | 2A | 598967566 | 1.91E-12 | 0.289017 | 173 | 2.06E-08 | 0 | 11.71867 | BLINK_2021 |
|  |  |  |  |  | 0.000252 | 0.289017 | 173 | 0.472279 | -2.61143 | 3.598798 | FarmCPU_2021 |
|  |  |  |  |  | 0.000868 | 0.289017 | 173 | 0.916291 | -3.12309 | 3.061406 | MLM_2021 |
| PH | Q.CIM.ph.2AL.5 | wsnp_Ex_c8894_14858193 | 2A | 602440025 | 0.000273 | 0.367052 | 173 | 0.472279 | 2.438404 | 3.563459 | FarmCPU_2021 |
| PH | Q.CIM.ph.2AL.6 | BS00049644_51 | 2A | 605184906 | 0.000791 | 0.193642 | 173 | 0.916291 | -2.95848 | 3.101726 | MLM_2021 |
| PH | Q.CIM.ph.2BS.1 | RAC875_c27611_467 | 2B | 1251390 | 0.000236 | 0.176301 | 173 | 0.317222 | -1.98872 | 3.627963 | BLINK_2020 |
| PH | Q.CIM.ph.2BS.2 | AX-94831339 | 2B | 3405723 | 0.000215 | 0.17341 | 173 | 0.317222 | -2.01549 | 3.666779 | BLINK_2020 |
| PH | Q.CIM.ph.2BS.3 | RAC875_c62831_255 | 2B | 17564757 | 1.08E-06 | 0.080925 | 173 | 0.003861 | 3.105693 | 5.968561 | FarmCPU_2020 |
| PH | Q.CIM.ph.2BS.4 | AX-94397938 | 2B | 253716707 | 0.000939 | 0.066474 | 173 | 0.856474 | 4.59566 | 3.027476 | MLM_2019 |
| PH | Q.CIM.ph.3AS.1 | RFL_Contig3591_1832 | 3A | 22616253 | 0.00017 | 0.17341 | 173 | 0.30537 | -1.59731 | 3.76944 | BLINK _ POOL |
| PH | Q.CIM.ph.3AL.2 | RAC875_c27323_867 | 3A | 480438501 | 0.000476 | 0.15896 | 173 | 0.676153 | -2.77555 | 3.321978 | MLM_ POOL |
| PH | Q.CIM.ph.3AL.3 | BS00021930_51 | 3A | 514111005 | 8.48E-05 | 0.199422 | 173 | 0.241315 | -32 | 4.071379 | BLINK_2021 |
| PH | Q.CIM.ph.3AL.4 | Ku_c26872_269 | 3A | 729756576 | 0.000213 | 0.398844 | 173 | 0.460032 | 0 | 3.67066 | BLINK_2021 |
| PH | Q.CIM.ph.3BS.1 | IAAV3924 | 3B | 20450524 | 0.000252 | 0.184971 | 173 | 0.379218 | 1.531785 | 3.598897 | BLINK _ POOL |
| PH | Q.CIM.ph.3BS.2 | AX-95008150 | 3B | 20720572 | 6.67E-05 | 0.182081 | 173 | 0.235117 | 1.674723 | 4.176112 | BLINK _ POOL |
|  |  |  |  |  | 1.46E-06 | 0.182081 | 173 | 0.003938 | 1.489574 | 5.835044 | FarmCPU_ POOL |
| PH | Q.CIM.ph.3BL.3 | AX-94634237 | 3B | 449812045 | 0.0001 | 0.078035 | 173 | 0.235117 | -256 | 3.998216 | BLINK _ POOL |
|  |  |  |  |  | 3.56E-06 | 0.078035 | 173 | 0.007678 | 4.188779 | 5.448184 | FarmCPU_2019 |
| PH | Q.CIM.ph.3BL.4 | Excalibur_c15332_1194 | 3B | 578812799 | 0.000255 | 0.303468 | 173 | 0.30557 | -1.86195 | 3.593065 | FarmCPU_2019 |
| PH | Q.CIM.ph.3BL.5 | BS00023017_51 | 3B | 779366860 | 0.000149 | 0.138728 | 173 | 0.178131 | -1.84404 | 3.827434 | FarmCPU_2020 |
| PH | Q.CIM.ph.4AL.1 | wsnp_JD_rep_c51623_35119179 | 4A | 486124896 | 3.81E-05 | 0.135838 | 173 | 0.058705 | -2.74557 | 4.418645 | FarmCPU_2019 |
| PH | Q.CIM.ph.4AL.2 | Kukri_c52413_282 | 4A | 590588763 | 7.95E-07 | 0.066474 | 173 | 0.008565 | 51 | 6.099687 | BLINK_2019 |
|  |  |  |  |  | 1.74E-06 | 0.066474 | 173 | 0.014108 | 48 | 5.76004 | BLINK_2020 |
|  |  |  |  |  | 1.18E-05 | 0.066474 | 173 | 0.018146 | -2.18349 | 4.928531 | FarmCPU_ POOL |
|  |  |  |  |  | 2.88E-06 | 0.066474 | 173 | 0.007755 | -3.75875 | 5.540794 | FarmCPU_2020 |
|  |  |  |  |  | 0.000209 | 0.066474 | 173 | 0.676153 | -2.97327 | 3.680189 | MLM_ POOL |
|  |  |  |  |  | 0.000671 | 0.066474 | 173 | 0.856474 | -4.96172 | 3.173058 | MLM_2019 |
|  |  |  |  |  | 1.76E-06 | 0.066474 | 173 | 0.018913 | -5.85972 | 5.75565 | MLM_2020 |
| PH | Q.CIM.ph.4BL.1 | BobWhite_rep_c66957_84 | 4B | 599553452 | 0.000326 | 0.228324 | 173 | 0.856474 | 4.930359 | 3.486379 | MLM_2019 |
| PH | Q.CIM.ph.5AL.1 | Ex_c1344_1859 | 5A | 104625383 | 0.000109 | 0.080925 | 173 | 0.235117 | 48 | 3.962163 | BLINK _ POOL |
|  |  |  |  |  | 0.000165 | 0.080925 | 173 | 0.295752 | 68 | 3.783339 | BLINK_2020 |
|  |  |  |  |  | 2.08E-08 | 0.080925 | 173 | 0.000224 | -3.01449 | 7.681644 | FarmCPU_ POOL |
|  |  |  |  |  | 1.04E-06 | 0.080925 | 173 | 0.003861 | -3.25791 | 5.983237 | FarmCPU_2020 |
| PH | Q.CIM.ph.5AL.2 | wsnp_Ex_c2132_4004831 | 5A | 403353551 | 0.000286 | 0.112717 | 173 | 0.30869 | -3.72027 | 3.542895 | MLM_2020 |
| PH | Q.CIM.ph.5AL.3 | wsnp_Ex_c40022_47169698 | 5A | 404533251 | 0.000286 | 0.112717 | 173 | 0.30869 | -3.72027 | 3.542895 | MLM_2020 |
| PH | Q.CIM.ph.5AL.4 | wsnp_BE443187A_Ta_2_3 | 5A | 450164758 | 5.06E-05 | 0.294798 | 173 | 0.09019 | -1.30799 | 4.295902 | FarmCPU_2020 |
| PH | Q.CIM.ph.5AL.5 | wsnp_Ex_c19724_28721128 | 5A | 503137639 | 2.02E-08 | 0.196532 | 173 | 0.000109 | 80 | 7.69515 | BLINK_2021 |
|  |  |  |  |  | 0.00022 | 0.196532 | 173 | 0.472279 | -3.14621 | 3.658225 | FarmCPU_2021 |
|  |  |  |  |  | 0.000648 | 0.196532 | 173 | 0.916291 | -3.18046 | 3.188314 | MLM_2021 |
| PH | Q.CIM.ph.5AL.6 | IACX5390 | 5A | 605101626 | 0.000104 | 0.286127 | 173 | 0.139814 | -1.30761 | 3.983777 | FarmCPU_2020 |
| PH | Q.CIM.ph.5BS.1 | RAC875_rep_c116173_605 | 5B | 35289978 | 2.72E-06 | 0.153179 | 173 | 0.014108 | 56 | 5.565291 | BLINK_2020 |
| PH | Q.CIM.ph.5BS.2 | Excalibur_c14594_182 | 5B | 38157606 | 0.000123 | 0.265896 | 173 | 0.147323 | 0.950386 | 3.909904 | FarmCPU_ POOL |
|  |  |  |  |  | 1.18E-07 | 0.265896 | 173 | 0.000438 | 2.956642 | 6.929789 | FarmCPU_2019 |
|  |  |  |  |  | 0.00071 | 0.265896 | 173 | 0.676153 | 1.853555 | 3.149024 | MLM_ POOL |
|  |  |  |  |  | 0.000423 | 0.265896 | 173 | 0.856474 | 3.486181 | 3.373877 | MLM_2019 |
| PH | Q.CIM.ph.5BS.3 | AX-94606697 | 5B | 59162669 | 2.54E-05 | 0.228324 | 173 | 0.137107 | 68 | 4.594328 | BLINK _ POOL |
|  |  |  |  |  | 0.000847 | 0.228324 | 173 | 0.676153 | -1.91873 | 3.072271 | MLM_ POOL |
| PH | Q.CIM.ph.5BL.4 | Kukri_c36789_230 | 5B | 355618301 | 0.000286 | 0.112717 | 173 | 0.30869 | 3.72027 | 3.542895 | MLM_2020 |
| PH | Q.CIM.ph.5BL.5 | wsnp_Ex_c5632_9904112 | 5B | 356186273 | 0.000262 | 0.115607 | 173 | 0.30869 | -3.73498 | 3.581421 | MLM_2020 |
| PH | Q.CIM.ph.5DL.1 | wsnp_Ex_c19724_28720939 | 5D | 398107597 | 0.000254 | 0.187861 | 173 | 0.472279 | -3.09487 | 3.594409 | FarmCPU_2021 |
| PH | Q.CIM.ph.6AS.1 | Kukri_c21943_958 | 6A | 70419120 | 3.23E-05 | 0.320809 | 173 | 0.058064 | 1.779232 | 4.490358 | FarmCPU_2019 |
| PH | Q.CIM.ph.6AS.2 | RAC875_c17189_155 | 6A | 105181461 | 2.15E-07 | 0.095376 | 173 | 0.00232 | 384 | 6.666966 | BLINK _ POOL |
|  |  |  |  |  | 2.07E-06 | 0.095376 | 173 | 0.004462 | 1.946008 | 5.683876 | FarmCPU_ POOL |
|  |  |  |  |  | 7.95E-06 | 0.095376 | 173 | 0.017122 | 2.982801 | 5.099883 | FarmCPU_2020 |
|  |  |  |  |  | 6.46E-05 | 0.095376 | 173 | 0.231943 | 4.374366 | 4.189915 | MLM_2020 |
| PH | Q.CIM.ph.6AL.3 | Kukri_rep_c104521_601 | 6A | 564270705 | 3.22E-05 | 0.144509 | 173 | 0.069362 | 64 | 4.492323 | BLINK_2020 |
|  |  |  |  |  | 5.86E-05 | 0.144509 | 173 | 0.09019 | -2.09869 | 4.232162 | FarmCPU_2020 |
|  |  |  |  |  | 0.000269 | 0.144509 | 173 | 0.30869 | -3.48257 | 3.571037 | MLM_2020 |
| PH | Q.CIM.ph.6AL.4 | Kukri_c6128_319 | 6A | 564272520 | 0.000269 | 0.144509 | 173 | 0.30869 | -3.48257 | 3.571037 | MLM_2020 |
| PH | Q.CIM.ph.6AL.5 | BobWhite_c5255_429 | 6A | 603280457 | 0.000301 | 0.095376 | 173 | 0.324042 | 2.751589 | 3.521816 | FarmCPU_2019 |
| PH | Q.CIM.ph.6BL.1 | wsnp_Ex_c5177_9174930 | 6B | 694678780 | 5.04E-05 | 0.104046 | 173 | 0.067884 | 1.494412 | 4.297562 | FarmCPU_ POOL |
| PH | Q.CIM.ph.7AS.1 | BobWhite_c14495_230 | 7A | 14189397 | 4.86E-06 | 0.297688 | 173 | 0.014108 | 72 | 5.313139 | BLINK_2020 |
| PH | Q.CIM.ph.7AS.2 | wsnp_Ex_c23755_32994701 | 7A | 95806762 | 0.000312 | 0.300578 | 173 | 0.591222 | 57 | 3.506103 | BLINK_2019 |
| PH | Q.CIM.ph.7AS.3 | Ku_c9598_2119 | 7A | 205171484 | 2.66E-07 | 0.49422 | 173 | 0.000957 | -1.22738 | 6.574597 | FarmCPU_ POOL |
| PH | Q.CIM.ph.7AL.4 | wsnp_Ku_c17161_26193994 | 7A | 660450478 | 7.02E-05 | 0.210983 | 173 | 0.094607 | 2.038451 | 4.153405 | FarmCPU_2019 |
| PH | Q.CIM.stb_ph.7AL.4 | wsnp_JD_c20555_18262260 | 7A | 674276749 | 0.0009 | 0.312139 | 173 | 0.676153 | -1.64912 | 3.045715 | MLM_ POOL |
| PH | Q.CIM.ph.7AL.5 | wsnp_JD_c20555_18262317 | 7A | 674276806 | 0.000826 | 0.34104 | 173 | 0.676153 | 1.590347 | 3.083138 | MLM_ POOL |
| PH | Q.CIM.ph.7BS.1 | Ra_c56305_1946 | 7B | 159142231 | 1.30E-07 | 0.488439 | 173 | 0.000438 | -2.42142 | 6.885147 | FarmCPU_2019 |
| PH | Q.CIM.ph.7BL.2 | AX-94767893 | 7B | 741575987 | 0.000414 | 0.390173 | 173 | 0.676153 | 1.740548 | 3.38252 | MLM_ POOL |
| PH | Q.CIM.ph.7BL.3 | Tdurum_contig30909_76 | 7B | 743304117 | 8.96E-05 | 0.393064 | 173 | 0.241315 | 48 | 4.047774 | BLINK_2021 |
| DH | Q.CIM.stb_dh.1AS.1 | AX-95154820 | 1A | 3120139 | 0.000262 | 0.141618 | 173 | 0.831425 | 36 | 3.581285 | BLINK_2019 |
|  |  |  |  |  | 4.48E-06 | 0.141618 | 173 | 0.013697 | -1.29993 | 5.349141 | FarmCPU_2019 |
|  |  |  |  |  | 0.000567 | 0.141618 | 173 | 0.96198 | -2.08589 | 3.246752 | MLM_2019 |
| DH | Q.CIM.dh.1AS.1 | RAC875_c38756_141 | 1A | 7335009 | 2.78E-05 | 0.268786 | 173 | 0.03333 | -0.68923 | 4.555343 | FarmCPU_2020 |
| DH | Q.CIM.dh.1AL.1 | Excalibur_c59894_97 | 1A | 463813596 | 0.000587 | 0.066474 | 173 | 0.646916 | 2.07606 | 3.231537 | MLM_POOL |
|  |  |  |  |  | 0.000374 | 0.066474 | 173 | 0.686823 | 2.928048 | 3.426922 | MLM_2021 |
| DH | Q.CIM.dh.1AL.2 | AX-94550326 | 1A | 464116406 | 0.000876 | 0.063584 | 173 | 0.674388 | -2.18664 | 3.05738 | MLM_POOL |
|  |  |  |  |  | 0.000995 | 0.063584 | 173 | 0.809624 | -2.93403 | 3.002204 | MLM_2021 |
| DH | Q.CIM.dh.1AL.3 | Ra_c58315_265 | 1A | 473637106 | 0.000258 | 0.054913 | 173 | 0.397376 | 416 | 3.588117 | BLINK_2021 |
|  |  |  |  |  | 1.40E-05 | 0.054913 | 173 | 0.018887 | 1.839411 | 4.853168 | FarmCPU_2021 |
| DH | Q.CIM.dh.1AL.4 | AX-94408000 | 1A | 550444223 | 0.000245 | 0.476879 | 173 | 0.240062 | 0.523558 | 3.610701 | FarmCPU_POOL |
| DH | Q.CIM.dh.1AL.5 | AX-94796921 | 1B | 38067645 | 4.79E-06 | 0.130058 | 173 | 0.013697 | 1.294109 | 5.319593 | FarmCPU_2019 |
| DH | Q.CIM.dh.1BL.1 | wsnp_Ex_c1440_2764867 | 1B | 551177422 | 1.95E-05 | 0.33526 | 173 | 0.105294 | 120 | 4.708983 | BLINK_2020 |
|  |  |  |  |  | 9.27E-05 | 0.33526 | 173 | 0.090827 | -0.54843 | 4.03281 | FarmCPU_2020 |
| DH | Q.CIM.dh.1BL.2 | TA004668-0687 | 1B | 643097047 | 1.18E-06 | 0.398844 | 173 | 0.012745 | -0.91781 | 5.927072 | FarmCPU_2019 |
| DH | Q.CIM.dh.2AS.1 | Kukri_c3701_392 | 2A | 1370472 | 1.80E-05 | 0.488439 | 173 | 0.038843 | 70 | 4.744135 | BLINK_2021 |
| DH | Q.CIM.dh.2AL.2 | RAC875_c96033_191 | 2A | 753617305 | 3.47E-08 | 0.312139 | 173 | 0.000187 | -0.77318 | 7.460227 | FarmCPU_POOL |
| DH | Q.CIM.dh.2AL.3 | BS00091763_51 | 2A | 773185993 | 0.000606 | 0.306358 | 173 | 0.646916 | -0.94778 | 3.217629 | MLM_POOL |
|  |  |  |  |  | 0.000249 | 0.306358 | 173 | 0.66943 | -1.23805 | 3.604652 | MLM_2020 |
| DH | Q.CIM.dh.2BL.1 | Excalibur_c33267_538 | 2B | 729180151 | 0.000498 | 0.138728 | 173 | 0.646916 | -1.23543 | 3.302539 | MLM_POOL |
| DH | Q.CIM.dh.2BL.2 | Excalibur_c44325_638 | 2B | 741445277 | 4.91E-06 | 0.268786 | 173 | 0.010113 | 0.873208 | 5.309011 | FarmCPU_2021 |
| DH | Q.CIM.stb_dh.2BL.9 | AX-95084761 | 2B | 784545663 | 0.000672 | 0.213873 | 173 | 0.646916 | 1.086337 | 3.172749 | MLM_POOL |
|  |  |  |  |  | 0.000183 | 0.213873 | 173 | 0.657027 | 1.659583 | 3.737713 | MLM_2021 |
| DH | Q.CIM.dh.2BL.3 | BS00004413_51 | 2B | 784551315 | 4.79E-05 | 0.075145 | 173 | 0.258312 | -2.0375 | 4.319243 | MLM_POOL |
|  |  |  |  |  | 0.000931 | 0.075145 | 173 | 0.96198 | -2.14786 | 3.031024 | MLM_2019 |
|  |  |  |  |  | 7.82E-06 | 0.075145 | 173 | 0.042114 | -3.11265 | 5.106958 | MLM_2021 |
| DH | Q.CIM.stb_dh.2DL.3 | AX-94787485 | 2D | 590930925 | 2.53E-06 | 0.179191 | 173 | 0.004749 | 44 | 5.596513 | BLINK_POOL |
| DH | Q.CIM.dh.3AS.1 | wsnp_Ra_c2228_4310870 | 3A | 140046639 | 2.64E-06 | 0.101156 | 173 | 0.004749 | 0 | 5.577701 | BLINK_POOL |
| DH | Q.CIM.dh.3AL.1 | Ra_c4373_453 | 3A | 720436380 | 0.000153 | 0.138728 | 173 | 0.831425 | -1.31323 | 3.816416 | BLINK_2019 |
|  |  |  |  |  | 0.000407 | 0.138728 | 173 | 0.646916 | -1.24891 | 3.389931 | MLM_POOL |
|  |  |  |  |  | 1.59E-05 | 0.138728 | 173 | 0.170931 | -2.02562 | 4.799596 | MLM_2019 |
| DH | Q.CIM.stb_dh.3AL.6 | RAC875_c61934_186 | 3A | 733995575 | 5.63E-06 | 0.236994 | 173 | 0.010113 | 0.94587 | 5.249372 | FarmCPU_2021 |
| DH | Q.CIM.dh.3BS.1 | AX-94660868 | 3B | 4328430 | 0.000113 | 0.124277 | 173 | 0.122035 | 0.988455 | 3.945932 | FarmCPU_POOL |
| DH | Q.CIM.dh.3BL.2 | Kukri_c66862_96 | 3B | 745220324 | 0.000637 | 0.473988 | 173 | 0.686533 | 1.062318 | 3.195756 | MLM_2020 |
| DH | Q.CIM.dh.3BL.3 | Ku_c24974_674 | 3B | 747712577 | 8.12E-09 | 0.485549 | 173 | 4.38E-05 | 74 | 8.090208 | BLINK_POOL |
|  |  |  |  |  | 1.36E-06 | 0.485549 | 173 | 0.004891 | 68 | 5.865859 | BLINK_2021 |
|  |  |  |  |  | 9.42E-08 | 0.485549 | 173 | 0.000338 | 0.748358 | 7.025827 | FarmCPU_POOL |
|  |  |  |  |  | 0.000191 | 0.485549 | 173 | 0.147106 | 0.628093 | 3.718658 | FarmCPU_2020 |
|  |  |  |  |  | 0.00036 | 0.485549 | 173 | 0.646916 | 0.986151 | 3.443489 | MLM_POOL |
|  |  |  |  |  | 3.76E-05 | 0.485549 | 173 | 0.404636 | 1.239362 | 4.425353 | MLM_2020 |
| DH | Q.CIM.dh.3BL.4 | AX-94439075 | 3B | 756265102 | 0.000217 | 0.381503 | 173 | 0.66943 | -1.1026 | 3.664237 | MLM_2020 |
| DH | Q.CIM.dh.3BL.5 | AX-94756413 | 3B | 791287062 | 0.000372 | 0.294798 | 173 | 0.96198 | -1.51154 | 3.429405 | MLM_2019 |
| DH | Q.CIM.dh.3DL.1 | AX-95165551 | 3D | 569524161 | 0.000147 | 0.419075 | 173 | 0.121943 | -0.6348 | 3.832316 | FarmCPU_2020 |
|  |  |  |  |  | 0.000182 | 0.419075 | 173 | 0.66943 | -1.05019 | 3.739432 | MLM_2020 |
| DH | Q.CIM.dh.4AS.1 | BS00068244_51 | 4A | 46125511 | 1.56E-08 | 0.066474 | 173 | 5.62E-05 | 34 | 7.805663 | BLINK_POOL |
|  |  |  |  |  | 7.78E-06 | 0.066474 | 173 | 0.02096 | 35 | 5.108962 | BLINK_2021 |
|  |  |  |  |  | 1.92E-05 | 0.066474 | 173 | 0.025824 | -1.36005 | 4.717306 | FarmCPU_POOL |
| DH | Q.CIM.dh.4AS.2 | wsnp_Ex_c6044_10590220 | 4A | 101510364 | 2.70E-07 | 0.17341 | 173 | 0.000728 | -0.97806 | 6.568239 | FarmCPU_POOL |
| DH | Q.CIM.dh.4AL.3 | BS00072025_51 | 4A | 605664279 | 1.04E-08 | 0.083815 | 173 | 0.000112 | 1.76435 | 7.982171 | FarmCPU_2020 |
| DH | Q.CIM.dh.4AL.4 | wsnp_Ex_c19207_28125072 | 4A | 611580737 | 2.28E-05 | 0.193642 | 173 | 0.040885 | -0.98435 | 4.642707 | FarmCPU_2019 |
| DH | Q.CIM.dh.4BL.1 | RAC875_c1357_860 | 4B | 609498098 | 5.08E-06 | 0.352601 | 173 | 0.013697 | 0.935393 | 5.293724 | FarmCPU_2019 |
| DH | Q.CIM.dh.4DS.1 | IAAV1674 | 4D | 1876348 | 9.34E-07 | 0.251445 | 173 | 0.002516 | 46 | 6.029674 | BLINK_POOL |
|  |  |  |  |  | 1.14E-07 | 0.251445 | 173 | 0.000616 | 44 | 6.941515 | BLINK_2021 |
|  |  |  |  |  | 2.67E-07 | 0.251445 | 173 | 0.001439 | 1.026527 | 6.573402 | FarmCPU_2021 |
| DH | Q.CIM.dh.5AS.1 | IAAV5061 | 5A | 91119501 | 1.76E-05 | 0.092486 | 173 | 0.02107 | -5.28491 | 4.754513 | BLINK_POOL |
| DH | Q.CIM.dh.5AS.2 | Ku_c69633_1873 | 5A | 97227265 | 1.76E-05 | 0.092486 | 173 | 0.02107 | -5.28491 | 4.754513 | BLINK_POOL |
| DH | Q.CIM.dh.5AS.3 | wsnp_Ku_c328_679106 | 5A | 104232188 | 1.76E-05 | 0.092486 | 173 | 0.02107 | -5.28491 | 4.754513 | BLINK_POOL |
| DH | Q.CIM.dh.5AL.4 | Excalibur_c1208_72 | 5A | 502219443 | 8.95E-05 | 0.369942 | 173 | 0.107193 | 0.453575 | 4.048009 | FarmCPU_POOL |
| DH | Q.CIM.dh.5AL.5 | BS00005860_51 | 5A | 598245820 | 0.000446 | 0.323699 | 173 | 0.686823 | -1.64331 | 3.350987 | MLM_2021 |
| DH | Q.CIM.dh.5AL.6 | BS00021968_51 | 5A | 677631865 | 0.000597 | 0.456647 | 173 | 0.96198 | -1.35866 | 3.223752 | MLM_2019 |
| DH | Q.CIM.dh.5BS.1 | Kukri_c1468_1609 | 5B | 65772685 | 3.52E-05 | 0.248555 | 173 | 0.063155 | 128 | 4.45386 | BLINK_2021 |
|  |  |  |  |  | 0.000815 | 0.248555 | 173 | 0.809624 | 1.413648 | 3.088604 | MLM_2021 |
| DH | Q.CIM.dh.5BL.2 | AX-94435238 | 5B | 581132597 | 0.0003 | 0.075145 | 173 | 0.59812 | 32 | 3.523524 | BLINK_2020 |
|  |  |  |  |  | 4.87E-06 | 0.075145 | 173 | 0.007814 | -1.28364 | 5.312391 | FarmCPU_POOL |
|  |  |  |  |  | 4.54E-05 | 0.075145 | 173 | 0.048891 | -1.3445 | 4.343192 | FarmCPU_2021 |
| DH | Q.CIM.dh.5BS.1 | AX-94663690 | 5D | 45354865 | 4.73E-07 | 0.17052 | 173 | 0.001698 | -1.23448 | 6.325339 | FarmCPU_2021 |
| DH | Q.CIM.dh.6AL.1 | Tdurum_contig97355_136 | 6A | 591729142 | 0.000581 | 0.291908 | 173 | 0.646916 | -0.98069 | 3.236097 | MLM_POOL |
|  |  |  |  |  | 0.00085 | 0.291908 | 173 | 0.809624 | -1.34648 | 3.070719 | MLM_2021 |
| DH | Q.CIM.dh.6AL.2 | Tdurum_contig97355_194 | 6A | 591729183 | 0.00072 | 0.283237 | 173 | 0.646916 | 0.946336 | 3.142388 | MLM_POOL |
| DH | Q.CIM.dh.6AL.2 | Excalibur_c2737_309 | 6A | 591732019 | 0.000408 | 0.286127 | 173 | 0.646916 | 0.998431 | 3.389104 | MLM_POOL |
|  |  |  |  |  | 0.000333 | 0.286127 | 173 | 0.686823 | 1.43775 | 3.477861 | MLM_2021 |
| DH | Q.CIM.dh.6AL.3 | Excalibur_c25390_2483 | 6A | 605231868 | 2.26E-05 | 0.343931 | 173 | 0.030469 | 0.633782 | 4.645463 | FarmCPU_2020 |
| DH | Q.CIM.dh.6AL.4 | RAC875_c12821_466 | 6A | 615439091 | 1.79E-06 | 0.187861 | 173 | 0.00385 | 0.952823 | 5.747936 | FarmCPU_2020 |
| DH | Q.CIM.dh.6AL.5 | Tdurum_contig70819_393 | 6A | 615821812 | 0.000291 | 0.066474 | 173 | 0.309097 | -1.11338 | 3.536302 | BLINK_POOL |
| DH | Q.CIM.dh.6BS.1 | AX-94408875 | 6B | 23653379 | 1.88E-11 | 0.078035 | 173 | 2.03E-07 | 41 | 10.72517 | BLINK_POOL |
|  |  |  |  |  | 7.32E-14 | 0.078035 | 173 | 7.89E-10 | 44 | 13.13553 | BLINK_2021 |
|  |  |  |  |  | 8.58E-16 | 0.078035 | 173 | 9.24E-12 | -2.24175 | 15.06652 | FarmCPU_POOL |
|  |  |  |  |  | 1.83E-05 | 0.078035 | 173 | 0.039442 | -1.28236 | 4.737484 | FarmCPU_2019 |
|  |  |  |  |  | 2.04E-13 | 0.078035 | 173 | 2.20E-09 | -2.63482 | 12.69049 | FarmCPU_2021 |
|  |  |  |  |  | 4.58E-06 | 0.078035 | 173 | 0.049397 | -2.41013 | 5.338713 | MLM_POOL |
|  |  |  |  |  | 3.27E-05 | 0.078035 | 173 | 0.175938 | -2.81931 | 4.486029 | MLM_2019 |
|  |  |  |  |  | 0.000534 | 0.078035 | 173 | 0.686533 | -1.90883 | 3.272678 | MLM_2020 |
|  |  |  |  |  | 3.12E-06 | 0.078035 | 173 | 0.03364 | -3.37524 | 5.505559 | MLM_2021 |
| DH | Q.CIM.dh.6BS.2 | wsnp_Ex_rep_c115803_95396724 | 6B | 25256244 | 0.000108 | 0.465318 | 173 | 0.096536 | -0.55862 | 3.968545 | FarmCPU_2020 |
| DH | Q.CIM.dh.6BL.3 | RAC875_c68525_284 | 6B | 657946476 | 1.55E-07 | 0.150289 | 173 | 0.000557 | -1.06052 | 6.809469 | FarmCPU_2020 |
| DH | Q.CIM.dh.6DS.1 | wsnp_Ex_c14439_22426200 | 6D | 2406518 | 6.17E-05 | 0.482659 | 173 | 0.066524 | -0.67278 | 4.20944 | FarmCPU_2020 |
|  |  |  |  |  | 0.000805 | 0.482659 | 173 | 0.666953 | -0.83462 | 3.094379 | MLM_POOL |
|  |  |  |  |  | 0.000463 | 0.482659 | 173 | 0.686533 | -0.97516 | 3.334252 | MLM_2020 |
| DH | Q.CIM.dh.6DS.2 | TA002853-0110-w | 6D | 2408966 | 0.000523 | 0.459538 | 173 | 0.686533 | 0.916224 | 3.281446 | MLM_2020 |
| DH | Q.CIM.dh.6DL.3 | AX-94765421 | 6D | 445497018 | 0.000446 | 0.291908 | 173 | 0.686823 | -1.33071 | 3.350474 | MLM_2021 |
| DH | Q.CIM.dh.7AS.1 | Tdurum_contig93663_457 | 7A | 18876986 | 5.08E-06 | 0.476879 | 173 | 0.007814 | -0.62214 | 5.294464 | FarmCPU_POOL |
| DH | Q.CIM.dh.7AS.2 | Tdurum_contig12722_779 | 7A | 46720779 | 2.36E-06 | 0.346821 | 173 | 0.004239 | -0.82025 | 5.627013 | FarmCPU_2020 |
| DH | Q.CIM.dh.7AS.3 | AX-94388916 | 7A | 58238172 | 2.47E-08 | 0.17052 | 173 | 0.000133 | -1.03199 | 7.607723 | FarmCPU_2020 |
| DH | Q.CIM.dh.7AL.4 | IAAV6170 | 7A | 538784257 | 0.000506 | 0.098266 | 173 | 0.96198 | -1.96278 | 3.29583 | MLM_2019 |
| DH | Q.CIM.dh.7AL.5 | wsnp_Ku_c60707_62509051 | 7A | 659372722 | 1.43E-05 | 0.277457 | 173 | 0.022028 | 0.540892 | 4.844351 | FarmCPU_2020 |
| DH | Q.CIM.dh.7AL.6 | BS00009838_51 | 7A | 666607524 | 4.87E-06 | 0.072254 | 173 | 0.010113 | 1.340472 | 5.312081 | FarmCPU_2021 |
| DH | Q.CIM.stb_dh.7AL.5 | wsnp_Ex_c3611_6599309 | 7A | 675113102 | 2.88E-05 | 0.147399 | 173 | 0.034486 | 0.95936 | 4.540533 | FarmCPU_2021 |
| DH | Q.CIM.dh.7AL.7 | wsnp_Ex_rep_c101269_86664147 | 7A | 704793025 | 7.00E-06 | 0.132948 | 173 | 0.01078 | -1.03245 | 5.154683 | FarmCPU_2021 |
| DH | Q.CIM.dh.7AL.8 | BS00010819_51 | 7A | 723259910 | 1.57E-05 | 0.179191 | 173 | 0.105294 | 42 | 4.803512 | BLINK_2020 |
|  |  |  |  |  | 1.21E-06 | 0.179191 | 173 | 0.003261 | -1.00887 | 5.917012 | FarmCPU_2020 |
|  |  |  |  |  | 0.000543 | 0.179191 | 173 | 0.686533 | -1.36456 | 3.264954 | MLM_2020 |
| DH | Q.CIM.dh.7BS.1 | AX-94545252 | 7B | 133792834 | 0.000971 | 0.199422 | 173 | 0.809624 | -2.19859 | 3.012962 | MLM_2021 |
| DH | Q.CIM.dh.7BL.2 | Excalibur_c25630_537 | 7B | 665679233 | 7.32E-07 | 0.315029 | 173 | 0.001578 | -0.67898 | 6.135337 | FarmCPU_POOL |
| DH | Q.CIM.dh.7BL.3 | BobWhite_c25708_334 | 7B | 727634253 | 0.000494 | 0.141618 | 173 | 0.646916 | -1.36449 | 3.306069 | MLM_POOL |
|  |  |  |  |  | 0.000575 | 0.141618 | 173 | 0.686533 | -1.43472 | 3.240369 | MLM_2020 |

**Table S5:** QTNs in complete linkage across the genome

| **QTNs** | **Chromosome** | **Position1** | **QTLs (Position 2)** | **Chromosome** | **Position2** | **Dist_bp** | **R^2** | **DPrime** | **pDiseq** |
| --- | --- | --- | --- | --- | --- | --- | --- | --- | --- |
| Q.CIM.stb.1AL.8 | 1A | 5.75E+08 | Q.CIM.stb.1AL.7 | 1A | 5.75E+08 | 115 | 1 | 1 | 9.3E-20 |
| Q.CIM.stb.1AL.7 | 1A | 5.75E+08 | Q.CIM.stb.1AL.6 | 1A | 5.75E+08 | 104490 | 1 | 1 | 1.01E-19 |
| Q.CIM.stb.1AL.8 | 1A | 5.75E+08 | Q.CIM.stb.1AL.6 | 1A | 5.75E+08 | 104605 | 1 | 1 | 9.3E-20 |
| Q.CIM.stb_ph.2AL.3 | 2A | 3.82E+08 | Q.CIM.stb.4AL.4 | 1B | 1.49E+08 | N/A | 1 | 1 | 1.6E-43 |
| Q.CIM.stb.3AL.3 | 3A | 5.35E+08 | Q.CIM.stb.3AL.2 | 3A | 5.35E+08 | 934 | 1 | 1 | 1.42E-18 |
| Q.CIM.stb.3AL.4 | 3A | 5.38E+08 | Q.CIM.stb.3AL.3 | 3A | 5.35E+08 | 2313420 | 1 | 1 | 1.32E-18 |
| Q.CIM.stb.3AL.4 | 3A | 5.38E+08 | Q.CIM.stb.3AL.2 | 3A | 5.35E+08 | 2314354 | 1 | 1 | 1.42E-18 |
| Q.CIM.stb.3BL.4 | 3B | 5.41E+08 | Q.CIM.stb.3AL.2 | 3A | 5.35E+08 | N/A | 1 | 1 | 1.98E-17 |
| Q.CIM.stb.3BL.4 | 3B | 5.41E+08 | Q.CIM.stb.3AL.3 | 3A | 5.35E+08 | N/A | 1 | 1 | 1.85E-17 |
| Q.CIM.stb.3BL.4 | 3B | 5.41E+08 | Q.CIM.stb.3AL.4 | 3A | 5.38E+08 | N/A | 1 | 1 | 1.73E-17 |
| Q.CIM.stb.4AL.4 | 4A | 5.99E+08 | Q.CIM.stb.4AL.3 | 4A | 5.99E+08 | 27 | 1 | 1 | 2.17E-26 |
| Q.CIM.stb.4AL.3 | 4A | 5.99E+08 | Q.CIM.stb.4AL.2 | 4A | 5.99E+08 | 734583 | 0.896888 | 1 | 5.01E-24 |
| Q.CIM.stb.4AL.4 | 4A | 5.99E+08 | Q.CIM.stb.4AL.2 | 4A | 5.99E+08 | 734610 | 0.896888 | 1 | 5.01E-24 |
| Q.CIM.stb.4BL.4 | 4B | 6.5E+08 | Q.CIM.stb.4BL.3 | 4B | 6.49E+08 | 132031 | 0.951537 | 1 | 3.58E-27 |
| Q.CIM.stb.4BL.6 | 4B | 6.57E+08 | Q.CIM.stb.4BL.5 | 4B | 6.57E+08 | 268258 | 1 | 1 | 0 |
| Q.CIM.stb.5AL.7 | 5A | 5.73E+08 | Q.CIM.stb.5AL.6 | 5A | 5.72E+08 | 677492 | 0.948951 | 1 | 7.78E-43 |
| Q.CIM.stb.5AL.11 | 5A | 5.89E+08 | Q.CIM.stb.5AL.10 | 5A | 5.87E+08 | 1945657 | 1 | 1 | 6.87E-32 |
| Q.CIM.stb.5AL.8 | 5A | 5.74E+08 | Q.CIM.stb.5AL.7 | 5A | 5.73E+08 | 544965 | 0.80321 | 1 | 2.33E-34 |

**Table S6:** The physical position of reported Stb genes and Meta QTLs

| **Gene/MQTL** | **chr** | **Physical position (bp)** | | **Markers** | **Reference** |
| --- | --- | --- | --- | --- | --- |
| Stb1 | 5B | 402787130 |  | (Xbarc74;) | Adhikari et al. 2004a |
| Stb11 | 1B | 42329007 |  | barc8 | Chartrain et. al., 2005 |
| Stb12 | 4A | 732902997 |  | (Xwmc219;) | Chartrain et al., 2005c |
| Stb13 | 7B | 546855428 |  | (Xwmc396;) | Cowling 2006 |
| Stb14 | 3B | 27530271 |  | (wmc500;wmc632) | Cowling, 2006 |
| Stb16 | 3D | 590040459 |  | - | Saintenac, et al., 2021 |
| Stb17 | 5A | 579827316 |  | (gwm617;hbg291)hbg247, | Ghaffary et al.., 2012 |
| Stb18 | 6D | 21365827 |  | (gpw5176;gpw3087, 26130831 ) | Ghaffary et al., 2011 |
| Stb19 | 1D | 7340804 |  | (1218021, ;4909967, 7420420 ) | Yang et al., 2018 |
| Stb2 | 1B | 42183529 |  | (wmc406;barc8)wmc230 | Liu et al., 2013 |
| Stb3 | 7A | 89970411 |  | TPt-4209 (wPt-1664, wPt-9132, wPt-0864) | Goodwin and Thompson, 2011 |
| Stb4 | 7D | 13460405 |  | gwm111 | Adhikari et al., 2004 |
| Stb6 | 3A | 26200000 |  | - | Saintenac et al., 2018 |
| Stb7 | 4A | 739515101 |  | (Xwmc313;) | McCartney et al. 2003 |
| Stb8 | 7B | 740720573 |  | (Xgwm146;) | Adhikari et al. 2003 |
| Stb9 | 2B | 739396089 |  | (wmc332;gpw1214, 786618055) | Chartrain et. al., 2009 |
| Stbsm3 | 3A | 11685754 |  | (barc321;barc12 ) | Cuthbert, 2011 |
| StbWW | 1B | 53059799 |  | barc119, | Raman et al., 2009 |
| TmStb1 | 7A | 116002861 |  | (Xbarc174;) | Jing et al., 2008 |
| MQTL11 | 2D | 79926878 | 170364558 | (wPt-665644, wPt-0298) wPt-8330 | Goudemand et al, 2013 |
| MQTL13 | 3A | 721223988 | 743563796 | wPt-1036 (wPt-1596, wPt-9761) | Goudemand et al, 2013 |
| MQTL17 | 4B | 549138483 | 579103874 | wPt-1101 (wPt-7365, wPt-6209, wPt-3991) | Goudemand et al, 2013 |
| MQTL20 | 6A | 1252532 | 25798538 | tPt-4209 (wPt-1664, wPt-9132, wPt-0864) | Goudemand et al, 2013 |
| MQTL24 | 7A | 693198258 | 721774214 | (wPt-5524, wPt-0639, wPt-744897) wPt-0971 | Goudemand et al, 2013 |
| MQTL3 | 1B | 632911927 | 632912608 | (wPt-6975) wPt-4721 (wPt-5281) | Goudemand et al, 2013 |
| MQTL4 | 2A | 31987181 | 60709473 | (wPt-740658, wmc177, wPt-9320) wPt-6711 | Goudemand et al, 2013 |
| MQTL5 | 2A | 759323599 | 763485731 | (wPt-9277, wPt-6662, wPt-741584) wPt-7901 | Goudemand et al, 2013 |
| MQTL8 | 2B | 686046185 | 708210455 | (wPt-1646, wPt-0189) wPt-8340 | Goudemand et al, 2013 |
