# Supplementary material for: Identification of resistance sources and genomic regions regulating Septoria tritici blotch resistance in South Asian bread wheat germplasm
Source: Plant Genome. 2024 Nov 27;18(1):e20531. doi: 10.1002/tpg2.20531 (PMC11726422; doi:10.1002/tpg2.20531)
Supplement: Supplementary file 1 — Table S1 List of genotypes along with parentage and gBLUP values. Table S2 Statistical and genetic parameters for days to heading and plant height over the respective year and pool over years. Table S3 The unique quantitative trait nucleotides (QTNs) controlling the Septoria tritici blotch resistance. Table S4 The unique quantitative trait nucleotides (QTNs) controlling the plant height (PH) and days to heading (DH). Table S5 Unique quantitative trait nucleotides (QTNs) in complete linkage across the genome. Table S6 The physical position of reported Stb genes and meta‐QTLs. [file TPG2-18-e20531-s002.docx]

**Table S1:** List of genotypes along with parentage and gBLUP values

| **GID** | **TAXA** | **Parentage** | **STB2019** | **STB2020** | **STB2021** | **STB_POOL** | **2NS/2AS** |
| --- | --- | --- | --- | --- | --- | --- | --- |
| 7890088 | BGD1 | BIJOY | 1211.5 | 1142.4 | 891.5 | 1096.0 | 2AS |
| 7890112 | BGD10 | BAW1195 | 1263.9 | 1163.9 | 896.9 | 1138.6 | 2AS |
| 7890115 | BGD11 | BAW1200 | 1261.5 | 1109.3 | 831.5 | 1074.7 | 2AS |
| 7890118 | BGD12 | BAW1202 | 1301.9 | 1216.2 | 849.6 | 1157.0 | 2AS |
| 7890120 | BGD13 | BAW1203 | 1340.9 | 1206.9 | 844.3 | 1148.2 | 2AS |
| 7890122 | BGD14 | BAW1208 | 1173.3 | 1240.4 | 946.7 | 1164.8 | 2AS |
| 7890125 | BGD15 | BAW1209 | 1132.9 | 1196.2 | 855.4 | 1056.3 | 2AS |
| 7890128 | BGD16 | BAW1219 | 1292.3 | 1242.3 | 976.6 | 1218.9 | 2AS |
| 7890134 | BGD17 | BAW1222 | 1289.7 | 1177.7 | 841.0 | 1110.2 | 2AS |
| 7890135 | BGD18 | BAW1249 | 1458.7 | 1278.2 | 959.8 | 1279.6 | 2AS |
| 7890137 | BGD19 | BAW1260 | 1406.7 | 1019.3 | 888.7 | 1164.8 | 2NS |
| 7890090 | BGD2 | PRODIP | 1293.8 | 1225.3 | 924.1 | 1188.9 | 2AS |
| 7890092 | BGD3 | BARIGOM25 | 1066.7 | 866.3 | 805.8 | 871.9 | 2AS |
| 7890096 | BGD4 | BARIGOM26 | 1274.6 | 1236.7 | 912.5 | 1214.2 | 2AS |
| 7890101 | BGD5 | BARIGOM28 | 1013.8 | 1044.8 | 845.5 | 991.8 | 2AS |
| 7890103 | BGD6 | BARIGOM29 | 1220.7 | 1143.3 | 859.1 | 1091.6 | 2AS |
| 7890104 | BGD7 | BARIGOM30 | 1099.3 | 1158.2 | 760.1 | 990.9 | 2AS |
| 7890106 | BGD8 | BAW1182 | 1092.9 | 1089.8 | 814.3 | 989.5 | 2AS |
| 7890110 | BGD9 | BAW1194 | 1428.9 | 1209.5 | 935.9 | 1256.2 | 2AS |
| 6681676 | CIM1 | QUAIU#1/SUP152 | 1002.8 | 842.0 | 801.5 | 880.6 | 2AS |
| 6680982 | CIM10 | FRET2*2/SHAMA//KIRITATI/2*TRCH/3/BAJ#1 | 759.3 | 784.3 | 750.1 | 745.0 | 2AS |
| 6567313 | CIM11 | PAURAQ/5/KIRITATI/4/2*SERI.1B*2/3/KAUZ*2/BOW//KAUZ/6/PAURAQUE#1 | 826.2 | 766.1 | 801.7 | 806.4 | 2AS |
| 6692346 | CIM12 | SOKOLL/3/PASTOR//HXL7573/2*BAU/5/CROC_1/AE.SQUARROSA(205)//BORL95/3/PRL/SARA//TSI/VEE#5/4/FRET2 | 756.7 | 655.1 | 669.9 | 674.1 | 2AS |
| 6679711 | CIM13 | KENYASUNBIRD/2*KACHU | 1124.1 | 905.6 | 859.2 | 960.2 | 2NS |
| 6690981 | CIM15 | TOB/ERA//TOB/CNO67/3/PLO/4/VEE#5/5/KAUZ/6/FRET2/7/VORB/8/MILAN/KAUZ//DHARWARDRY/3/BAV92 | 634.4 | 726.1 | 713.4 | 671.0 | 2NS |
| 6566828 | CIM16 | KIRITATI//2*PRL/2*PASTOR/3/CHONTE/5/PRL/2*PASTOR/4/CHOIX/STAR/3/HE1/3*CNO79//2*SERI | 714.7 | 800.6 | 826.3 | 798.0 | 2AS |
| 6566596 | CIM17 | FRET2*2/BRAMBLING//KIRITATI/2*TRCH/3/FRET2/TUKURU//FRET2 | 880.9 | 856.6 | 825.9 | 883.2 | 2AS |
| 6681164 | CIM18 | KIRITATI//HUW234+LR34/PRINIA/3/CHONTE/5/PRL/2*PASTOR/4/CHOIX/STAR/3/HE1/3*CNO79//2*SERI | 615.4 | 641.0 | 727.5 | 625.6 | 2AS |
| 6568165 | CIM19 | SUP152/FRNCLN | 801.9 | 728.5 | 740.3 | 724.6 | 2AS |
| 6683477 | CIM2 | KIRITATI//HUW234+LR34/PRINIA/3/BAJ#1 | 675.3 | 743.0 | 752.5 | 736.5 | 2NS |
| 6691721 | CIM20 | BAVIS/3/ATTILA/BAV92//PASTOR/5/CROC_1/AE.SQUARROSA(205)//BORL95/3/PRL/SARA//TSI/VEE#5/4/FRET2 | 347.7 | 397.5 | 563.6 | 339.9 | 2AS |
| 6681777 | CIM21 | ND643/2*WBLL1//VILLAJUAREZF2009 | 989.6 | 888.4 | 834.5 | 913.2 | 2AS |
| 6085788 | CIM22 | QUAIU#1 | 1000.0 | 770.3 | 777.2 | 858.6 | 2AS |
| 6567425 | CIM23 | FRANCOLIN#1/CHONTE//FRNCLN | 984.5 | 799.3 | 833.7 | 898.3 | 2AS |
| 6678574 | CIM24 | TAM200/PASTOR//TOBA97/3/FRNCLN/4/WHEAR//2*PRL/2*PASTOR | 682.3 | 696.8 | 759.2 | 698.8 | 2AS |
| 5398434 | CIM25 | FRANCOLIN#1 | 851.6 | 786.0 | 780.3 | 804.7 | 2AS |
| 6691523 | CIM26 | VEE/MJI//2*TUI/3/PASTOR/4/BERKUT/5/BAVIS | 439.9 | 551.5 | 641.7 | 459.3 | 2AS |
| 6566821 | CIM27 | DANPHE/PAURAQUE#1//MUNAL#1 | 952.7 | 899.4 | 828.0 | 914.5 | 2AS |
| 6681493 | CIM28 | BAJ#1*2/KISKADEE#1 | 994.2 | 909.0 | 849.4 | 944.1 | 2AS |
| 4747362 | CIM29 | SHATABDI | 1091.1 | 941.6 | 793.8 | 932.5 | 2AS |
| 6681464 | CIM3 | FRANCOLIN#1*2//ND643/2*WBLL1 | 941.7 | 847.7 | 821.2 | 898.3 | 2AS |
| 6001093 | CIM30 | SOKOLL/ROLF07 | 835.3 | 683.2 | 737.0 | 716.3 | 2AS |
| 6001439 | CIM31 | ATTILA/BAV92//PASTOR/3/ATTILA*2/PBW65 | 830.3 | 745.4 | 732.0 | 733.0 | 2AS |
| 6564531 | CIM32 | WHEAR/KUKUNA/3/C80.1/3*BATAVIA//2*WBLL1/4/T.DICOCCONPI94625/AE.SQUARROSA(372)//SHA4/CHIL/5/WHEAR/KUKUNA/3/C80.1/3*BATAVIA//2*WBLL1 | 775.3 | 626.8 | 669.5 | 651.4 | 2AS |
| 27001 | CIM33 | TEPOCAT89 | 1003.7 | 1099.3 | 818.0 | 983.9 | 2AS |
| 12725 | CIM34 | MILAN | 641.4 | 632.4 | 695.0 | 611.0 | 2NS |
| 7806808 | CIM35 | BORLAUG100F2014 | 994.4 | 838.0 | 766.0 | 870.7 | 2NS |
| 4905617 | CIM36 | ROELFSF2007 | 971.9 | 807.3 | 808.5 | 910.8 | 2AS |
| 6333158 | CIM37 | SUP152/BAJ#1 | 941.5 | 1023.8 | 783.8 | 911.5 | 2AS |
| 14337 | CIM38 | ATTILA | 977.6 | 985.2 | 807.9 | 966.4 | 2AS |
| 3855011 | CIM39 | VOROBEY | 587.3 | 859.1 | 734.8 | 686.6 | 2AS |
| 6692031 | CIM4 | CROC_1/AE.SQUARROSA(213)//PGO/10/ATTILA*2/9/KT/BAGE//FN/U/3/BZA/4/TRM/5/ALDAN/6/SERI/7/VEE#10/8/OPATA/11/ATTILA*2/PBW65 | 606.4 | 690.9 | 735.4 | 644.0 | 2AS |
| 1396784 | CIM40 | MILAN/MUNIA | 792.1 | 668.7 | 673.9 | 705.7 | 2NS |
| 5398434 | CIM41 | FRANCOLIN#1 | 905.4 | 804.5 | 792.5 | 833.2 | 2AS |
| 5390612 | CIM42 | SUPER152 | 883.2 | 957.3 | 813.5 | 923.6 | 2AS |
| 4755014 | CIM43 | KACHU#1 | 1330.0 | 1023.9 | 939.0 | 1156.3 | 2NS |
| 4754390 | CIM44 | MUTUS#1 | 690.8 | 644.1 | 706.2 | 659.5 | 2NS |
| 5794351 | CIM45 | AKEPA | 751.0 | 847.4 | 787.0 | 811.6 | 2AS |
| 6174889 | CIM46 | BOKOTA | 807.9 | 740.3 | 788.3 | 766.4 | 2NS |
| 5106304 | CIM47 | BAJ#1 | 947.8 | 968.8 | 811.8 | 927.1 | 2AS |
| 6177828 | CIM48 | FRNCLN/ROLF07 | 1014.1 | 906.5 | 864.1 | 948.7 | 2AS |
| 6175067 | CIM49 | NADI#2 | 1069.4 | 968.6 | 868.8 | 989.1 | 2NS |
| 6682922 | CIM5 | BAJ#1*2//ND643/2*WBLL1 | 1095.7 | 932.1 | 887.1 | 1008.6 | 2AS |
| 6337034 | CIM50 | FRNCLN*2/TECUE#1 | 870.6 | 718.4 | 799.4 | 789.7 | 2NS |
| 6341870 | CIM51 | MUCUY | 824.9 | 652.9 | 716.5 | 700.6 | 2NS |
| 6464471 | CIM52 | KACHU/BECARD//WBLL1*2/BRAMBLING | 1003.9 | 858.7 | 827.1 | 932.2 | 2AS |
| 6415882 | CIM53 | KIDEA | 867.8 | 780.0 | 751.6 | 772.1 | 2AS |
| 6681676 | CIM54 | QUAIU#1/SUP152 | 949.6 | 846.6 | 795.7 | 875.6 | 2AS |
| 6568165 | CIM55 | SUP152/FRNCLN | 805.7 | 746.3 | 741.3 | 740.5 | 2AS |
| 6682903 | CIM56 | FRNCLN/3/ND643//2*PRL/2*PASTOR/4/FRANCOLIN#1 | 510.0 | 511.8 | 661.1 | 477.5 | 2AS |
| 6684333 | CIM57 | SWSR22T.B./2*BLOUK#1//WBLL1*2/KURUKU | 560.8 | 600.3 | 682.7 | 559.0 | 2NS |
| 6679899 | CIM58 | FRANCOLIN#1*2//ND643/2*WBLL1 | 869.9 | 817.3 | 798.9 | 822.3 | 2AS |
| 6932283 | CIM59 | DANPHE/2*BAJ#1 | 831.5 | 821.5 | 773.1 | 771.3 | 2AS |
| 6679852 | CIM6 | PAURAQ/4/WHEAR/KUKUNA/3/C80.1/3*BATAVIA//2*WBLL1/5/PAURAQUE#1 | 1085.7 | 1005.5 | 808.4 | 989.6 | 2AS |
| 6932418 | CIM60 | BAJ#1*2/BECARD | 916.0 | 809.0 | 750.0 | 775.2 | 2AS |
| 6932429 | CIM61 | BAJ#1*2/5/SW89.5277/BORL95//SKAUZ/3/PRL/2*PASTOR/4/HEILO | 917.4 | 900.6 | 816.3 | 898.9 | 2AS |
| 6931516 | CIM62 | FRANCOLIN#1/BAJ#1 | 960.8 | 903.7 | 833.6 | 901.6 | 2AS |
| 6933207 | CIM63 | BAJ#1/CIRO16 | 1076.2 | 1019.0 | 858.7 | 1027.4 | 2NS |
| 6934570 | CIM64 | PBW343*2/KUKUNA//PBW343*2/KUKUNA/3/2*BAJ#1 | 994.1 | 917.1 | 794.4 | 882.9 | 2AS |
| 7175990 | CIM65 | BAJ#1/5/ATTILA/3*BCN//BAV92/3/TILHI/4/SHA7/VEE#5//ARIV92 | 1046.7 | 960.1 | 816.7 | 965.8 | 2NS |
| 7175993 | CIM66 | BAJ#1/CIRO16 | 966.2 | 877.4 | 821.2 | 883.3 | 2NS |
| 7176068 | CIM67 | SUP152/3/INQALAB91*2/TUKURU//WHEAR | 1039.7 | 915.5 | 871.7 | 964.9 | 2NS |
| 7176119 | CIM68 | SUP152/CIRO16 | 955.4 | 900.8 | 873.9 | 930.2 | 2NS |
| 7398595 | CIM69 | ATTILA*2/PBW65//KACHU/3/UP2338*2/KKTS*2//YANAC | 887.0 | 820.5 | 831.6 | 847.5 | 2NS |
| 6464471 | CIM7 | KACHU/BECARD//WBLL1*2/BRAMBLING | 1007.3 | 831.7 | 822.5 | 888.4 | 2AS |
| 7396039 | CIM70 | KASUKO | 1016.0 | 840.6 | 764.9 | 855.9 | 2NS |
| 7396072 | CIM71 | KFA/2*KACHU/3/ATTILA*2/PBW65//MURGA | 844.4 | 762.3 | 831.6 | 824.0 | 2NS |
| 7396125 | CIM72 | ATTILA*2/PBW65*2//KACHU/3/TRCH/HUIRIVIS#1 | 873.6 | 799.7 | 826.5 | 848.0 | 2NS |
| 7396133 | CIM73 | BORL14//KFA/2*KACHU | 1032.2 | 862.4 | 794.0 | 879.2 | 2NS |
| 7396135 | CIM74 | BORL14//KFA/2*KACHU | 977.8 | 815.5 | 794.3 | 892.1 | 2NS |
| 7396139 | CIM75 | BORL14//KFA/2*KACHU | 1101.2 | 744.0 | 750.7 | 855.2 | 2NS |
| 7396142 | CIM76 | BORL14//KFA/2*KACHU | 985.9 | 741.2 | 759.0 | 810.1 | 2NS |
| 7310905 | CIM77 | KACHU/KIRITATI//BORL14 | 775.2 | 765.1 | 769.5 | 743.4 | 2NS |
| 7400913 | CIM78 | KASUKO | 1152.0 | 889.7 | 815.6 | 956.7 | 2NS |
| 7400914 | CIM79 | KASUKO | 1100.3 | 901.5 | 821.6 | 953.2 | 2NS |
| 6679708 | CIM8 | MUTUS//KIRITATI/2*TRCH/3/WHEAR/KRONSTADF2004 | 1166.7 | 972.3 | 895.7 | 1024.0 | 2NS |
| 7400915 | CIM80 | KASUKO | 957.8 | 904.6 | 796.0 | 893.1 | 2NS |
| 7400916 | CIM81 | KASUKO | 956.6 | 819.8 | 745.8 | 835.2 | 2NS |
| 4836044 | CIM82 | ORL93320/ER2000 | 606.8 | 593.5 | 667.4 | 548.4 | 2NS |
| 4911209 | CIM83 | SW91.4903/3/URES/BOW//OPATA/4/SW94.15373 | 586.7 | 590.7 | 713.7 | 584.2 | 2AS |
| 4940678 | CIM84 | MON/TAN//ROMO96/3/METSO/4/FINSI | 535.4 | 627.7 | 608.3 | 545.6 | 2NS |
| 5090596 | CIM85 | MUU | 726.2 | 657.5 | 767.4 | 701.8 | 2AS |
| 5090672 | CIM86 | PFAU/WEAVER*2/3/WEAVER/ESDA//BORL95 | 734.2 | 801.3 | 797.3 | 776.8 | 2AS |
| 5106391 | CIM87 | SW8488*2/KURUKU | 874.2 | 918.2 | 718.8 | 839.0 | 2AS |
| 4936520 | CIM88 | FINSI/METSO | 462.3 | 461.4 | 574.0 | 461.8 | 2NS |
| 4936526 | CIM89 | FINSI/METSO | 474.8 | 496.9 | 574.0 | 472.2 | 2NS |
| 6569147 | CIM9 | BAJ#1*2/TINKIO#1 | 1122.7 | 959.1 | 888.0 | 1024.4 | 2AS |
| 4936533 | CIM90 | FINSI/METSO | 400.5 | 455.7 | 550.1 | 412.2 | 2NS |
| 4935134 | CIM91 | BAV92//IRENA/KAUZ/3/HUITES | 818.2 | 827.9 | 743.2 | 763.7 | 2AS |
| 4885822 | CIM92 | GONDO//BAU/MILAN/3/PASTOR | 592.1 | 662.2 | 724.9 | 632.7 | 2AS |
| 4756015 | CIM93 | ATTILA/3*BCN//BAV92/3/TILHI | 868.8 | 885.5 | 740.9 | 817.2 | 2NS |
| 4755014 | CIM94 | KACHU#1 | 1301.2 | 1029.5 | 920.5 | 1110.0 | 2NS |
| 4944857 | CIM95 | TEG/MIANYANG20//CHUM18/5*BCN | 823.1 | 749.9 | 687.7 | 704.7 | 2AS |
| 4937343 | CIM96 | JWS17/7/IAS58/4/KAL/BB//CJ71/3/ALD/5/CNR/6/THB/CEP7780/8/FINSI | 463.0 | 572.7 | 610.4 | 487.5 | 2NS |
| 4960851 | CIM97 | MILAN/ARA90//TNMU/TUI | 754.7 | 760.6 | 704.2 | 726.5 | 2NS |
| 7025946 | IND1 | HD2967 | 781.2 | 716.0 | 771.1 | 762.3 | 2NS |
| 6927036 | IND10 | WH1105 | 694.2 | 608.5 | 705.3 | 667.4 | 2NS |
| 7895088 | IND11 | WH1124 | 927.4 | 813.2 | 829.9 | 840.3 | 2AS |
| 7895089 | IND12 | WH1142 | 916.6 | 896.7 | 798.4 | 891.8 | 2AS |
| 7895090 | IND13 | HD3171 | 770.4 | 765.6 | 750.2 | 722.9 | 2NS |
| 7895091 | IND14 | K1317 | 395.2 | 554.0 | 653.9 | 450.7 | 2AS |
| 3575624 | IND15 | C306 | 1087.4 | 917.6 | 732.8 | 905.4 | 2AS |
| 7025950 | IND16 | DBW39 | 965.2 | 934.9 | 771.3 | 900.2 | 2NS |
| 4048863 | IND17 | HD2733 | 866.2 | 962.6 | 718.9 | 811.3 | 2AS |
| 7025907 | IND18 | HD2888 | 1143.3 | 945.2 | 741.1 | 936.6 | 2AS |
| 152830 | IND19 | MAGHAR | 978.2 | 796.9 | 764.8 | 815.2 | 2AS |
| 7895083 | IND2 | DBW90 | 855.4 | 868.2 | 828.1 | 879.0 | 2AS |
| 7025921 | IND20 | K0307 | 974.1 | 891.4 | 793.7 | 888.4 | 2AS |
| 7522443 | IND21 | K1006 | 945.1 | 887.3 | 789.9 | 853.4 | 2AS |
| 7895097 | IND28 | HI1605 | 646.5 | 597.2 | 675.3 | 580.3 | 2NS |
| 7895084 | IND3 | HD3043 | 709.5 | 649.3 | 644.5 | 622.1 | 2NS |
| 7895098 | IND30 | DBW93 | 737.2 | 723.1 | 746.7 | 693.5 | 2AS |
| 7025931 | IND31 | HD2932 | 1410.4 | 1093.4 | 824.8 | 1144.5 | 2AS |
| 7025955 | IND32 | MACS6222 | 981.5 | 1043.9 | 839.9 | 981.3 | 2AS |
| 7895099 | IND33 | MACS6478 | 1252.8 | 1051.3 | 816.1 | 1080.4 | 2NS |
| 23028 | IND34 | NI5439 | 1178.3 | 1132.6 | 861.2 | 1112.9 | 2AS |
| 7895100 | IND35 | NIAW1415 | 955.2 | 860.8 | 749.3 | 838.6 | 2AS |
| 7895101 | IND36 | NIAW34 | 921.2 | 880.9 | 854.2 | 900.2 | 2AS |
| 7025915 | IND38 | RAJ4083 | 1214.0 | 1003.7 | 838.3 | 1046.6 | 2AS |
| 7895085 | IND4 | DBW88 | 1284.4 | 1024.9 | 934.2 | 1105.5 | 2NS |
| 6927038 | IND5 | HD3059 | 1315.9 | 1020.5 | 936.2 | 1135.9 | 2NS |
| 7895086 | IND6 | HD3086 | 826.2 | 901.8 | 841.6 | 915.6 | 2AS |
| 7895087 | IND7 | PBW644 | 1008.3 | 794.0 | 796.9 | 875.3 | 2AS |
| 7025929 | IND8 | WH1021 | 1043.5 | 1109.2 | 787.4 | 988.4 | 2AS |
| 7025960 | IND9 | WH1080 | 690.0 | 810.3 | 802.0 | 802.0 | 2AS |
| 264591 | NPL1 | BHRIKUTI | 1088.6 | 1098.6 | 923.5 | 1068.2 | 2AS |
| 7895108 | NPL10 | BL4407 | 1116.2 | 923.6 | 906.8 | 1011.8 | 2AS |
| 7895109 | NPL11 | BL4725 | 867.5 | 902.2 | 761.0 | 811.5 | 2AS |
| 7895110 | NPL12 | BL4707 | 985.7 | 864.1 | 849.6 | 908.1 | 2AS |
| 7895111 | NPL13 | BL4406 | 1111.4 | 941.9 | 910.2 | 999.0 | 2AS |
| 7895112 | NPL14 | BL4682 | 979.8 | 823.9 | 774.8 | 858.7 | 2AS |
| 7895113 | NPL15 | BL4621 | 1141.9 | 1176.0 | 938.9 | 1130.2 | 2AS |
| 7895114 | NPL16 | BL4622 | 1056.2 | 1144.1 | 901.7 | 1069.8 | 2AS |
| 7895115 | NPL17 | NL1164 | 887.8 | 937.0 | 793.6 | 876.8 | 2NS |
| 7895116 | NPL18 | NL1202 | 1112.1 | 999.2 | 894.6 | 1050.5 | 2NS |
| 7895117 | NPL19 | NL1249 | 731.4 | 736.5 | 787.4 | 730.0 | 2AS |
| 5956817 | NPL2 | GAUTAM | 996.2 | 847.8 | 808.9 | 878.1 | 2AS |
| 7895118 | NPL20 | NL1270 | 550.9 | 746.2 | 730.9 | 602.7 | 2NS |
| 7895119 | NPL21 | NL1178 | 658.6 | 772.7 | 722.4 | 703.1 | 2AS |
| 7895120 | NPL22 | NL1226 | 512.6 | 621.8 | 712.3 | 598.1 | 2AS |
| 7895121 | NPL23 | NL1231 | 1012.1 | 827.7 | 769.7 | 894.0 | 2AS |
| 7895122 | NPL24 | WK2432 | 792.9 | 946.1 | 791.7 | 848.8 | 2AS |
| 7895123 | NPL25 | WK2375 | 895.7 | 776.6 | 776.0 | 800.1 | 2AS |
| 7895124 | NPL26 | WK2123 | 1158.9 | 986.8 | 888.7 | 1074.8 | 2AS |
| 7895125 | NPL27 | WK2286 | 1020.9 | 712.2 | 742.3 | 790.7 | 2AS |
| 7895126 | NPL28 | WK2395 | 771.2 | 708.5 | 701.2 | 664.9 | 2AS |
| 7895104 | NPL3 | WK1204 | 1063.8 | 775.9 | 789.8 | 876.1 | 2AS |
| 6561770 | NPL4 | ADITYA | 1233.0 | 795.5 | 838.4 | 986.4 | 2AS |
| 5218199 | NPL5 | VIJAY | 922.3 | 929.5 | 923.2 | 959.7 | 2AS |
| 7895105 | NPL6 | DHAWALAGIRI | 1270.8 | 1007.9 | 819.3 | 1052.3 | 2AS |
| 6308951 | NPL7 | BL3623 | 1145.4 | 948.3 | 826.1 | 993.0 | 2AS |
| 7895106 | NPL8 | BL3629 | 1199.9 | 1027.0 | 848.9 | 1053.6 | 2AS |
| 7895107 | NPL9 | BL4341 | 1185.3 | 904.6 | 946.0 | 1060.6 | 2AS |

**Table S2:** Statistical and genetic parameters for Days to heading and plant height over the respective year and pool over years

| **Statistical parameters** | **DH 2019** | **DH 2020** | **DH 2021** | **DH_POOL** | **PH 2019** | **PH 2020** | **PH 2021** | **PH_POOL** |
| --- | --- | --- | --- | --- | --- | --- | --- | --- |
| Heritability | 0.95 | 0.91 | 0.97 | 0.86 | 0.95 | 0.97 | 0.92 | 0.74 |
| Genotype Variance | 14.20 | 11.70 | 17.90 | 10.44 | 75.26 | 52.53 | 52.04 | 30.85 |
| Genotype × Year Variance |  |  |  | 4.15 |  |  |  | 29.09 |
| Year variance |  |  |  | 5.69 |  |  |  | 46.28 |
| Residual variance | 1.53 | 2.37 | 1.09 | 1.66 | 8.50 | 3.73 | 9.52 | 7.24 |
| Grand Mean | 67.53 | 64.44 | 69.15 | 67.04 | 94.75 | 103.09 | 108.27 | 102.04 |
| LSD | 2.38 | 2.90 | 2.03 | 3.39 | 5.60 | 3.75 | 5.83 | 7.96 |
| CV | 1.83 | 2.39 | 1.51 | 1.92 | 3.08 | 1.87 | 2.85 | 2.64 |
| Genotype significance | 0 | 0 | 0 | 1.88E-54 | 0 | 0 | 0 | 3.9204E-26 |
| Genotype × Year significance |  |  |  | 2.84E-73 |  |  |  | 8.30E-107 |
| Year significance |  |  |  | 0.000686 |  |  |  | 1.27592E-05 |

**Table S3:** The unique quantitative trait nucleotides (QTNs) controlling the Septoria tritici blotch resistance

| **QTNs** | **SNP** | **Chr** | **Pos** | **Effect** | **LOG P VALUE** | **Percent Variation** | **Year** | **Gene ID** | **Position** | **Description with Prot ID** | **Biological function** |
| --- | --- | --- | --- | --- | --- | --- | --- | --- | --- | --- | --- |
| Q.CIM.stb.1AL.6 | GENE-0115_375 | 1A | 5.75E+08 | 81.30624 | 3.710828 |  | BLINKSTB2020 | TraesCS1A02G416500 | chr1A:574826362-574832131(-) | NA |  |
| Q.CIM.stb.1AL.7 | Kukri_c30490_328 | 1A | 5.75E+08 | -81.1384 | 3.785564 |  | BLINKSTB2020 | TraesCS1A02G416600 | chr1A:574933843-574935896(+) | Transcription elongation factor 1 homolog [UniProtKB/Swiss-Prot:Q8LHP0] | transcription elongation from RNA polymerase II promoter |
| Q.CIM.stb.1AL.8 | RAC875_c55934_165 | 1A | 5.75E+08 | -78.5204 | 3.505254 |  | BLINKSTB2020 | TraesCS1A02G416600 | chr1A:574933843-574935896(+) | Transcription elongation factor 1 homolog [UniProtKB/Swiss-Prot:Q8LHP0] | transcription elongation from RNA polymerase II promoter |
| Q.CIM.stb.1AS.2 | Tdurum_contig9199_714 | 1A | 45796934 | -55.9255 | 5.963495 | 1.354226 | FarmCPUSTB2019 | TraesCS1A02G064100 | chr1A:45793606-45797826(+) | Protein IQ-DOMAIN 1 [UniProtKB/Swiss-Prot:Q9SF32] |  |
| Q.CIM.stb.1BL.3 | Kukri_c30461_857 | 1B | 6.15E+08 | 23.78835 | 5.660014 | 1.322307 | FarmCPUSTB2021 | TraesCS1B02G382600 | chr1B:614788759-614794654(+) | Phospholipase D beta 1 [UniProtKB/Swiss-Prot:P93733] | phosphatidylcholine metabolic process |
|  |  |  |  |  |  |  |  | TraesCS1B02G379700 | chr1B:612893037-612894939(+) | Probable cytokinin riboside 5'-monophosphate phosphoribohydrolase LOGL7 [UniProtKB/Swiss-Prot:Q5TKP8] | cytokinin biosynthetic process [GO:0009691] |
|  |  |  |  |  |  |  |  | TraesCS1B02G383300 | chr1B:615117570-615125086(+) | Protein ANTHESIS POMOTING FACTOR 1 [UniProtKB/Swiss-Prot:Q9LYK6] | histone H3-K4 trimethylation [GO:0080182] |
|  |  |  |  |  |  |  |  | TraesCS1B02G380000 | chr1B:613020322-613022195(+) | Transcription factor LATE FLOWERING [UniProtKB/Swiss-Prot:A0A0P0WQ90] | regulation of long-day photoperiodism, flowering [GO:0048586] |
| Q.CIM.stb.1BS.2 | Ra_c4899_609 | 1B | 2.34E+08 | 79.80508 | 3.145206 |  | MLMSTB2020 PC6 | TraesCS1B02G150900 | chr1B:234251618-234254193(-) | Ras-related protein RGP2 [UniProtKB/Swiss-Prot:Q40723] |  |
| Q.CIM.stb.1DS.1 | D_contig13475_402 | 1D | 20045042 | 88.93567 | 3.153394 |  | MLMSTB2020 PC6 | TraesCS5A02G396500 | chr5A:591313846-591320402(+) | NA |  |
| Q.CIM.stb.2AL.4 | RAC875_c19328_236 | 2A | 4.01E+08 | -99.8754 | 3.421572 |  | MLMSTB2019 PC6 | NA | #N/A | #N/A |  |
| Q.CIM.stb.2AL.6 | wsnp_CAP7_c1592_791887 | 2A | 7.31E+08 | 128 | 7.091825 | 7.559225 | BLINKSTB2021 | TraesCS2A02G502400 | chr2A:731156504-731163306(-) | Plasma membrane ATPase [UniProtKB/Swiss-Prot:P83970] | ion transmembrane transport, regulation of intracellular pH |
|  |  |  |  |  |  |  |  | TraesCS2A02G501900 | chr2A:729852393-729858636(+) | Farnesyl pyrophosphate synthase [UniProtKB/Swiss-Prot:Q4JHN6] | farnesyl diphosphate biosynthetic process [GO:0045337] |
|  |  |  |  |  |  |  |  | TraesCS2A02G501000 | chr2A:729576096-729579455(+) | Ribosomal RNA processing protein 1 homolog B [UniProtKB/Swiss-Prot:Q14684] | negative regulation of GTPase activity [GO:0034260] |
|  |  |  |  |  |  |  |  | TraesCS2A02G501300 | chr2A:729687927-729689703(+) | GTPase activating protein 1 [UniProtKB/Swiss-Prot:Q6YWF1] | positive regulation of defense response to bacterium, incompatible interaction [GO:1902479] |
|  |  |  |  |  |  |  |  | TraesCS2A02G502400 | chr2A:731156504-731163306(-) | Plasma membrane ATPase [UniProtKB/Swiss-Prot:P83970] | proton export across plasma membrane [GO:0120029] |
| Q.CIM.stb.2AL.7 | BS00082084_51 | 2A | 7.33E+08 | 93.23496 | 7.806079 | 9.861082 | FarmCPUSTB2020 | TraesCS2A02G503000 | chr2A:732569049-732570858(-) | NA | defense response, negative regulation of translation |
|  |  |  |  |  |  |  |  | TraesCS2A02G504400 | chr2A:733287115-733289358(-) | Subtilisin-like protease 4 [UniProtKB/Swiss-Prot:A9QY38] | arbuscular mycorrhizal association [GO:0036377] |
|  |  |  |  |  |  |  |  | TraesCS2A02G505000 | chr2A:733560806-733563422(+) | Sucrose transport protein SUT5 [UniProtKB/Swiss-Prot:A2X6E6] | #N/A |
|  |  |  |  |  |  |  |  | TraesCS2A02G506200 | chr2A:734148608-734153261(-) | Vacuolar cation/proton exchanger 3 [UniProtKB/Swiss-Prot:Q6K1C4] | cellular calcium ion homeostasis [GO:0006874] |
|  |  |  |  |  |  |  |  | TraesCS2A02G504600 | chr2A:733394610-733398877(-) | COP9 signalosome complex subunit 5 [UniProtKB/Swiss-Prot:Q8H936] | #N/A |
|  |  |  |  |  |  |  |  | TraesCS2A02G503100 | chr2A:732580211-732581104(+) | Ethylene-responsive transcription factor ERF003 [UniProtKB/Swiss-Prot:Q94AW5] | ethylene-activated signaling pathway [GO:0009873] |
|  |  |  |  |  |  |  |  | TraesCS2A02G505800 | chr2A:733921860-733929336(-) | NADPH--cytochrome P450 reductase [UniProtKB/Swiss-Prot:Q05001] | #N/A |
| Q.CIM.stb.2AS.1 | RAC875_c21868_792 | 2A | 30452856 | -42.695 | 5.211511 | 0.388925 | FarmCPUSTB2020 | TraesCS2A02G069400 | chr2A:30451134-30464943(-) | Acetyl-CoA carboxylase 2 [UniProtKB/Swiss-Prot:B9FK36] |  |
|  |  |  |  |  |  |  |  | TraesCS2A02G069900 | chr2A:30764411-30766386(+) | 2-alkenal reductase (NADP(+)-dependent) [UniProtKB/Swiss-Prot:Q9SLN8] | #N/A |
|  |  |  |  |  |  |  |  | TraesCS2A02G069400 | chr2A:30451134-30464943(-) | Acetyl-CoA carboxylase 2 [UniProtKB/Swiss-Prot:B9FK36] | malonyl-CoA biosynthetic process [GO:2001295] |
|  |  |  |  |  |  |  |  | TraesCS2A02G071000 | chr2A:31648562-31686564(-) | Arginine decarboxylase [UniProtKB/Swiss-Prot:P22220] | arginine catabolic process [GO:0006527] |
|  |  |  |  |  |  |  |  | TraesCS2A02G066400 | chr2A:29933733-29935186(+) | Probable cinnamyl alcohol dehydrogenase 6 [UniProtKB/Swiss-Prot:Q7XWU3] | lignin biosynthetic process [GO:0009809] |
|  |  |  |  |  |  |  |  | TraesCS2A02G072100 | chr2A:32026318-32028546(-) | Two-component response regulator ORR42 [UniProtKB/Swiss-Prot:Q7XN30] | cytokinin-activated signaling pathway [GO:0009736] |
|  |  |  |  |  |  |  |  | TraesCS2A02G068400 | chr2A:30197044-30199142(-) | LRR receptor-like serine/threonine-protein kinase FLS2 [UniProtKB/Swiss-Prot:Q9FL28] | detection of bacterium [GO:0016045] |
|  |  |  |  |  |  |  |  | TraesCS2A02G069100 | chr2A:30305253-30308602(-) | LRR receptor-like serine/threonine-protein kinase EFR [UniProtKB/Swiss-Prot:C0LGT6] | innate immune response-activating signal transduction [GO:0002758] |
|  |  |  |  |  |  |  |  | TraesCS2A02G066700 | chr2A:30020633-30021500(-) | Ribulose bisphosphate carboxylase small chain PWS4.3, chloroplastic [UniProtKB/Swiss-Prot:P00871] | photorespiration [GO:0009853] |
| Q.CIM.stb.2AS.2 | AX-94639471 | 2A | 52129997 | -160.887 | 3.589311 |  | BLINKSTB ACROSS YEARS | TraesCS2A02G099200 | chr2A:52126850-52131904(+) | NA |  |
| Q.CIM.stb.2BL.4 | BS00047070_51 | 2B | 7.15E+08 | 464 | 3.936493 |  | BLINKSTB2020 | TraesCS2B02G520100 | chr2B:714779662-714782762(-) | U11/U12 small nuclear ribonucleoprotein 25 kDa protein [UniProtKB/Swiss-Prot:Q84WS8] | mRNA splicing, via spliceosome |
| Q.CIM.stb.2BL.5 | BS00065327_51 | 2B | 7.31E+08 | 41.74597 | 3.201556 |  | MLMSTB2021 PC6 | TraesCS2B02G535600 | chr2B:731480831-731482520(-) | 3-ketoacyl-CoA synthase 6 [UniProtKB/Swiss-Prot:Q9XF43] | fatty acid biosynthetic process |
| Q.CIM.stb.2BL.7 | RAC875_c50422_299 | 2B | 7.34E+08 | 39.25921 | 3.074288 |  | MLMSTB2021 PC6 | TraesCS2B02G538100 | chr2B:733642201-733647003(-) | Probable inactive receptor kinase RLK902 [UniProtKB/Swiss-Prot:Q9LVI6] | protein phosphorylation and cell surface receptor signaling pathway |
| Q.CIM.stb.2BL.8 | AX-94945263 | 2B | 7.49E+08 | -40.2838 | 3.085764 |  | MLMSTB2021 PC6 | TraesCS2B02G553700 | chr2B:749043905-749044536(-) | NA |  |
| Q.CIM.stb.2BS.1 | BS00010055_51 | 2B | 72610501 | 57.93331 | 4.087254 |  | FarmCPUSTB2019 | TraesCS2B02G110800 | chr2B:72608545-72619945(-) | Lon protease homolog, mitochondrial [UniProtKB/Swiss-Prot:Q69UZ3] | cellular response to oxidative stress |
| Q.CIM.stb.2BS.2 | BobWhite_c892_73 | 2B | 2.12E+08 | -5120 | 3.75108 |  | BLINKSTB2021 | TraesCS2B02G221600 | chr2B:211525650-211535423(+) | Importin-5 [UniProtKB/Swiss-Prot:Q8BKC5] | intracellular protein transport |
| Q.CIM.stb.2DS.1 | GENE-0581_156 | 2D | 49881735 | 384 | 4.831081 | 8.595425 | BLINKSTB ACROSS YEARS | TraesCS2D02G097700 | chr2D:49879249-49884545(-) | CBL-interacting protein kinase 31 [UniProtKB/Swiss-Prot:Q6X4A2] | protein phosphorylation;intracellular signal transduction |
| Q.CIM.stb.3AL.7 | RFL_Contig2394_439 | 3A | 7.49E+08 | 49.40193 | 4.160511 |  | FarmCPUSTB2020 | TraesCS3A02G537600 | chr3A:749276696-749281113(+) | DnaJ protein P58IPK homolog B [UniProtKB/Swiss-Prot:Q5JNB5] |  |
| Q.CIM.stb.3AS.1 | RAC875_c45016_79 | 3A | 53837058 | -24.9983 | 4.07456 |  | FarmCPUSTB2021 | TraesCS3A02G083800 | chr3A:53838186-53842020(-) | NA |  |
| Q.CIM.stb.3BL.4 | wsnp_RFL_Contig3396_3515504 | 3B | 5.41E+08 | -104.6 | 3.073965 |  | MLMSTB2020 PC6 | TraesCS3B02G335000 | chr3B:541080846-541085261(-) | Clp protease adapter protein ClpF, chloroplastic [UniProtKB/Swiss-Prot:Q67Y99] | chloroplastic endopeptidase Clp complex |
| Q.CIM.stb.3BL.5 | wsnp_CAP12_c2297_1121142 | 3B | 7.83E+08 | 33.7796 | 4.287921 |  | FarmCPUSTB2020 | TraesCS3B02G547900 | chr3B:783465998-783469436(-) | Uridine 5'-monophosphate synthase [UniProtKB/Swiss-Prot:Q9LDN2] | UMP biosynthetic process, pyrimidine nucleotide biosynthetic process, metabolic process |
| Q.CIM.stb.3BL.6 | AX-94437380 | 3B | 8.15E+08 | -54.3757 | 5.087521 | 0.338167 | FarmCPUSTB2020 | TraesCS3B02G589300 | chr3B:814795775-814800995(-) | Disease resistance protein Piks-2 [UniProtKB/Swiss-Prot:P0DO10] |  |
| Q.CIM.stb.3BS.1 | Kukri_c88294_60 | 3B | 3904458 | 82.60869 | 3.026654 |  | MLMSTB PC6 ACROSS YEARS | TraesCS3B02G007210 | NA | NA |  |
| Q.CIM.stb.3BS.2 | AX-94394857 | 3B | 39910845 | -49.0643 | 3.984068 |  | BLINKSTB ACROSS YEARS | TraesCS3B02G067400 | chr3B:39905297-39910826(-) | NA |  |
| Q.CIM.stb.3BS.3 | CAP8_c8651_206 | 3B | 40246061 | 41.189 | 3.650904 |  | BLINKSTB ACROSS YEARS | TraesCS3B02G068000 | chr3B:40243799-40247161(-) | 14-3-3-like protein B [UniProtKB/Swiss-Prot:Q43470] |  |
| Q.CIM.stb.4AL.1 | wsnp_Ku_c1205_2398925 | 4A | 5.98E+08 | -61.0043 | 3.73299 |  | BLINKSTB2019 | TraesCS4A02G299700 | chr4A:597905176-597910407(+) | NA |  |
| Q.CIM.stb.4AL.2 | BobWhite_c20909_243 | 4A | 5.99E+08 | -66.7936 | 3.621641 |  | BLINKSTB2019 | TraesCS4A02G301600 | chr4A:598590265-598592384(+) | 2-methyl-6-phytyl-1,4-hydroquinone methyltransferase 2, chloroplastic [UniProtKB/Swiss-Prot:Q2QM69] |  |
| Q.CIM.stb.4AL.3 | AX-94467468 | 4A | 5.99E+08 | 70.86701 | 3.543723 |  | BLINKSTB2019 | TraesCS4A02G303400 | chr4A:599321764-599326140(+) | Uncharacterized oxidoreductase At1g06690, chloroplastic [UniProtKB/Swiss-Prot:Q94A68] |  |
| *Q.CIM.stb.4AL.4* | BS00092859_51 | 4A | 5.99E+08 | 0.21 | -70.87 | 3.54 | BLINKSTB2019 | NA |  |  |  |
| Q.CIM.stb.4AL.5 | RAC875_c78248_154 | 4A | 6.02E+08 | -76.087 | 3.059717 |  | MLMSTB2020 PC6 | TraesCS4A02G308800 | chr4A:602269460-602272127(-) | Cytochrome P450 72A14 [UniProtKB/Swiss-Prot:Q9LUC6] |  |
| Q.CIM.stb.4AL.6 | BS00021957_51 | 4A | 6.93E+08 | 34.57209 | 6.215447 | 2.03706 | FarmCPUSTB2021 | TraesCS4A02G422900 | chr4A:693274378-693278701(+) | Probable aminotransferase ACS10 [UniProtKB/Swiss-Prot:Q9LQ10] | 1-aminocyclopropane-1-carboxylate biosynthetic process |
|  |  |  |  |  |  |  |  | TraesCS4A02G422400 | chr4A:692545985-692551526(+) | Spastin [UniProtKB/Swiss-Prot:Q6AZT2] | axonal transport of mitochondrion [GO:0019896] |
|  |  |  |  |  |  |  |  | TraesCS4A02G422100 | chr4A:692382875-692386116(-) | 26S proteasome non-ATPase regulatory subunit 8 homolog A [UniProtKB/Swiss-Prot:Q9SGW3] | regulation of anthocyanin biosynthetic process [GO:0031540] |
|  |  |  |  |  |  |  |  | TraesCS4A02G423900 | chr4A:693783398-693785810(+) | Subtilisin-like protease 4 [UniProtKB/Swiss-Prot:A9QY38] | response to symbiotic bacterium [GO:0009609] |
|  |  |  |  |  |  |  |  | TraesCS4A02G422900 | chr4A:693274378-693278701(+) | Probable aminotransferase ACS10 [UniProtKB/Swiss-Prot:Q9LQ10] | #N/A |
| Q.CIM.stb.4AL.7 | TA001746-1415 | 4A | 7.34E+08 | 51.71376 | 3.767748 |  | FarmCPUSTB2019 | TraesCS4A02G474200 | chr4A:733705236-733711789(+) | NA |  |
| Q.CIM.stb.4BL.2 | IACX378 | 4B | 5.69E+08 | 960 | 3.727603 |  | BLINKSTB2021 | TraesCS4B02G285000 | chr4B:568555627-568559531(+) | Ribulose-phosphate 3-epimerase, chloroplastic [UniProtKB/Swiss-Prot:Q9ZTP5] | cellular carbohydrate metabolic process |
| Q.CIM.stb.4BL.3 | RAC875_c107130_384 | 4B | 6.49E+08 | -66.6332 | 3.733272 |  | BLINKSTB2019 | TraesCS4B02G358800 | chr4B:649469807-649475248(+) | Respiratory burst oxidase homolog protein B [UniProtKB/Swiss-Prot:Q5ZAJ0] |  |
| Q.CIM.stb.4BL.4 | Kukri_c18722_56 | 4B | 6.5E+08 | 69.42575 | 3.727149 |  | BLINKSTB2019 | TraesCS4B02G359300 | chr4B:649602163-649607404(-) | WD repeat-containing protein 44 [UniProtKB/Swiss-Prot:Q9XSC3] |  |
| Q.CIM.stb.4BL.5 | RAC875_c19259_1902 | 4B | 6.57E+08 | 1024 | 5.43806 | 1.329759 | BLINKSTB ACROSS YEARS | TraesCS4B02G371400 | chr4B:656903504-656911031(-) | Protein ARABIDILLO 1 [UniProtKB/Swiss-Prot:O22161] |  |
|  |  |  |  |  |  |  |  | TraesCS4B02G369200 | chr4B:654892610-654894282(+) | Anthranilate N-benzoyltransferase protein 1 [UniProtKB/Swiss-Prot:O24645] | #N/A |
|  |  |  |  |  |  |  |  | TraesCS4B02G375100 | chr4B:659154074-659173043(-) | CCR4-NOT transcription complex subunit 1 [UniProtKB/Swiss-Prot:Q6ZQ08] | gene silencing by miRNA [GO:0035195] |
|  |  |  |  |  |  |  |  | TraesCS4B02G371500 | chr4B:657159331-657160442(+) | BTB/POZ and MATH domain-containing protein 4 [UniProtKB/Swiss-Prot:Q9SRV1] | cellular response to salt stress [GO:0071472] |
|  |  |  |  |  |  |  |  | TraesCS4B02G372100 | chr4B:657243113-657246017(+) | Probable leucine-rich repeat receptor-like protein kinase At1g35710 [UniProtKB/Swiss-Prot:Q9LP24] | hormone-mediated signaling pathway [GO:0009755] |
|  |  |  |  |  |  |  |  | TraesCS4B02G374000 | chr4B:658166283-658167487(+) | DNA-directed RNA polymerases II, IV and V subunit 9A [UniProtKB/Swiss-Prot:Q6NLH0] | maintenance of transcriptional fidelity during DNA-templated transcription elongation from RNA polymerase II promoter [GO:0001193] |
|  |  |  |  |  |  |  |  | TraesCS4B02G371200 | chr4B:656891495-656893325(+) | UDP-glycosyltransferase 73C1 [UniProtKB/Swiss-Prot:Q9ZQ99] | #N/A |
| Q.CIM.stb.4BS.1 | AX-95148759 | 4B | 59232947 | -32.029 | 4.473369 |  | FarmCPU ACROSS YEARS | TraesCS4B02G066400 | chr4B:59231238-59234415(+) | Formate dehydrogenase, mitochondrial [UniProtKB/Swiss-Prot:Q9ZRI8] |  |
| Q.CIM.stb.4DL.1 | BS00103682_51 | 4D | 4.55E+08 | 33.18977 | 7.647112 | 1.340224 | FarmCPUSTB2021 | TraesCS4D02G283600 | chr4D:455341941-455346185(-) | Aluminum-activated malate transporter 1 [UniProtKB/Swiss-Prot:Q76LB1] | ion transmembrane transport |
| Q.CIM.stb.5AL.10 | BS00022071_51 | 5A | 5.87E+08 | -95.5077 | 3.349168 |  | MLMSTB2020 PC6 | NA | #N/A | #N/A |  |
| Q.CIM.stb.5AL.11 | wsnp_AJ612027A_Ta_2_1 | 5A | 5.89E+08 | 95.50774 | 3.349168 |  | MLMSTB2020 PC6 | TraesCS5A02G392300 | chr5A:588548725-588552987(+) | Solute carrier family 25 member 44 [UniProtKB/Swiss-Prot:Q8BGF9] | transmembrane transport |
| Q.CIM.stb.5AL.12 | IAAV8629 | 5A | 5.91E+08 | 36.84204 | 5.442344 | 4.896963 | FarmCPUSTB2021 | TraesCS5A02G396500 | chr5A:591313846-591320402(+) | NA |  |
| Q.CIM.stb.5AL.14 | BS00049403_51 | 5A | 6.07E+08 | -48.597 | 5.015898 | 1.699275 | FarmCPU ACROSS YEARS | TraesCS5A02G419900 | chr5A:606810994-606811710(+) | NA |  |
| Q.CIM.stb.5AL.2 | BS00110075_51 | 5A | 5.5E+08 | -39.9263 | 4.27292 |  | FarmCPU ACROSS YEARS | TraesCS5A02G347300 | chr5A:549797658-549800976(+) | Subtilisin-like protease SBT3.8 [UniProtKB/Swiss-Prot:Q9SZY3] | proteolysis |
| Q.CIM.stb.5AL.3 | BS00065551_51 | 5A | 5.57E+08 | 544 | 6.3446 | 5.270172 | BLINKSTB2020 | TraesCS5A02G353500 | chr5A:556529046-556531643(+) | NAC domain-containing protein 86 [UniProtKB/Swiss-Prot:Q9FFI5] | regulation of transcription, DNA-templated |
|  |  |  |  |  |  |  |  | TraesCS5A02G355000 | chr5A:557126728-557129781(-) | Protein PLASTID MOVEMENT IMPAIRED 1 [UniProtKB/Swiss-Prot:Q9C8E6] | abscisic acid homeostasis [GO:1902265] |
|  |  |  |  |  |  |  |  | TraesCS5A02G354500 | chr5A:557076709-557077548(+) | RING-H2 finger protein ATL80 [UniProtKB/Swiss-Prot:Q9LM69] | cellular response to cold [GO:0070417] |
|  |  |  |  |  |  |  |  | TraesCS5A02G355900 | chr5A:558304063-558305554(-) | Cytosolic sulfotransferase 5 [UniProtKB/Swiss-Prot:Q9M1V2] | flavonoid metabolic process [GO:0009812] |
|  |  |  |  |  |  |  |  | TraesCS5A02G356000 | chr5A:558321388-558331318(-) | Actin-related protein 4 [UniProtKB/Swiss-Prot:Q6ZJW9] | histone H4 acetylation [GO:0043967] |
|  |  |  |  |  |  |  |  | TraesCS5A02G355300 | chr5A:557319488-557323790(-) | Nicotinate-nucleotide pyrophosphorylase [carboxylating], chloroplastic [UniProtKB/Swiss-Prot:Q0IZS0] | NAD biosynthetic process [GO:0009435] |
|  |  |  |  |  |  |  |  | TraesCS5A02G353700 | chr5A:556675929-556684951(+) | UTP--glucose-1-phosphate uridylyltransferase [UniProtKB/Swiss-Prot:Q43772] | UDP-glucose metabolic process [GO:0006011] |
| Q.CIM.stb.5AL.4 | Tdurum_contig10210_425 | 5A | 5.7E+08 | -97.8193 | 12.21014 | 3.441174 | FarmCPU ACROSS YEARS | TraesCS5A02G370500 | chr5A:569863642-569869224(+) | Rhomboid-like protein 15 [UniProtKB/Swiss-Prot:Q8LB17] |  |
| Q.CIM.stb.5AL.6 | RAC875_c23775_406 | 5A | 5.72E+08 | 93.08433 | 3.640867 |  | MLMSTB2019PC6 | NA | #N/A | #N/A |  |
| Q.CIM.stb.5AL.7 | AX-94432465 | 5A | 5.73E+08 | -90.0279 | 3.406062 |  | MLMSTB2019 PC6 | TraesCS5A02G375000 | chr5A:573039922-573045194(+) | Ornithine aminotransferase, mitochondrial [UniProtKB/Swiss-Prot:Q10G56] | response to oxidative stress, response to water deprivation, defense response to bacterium, arginine catabolic process to glutamate, L-proline biosynthetic process, |
| Q.CIM.stb.5AL.8 | Tdurum_contig44343_1039 | 5A | 5.74E+08 | -89.1542 | 3.349322 |  | MLMSTB2019 PC6 | TraesCS5A02G375500 | chr5A:573586786-573591315(+) | BTB/POZ domain-containing protein At1g30440 [UniProtKB/Swiss-Prot:Q9S9Q9] | transcriptional regulation, cytoskeleton dynamics, ion channel assembly and gating, and targeting proteins for ubiquitination |
| Q.CIM.stb.5AL.9 | AX-94429192 | 5A | 5.81E+08 | 1664 | 7.678301 | 3.646174 | BLINKSTB2019 | TraesCS5A02G382700 | chr5A:580794356-580798659(+) | Probable histone H2A variant 3 [UniProtKB/Swiss-Prot:Q84MP7] |  |
|  |  |  |  |  |  |  |  | TraesCS5A02G381300 | chr5A:579242640-579243506(+) | BTB/POZ and MATH domain-containing protein 1 [UniProtKB/Swiss-Prot:Q8L765] | cellular response to salt stress [GO:0071472] |
|  |  |  |  |  |  |  |  | TraesCS5A02G381600 | chr5A:579409334-579410364(-) | Two-component response regulator ORR41 [UniProtKB/Swiss-Prot:Q75KW7] | cytokinin-activated signaling pathway [GO:0009736] |
|  |  |  |  |  |  |  |  | TraesCS5A02G383900 | chr5A:581572707-581574621(-) | UDP-glucose flavonoid 3-O-glucosyltransferase 7 [UniProtKB/Swiss-Prot:Q2V6J9] | #N/A |
|  |  |  |  |  |  |  |  | TraesCS5A02G382900 | chr5A:580802616-580806190(-) | ATP sulfurylase 4, chloroplastic [UniProtKB/Swiss-Prot:Q9S7D8] | hydrogen sulfide biosynthetic process [GO:0070814] |
|  |  |  |  |  |  |  |  | TraesCS5A02G384200 | chr5A:582099388-582104496(+) | Serine/threonine-protein kinase STY13 [UniProtKB/Swiss-Prot:Q9ZQ31] | #N/A |
|  |  |  |  |  |  |  |  | TraesCS5A02G383100 | chr5A:580944043-580947242(+) | General transcription factor 3C polypeptide 3 [UniProtKB/Swiss-Prot:Q9Y5Q9] | #N/A |
| Q.CIM.stb.5AS.1 | AX-94958984 | 5A | 34526445 | 101.603 | 4.12179 |  | MLMSTB2019PC6 | NA | #N/A | #N/A |  |
| Q.CIM.stb.5BL.1 | RAC875_c19105_498 | 5B | 4.32E+08 | 52.1403 | 4.108635 |  | FarmCPUSTB2020 | TraesCS5B02G249700 | chr5B:432174789-432178198(-) | Rhamnogalacturonan I rhamnosyltransferase 1 [UniProtKB/Swiss-Prot:Q4V398] | fucose metabolic process |
| Q.CIM.stb.5BL.2 | AX-94746298 | 5B | 5.61E+08 | -35.3584 | 3.647536 |  | FarmCPUSTB2020 | TraesCS5B02G382600 | chr5B:561061500-561065007(-) | Linoleate 9S-lipoxygenase 2 [UniProtKB/Swiss-Prot:P29250] | protein binding and metal ion binding; |
| Q.CIM.stb.5BL.3 | wsnp_Ex_c2870_5296539 | 5B | 6.81E+08 | 0 | 4.264746 |  | BLINKSTB2020 | TraesCS5B02G516400 | chr5B:680604273-680606613(-) | Glycine-rich RNA-binding protein RZ1A [UniProtKB/Swiss-Prot:Q9LIN3] |  |
| Q.CIM.stb.6AL.4 | wsnp_Ra_c27787_37299352 | 6A | 5.44E+08 | -57.4459 | 5.877371 | 2.342159 | FarmCPUSTB2019 | TraesCS6A02G309100 | chr6A:544204000-544213402(-) | NA |  |
| Q.CIM.stb.6AL.5 | BobWhite_s67148_292 | 6A | 5.82E+08 | -256 | 4.71436 |  | BLINKSTB2019 | TraesCS6A02G350400 | chr6A:582248516-582254826(+) | Protein DETOXIFICATION 19 [UniProtKB/Swiss-Prot:Q9LUH2] |  |
| Q.CIM.stb.6AS.3 | tplb0030n22_541 | 6A | 80690723 | -49.3172 | 3.801858 |  | FarmCPU ACROSS YEARS | TraesCS6A02G111200 | chr6A:80686000-80691297(+) | Protein ABC transporter 1, mitochondrial [UniProtKB/Swiss-Prot:Q9SBB2] |  |
| Q.CIM.stb.6DL.2 | AX-94633926 | 6D | 3.7E+08 | 62.4236 | 3.543652 |  | BLINKSTB2019 | TraesCS6D02G261300 | chr6D:369657989-369660932(+) | NA |  |
| Q.CIM.stb.6DL.3 | AX-94519170 | 6D | 4.65E+08 | -74.1355 | 3.166026 |  | MLMSTB2019 PC6 | TraesCS6D02G388300 | chr6D:464735277-464737794(-) | Rop guanine nucleotide exchange factor 3 [UniProtKB/Swiss-Prot:A4IJ27] |  |
| Q.CIM.stb.6DS.1 | RFL_Contig4626_873 | 6D | 91819922 | -121.281 | 3.331273 |  | MLMSTB2019 PC6 | TraesCS6D02G127700 | chr6D:91819732-91820932(-) | Probable glutathione S-transferase GSTU6 [UniProtKB/Swiss-Prot:Q06398] | glutathione metabolic process |
| Q.CIM.stb.7AL.2 | IAAV6287 | 7A | 6.45E+08 | 100.6087 | 6.161142 | 7.533103 | FarmCPUSTB2019 | NA | #N/A | #N/A |  |
| Q.CIM.stb.7AL.7 | AX-94404953 | 7D | 6.3E+08 | -56.6594 | 3.567555 |  | FarmCPUSTB2020 | TraesCS7D02G539500 | chr7D:629823548-629826613(+) | Aquaporin NIP1-3 [UniProtKB/Swiss-Prot:Q0DK16] | transmembrane transport |
| Q.CIM.stb.7AS.1 | AX-95103403 | 7A | 1.18E+08 | -24.2719 | 5.148811 | 4.760621 | FarmCPUSTB2021 | TraesCS7A02G161600 | chr7A:118184773-118187573(-) | Putative pentatricopeptide repeat-containing protein At5g08310, mitochondrial [UniProtKB/Swiss-Prot:P0C8Q6] |  |
| Q.CIM.stb_dh.1AS.1 | AX-95154820 | 1A | 3120139 | 56.69181 | 4.008231 |  | FarmCPUSTB2019 | TraesCS1A02G005500 | chr1A:3119378-3122276(-) | NA |  |
| Q.CIM.stb_dh.7AL.5 | wsnp_Ex_c3611_6599309 | 7A | 6.75E+08 | -84.5661 | 3.127973 |  | MLMSTB2019 PC6 | TraesCS7A02G483600 | chr7A:675112899-675115741(-) | NA |  |
| Q.CIM.stb_ph.1BS.1 | Excalibur_c29734_1152 | 1B | 1.49E+08 | 1504 | 5.791748 | 1.997294 | BLINKSTB2019 | NA | #N/A | #N/A |  |
| Q.CIM.stb_ph.2AL.3 | Kukri_c35153_956 | 2A | 3.82E+08 | -48.254 | 6.780147 | 1.280588 | FarmCPU ACROSS YEARS | NA_A | #N/A | #N/A |  |
| Q.CIM.stb_ph.7AL.4 | wsnp_JD_c20555_18262260 | 7A | 6.74E+08 | 79.27304 | 3.279815 |  | MLMSTB2019 PC6 | TraesCS7A02G482200 | chr7A:674276643-674280959(-) | ALA-interacting subunit 3 [UniProtKB/Swiss-Prot:Q9SLK2] |  |

**Table S4:** The unique quantitative trait nucleotides (QTNs) controlling the plant height (PH) and days to heading (DH)

| **Trait** | **QTNs** | **SNP** | **Chr** | **Pos** | **P.value** | **MAF** | **nobs** | **H&B.P.Value** | **Effect** | **LOG P VALUE** | **Years/Models** |
| --- | --- | --- | --- | --- | --- | --- | --- | --- | --- | --- | --- |
| PH | Q.CIM.ph.1AS.1 | Tdurum_contig13095_97 | 1A | 149352649 | 1.24E-07 | 0.112717 | 173 | 0.000668 | 1.880259 | 6.906436 | FarmCPU_ POOL |
| PH | Q.CIM.ph.1AL.2 | Ex_c5759_628 | 1A | 345108212 | 0.000465 | 0.092486 | 173 | 0.676153 | 4.283705 | 3.332682 | MLM_ POOL |
|  |  |  |  |  | 2.91E-05 | 0.092486 | 173 | 0.156731 | 7.638126 | 4.536231 | MLM_2020 |
| PH | Q.CIM.ph.1AL.3 | RFL_Contig5001_1099 | 1A | 353916288 | 0.00058 | 0.098266 | 173 | 0.676153 | -4.46737 | 3.236564 | MLM_ POOL |
|  |  |  |  |  | 9.03E-05 | 0.098266 | 173 | 0.243121 | -7.53957 | 4.044535 | MLM_2020 |
| PH | Q.CIM.ph.1BS.1 | BS00087784_51 | 1B | 50777928 | 0.000904 | 0.054913 | 173 | 0.856474 | 6.577153 | 3.043832 | MLM_2019 |
| PH | Q.CIM.ph.1BS.2 | BS00084305_51 | 1B | 90992706 | 5.24E-06 | 0.101156 | 173 | 0.014108 | 400 | 5.280902 | BLINK_2020 |
|  |  |  |  |  | 1.92E-11 | 0.101156 | 173 | 2.06E-07 | -4.32435 | 10.71779 | FarmCPU_2020 |
| PH | Q.CIM.stb_ph.1BS.1 | Excalibur_c29734_1152 | 1B | 148507500 | 0.000289 | 0.375723 | 173 | 0.472279 | -2.42513 | 3.539315 | FarmCPU_2021 |
| PH | Q.CIM.ph.1DS.1 | wsnp_Ra_c2633_5017265 | 1D | 8610041 | 0.000744 | 0.196532 | 173 | 0.676153 | 1.82527 | 3.128191 | MLM_ POOL |
|  |  |  |  |  | 7.76E-05 | 0.196532 | 173 | 0.836386 | 3.182755 | 4.110011 | MLM_2021 |
| PH | Q.CIM.ph.2AL.1 | wsnp_Ra_c14112_22155451 | 2A | 130174835 | 1.62E-07 | 0.086705 | 173 | 0.000438 | 4.606443 | 6.789156 | FarmCPU_2019 |
| PH | Q.CIM.ph.2AL.2 | AX-95249962 | 2A | 364479573 | 1.49E-07 | 0.104046 | 173 | 0.000438 | 4.093891 | 6.825759 | FarmCPU_2019 |
| PH | Q.CIM.stb_ph.2AL.3 | Kukri_c35153_956 | 2A | 381722087 | 0.000312 | 0.352601 | 173 | 0.472279 | 2.43194 | 3.505172 | FarmCPU_2021 |
| PH | Q.CIM.ph.2AL.3 | BS00067806_51 | 2A | 564620529 | 1.06E-05 | 0.234104 | 173 | 0.018146 | -1.17649 | 4.976201 | FarmCPU_ POOL |
| PH | Q.CIM.ph.2AL.4 | Jagger_c1810_163 | 2A | 598967566 | 1.91E-12 | 0.289017 | 173 | 2.06E-08 | 0 | 11.71867 | BLINK_2021 |
|  |  |  |  |  | 0.000252 | 0.289017 | 173 | 0.472279 | -2.61143 | 3.598798 | FarmCPU_2021 |
|  |  |  |  |  | 0.000868 | 0.289017 | 173 | 0.916291 | -3.12309 | 3.061406 | MLM_2021 |
| PH | Q.CIM.ph.2AL.5 | wsnp_Ex_c8894_14858193 | 2A | 602440025 | 0.000273 | 0.367052 | 173 | 0.472279 | 2.438404 | 3.563459 | FarmCPU_2021 |
| PH | Q.CIM.ph.2AL.6 | BS00049644_51 | 2A | 605184906 | 0.000791 | 0.193642 | 173 | 0.916291 | -2.95848 | 3.101726 | MLM_2021 |
| PH | Q.CIM.ph.2BS.1 | RAC875_c27611_467 | 2B | 1251390 | 0.000236 | 0.176301 | 173 | 0.317222 | -1.98872 | 3.627963 | BLINK_2020 |
| PH | Q.CIM.ph.2BS.2 | AX-94831339 | 2B | 3405723 | 0.000215 | 0.17341 | 173 | 0.317222 | -2.01549 | 3.666779 | BLINK_2020 |
| PH | Q.CIM.ph.2BS.3 | RAC875_c62831_255 | 2B | 17564757 | 1.08E-06 | 0.080925 | 173 | 0.003861 | 3.105693 | 5.968561 | FarmCPU_2020 |
| PH | Q.CIM.ph.2BS.4 | AX-94397938 | 2B | 253716707 | 0.000939 | 0.066474 | 173 | 0.856474 | 4.59566 | 3.027476 | MLM_2019 |
| PH | Q.CIM.ph.3AS.1 | RFL_Contig3591_1832 | 3A | 22616253 | 0.00017 | 0.17341 | 173 | 0.30537 | -1.59731 | 3.76944 | BLINK _ POOL |
| PH | Q.CIM.ph.3AL.2 | RAC875_c27323_867 | 3A | 480438501 | 0.000476 | 0.15896 | 173 | 0.676153 | -2.77555 | 3.321978 | MLM_ POOL |
| PH | Q.CIM.ph.3AL.3 | BS00021930_51 | 3A | 514111005 | 8.48E-05 | 0.199422 | 173 | 0.241315 | -32 | 4.071379 | BLINK_2021 |
| PH | Q.CIM.ph.3AL.4 | Ku_c26872_269 | 3A | 729756576 | 0.000213 | 0.398844 | 173 | 0.460032 | 0 | 3.67066 | BLINK_2021 |
| PH | Q.CIM.ph.3BS.1 | IAAV3924 | 3B | 20450524 | 0.000252 | 0.184971 | 173 | 0.379218 | 1.531785 | 3.598897 | BLINK _ POOL |
| PH | Q.CIM.ph.3BS.2 | AX-95008150 | 3B | 20720572 | 6.67E-05 | 0.182081 | 173 | 0.235117 | 1.674723 | 4.176112 | BLINK _ POOL |
|  |  |  |  |  | 1.46E-06 | 0.182081 | 173 | 0.003938 | 1.489574 | 5.835044 | FarmCPU_ POOL |
| PH | Q.CIM.ph.3BL.3 | AX-94634237 | 3B | 449812045 | 0.0001 | 0.078035 | 173 | 0.235117 | -256 | 3.998216 | BLINK _ POOL |
|  |  |  |  |  | 3.56E-06 | 0.078035 | 173 | 0.007678 | 4.188779 | 5.448184 | FarmCPU_2019 |
| PH | Q.CIM.ph.3BL.4 | Excalibur_c15332_1194 | 3B | 578812799 | 0.000255 | 0.303468 | 173 | 0.30557 | -1.86195 | 3.593065 | FarmCPU_2019 |
| PH | Q.CIM.ph.3BL.5 | BS00023017_51 | 3B | 779366860 | 0.000149 | 0.138728 | 173 | 0.178131 | -1.84404 | 3.827434 | FarmCPU_2020 |
| PH | Q.CIM.ph.4AL.1 | wsnp_JD_rep_c51623_35119179 | 4A | 486124896 | 3.81E-05 | 0.135838 | 173 | 0.058705 | -2.74557 | 4.418645 | FarmCPU_2019 |
| PH | Q.CIM.ph.4AL.2 | Kukri_c52413_282 | 4A | 590588763 | 7.95E-07 | 0.066474 | 173 | 0.008565 | 51 | 6.099687 | BLINK_2019 |
|  |  |  |  |  | 1.74E-06 | 0.066474 | 173 | 0.014108 | 48 | 5.76004 | BLINK_2020 |
|  |  |  |  |  | 1.18E-05 | 0.066474 | 173 | 0.018146 | -2.18349 | 4.928531 | FarmCPU_ POOL |
|  |  |  |  |  | 2.88E-06 | 0.066474 | 173 | 0.007755 | -3.75875 | 5.540794 | FarmCPU_2020 |
|  |  |  |  |  | 0.000209 | 0.066474 | 173 | 0.676153 | -2.97327 | 3.680189 | MLM_ POOL |
|  |  |  |  |  | 0.000671 | 0.066474 | 173 | 0.856474 | -4.96172 | 3.173058 | MLM_2019 |
|  |  |  |  |  | 1.76E-06 | 0.066474 | 173 | 0.018913 | -5.85972 | 5.75565 | MLM_2020 |
| PH | Q.CIM.ph.4BL.1 | BobWhite_rep_c66957_84 | 4B | 599553452 | 0.000326 | 0.228324 | 173 | 0.856474 | 4.930359 | 3.486379 | MLM_2019 |
| PH | Q.CIM.ph.5AL.1 | Ex_c1344_1859 | 5A | 104625383 | 0.000109 | 0.080925 | 173 | 0.235117 | 48 | 3.962163 | BLINK _ POOL |
|  |  |  |  |  | 0.000165 | 0.080925 | 173 | 0.295752 | 68 | 3.783339 | BLINK_2020 |
|  |  |  |  |  | 2.08E-08 | 0.080925 | 173 | 0.000224 | -3.01449 | 7.681644 | FarmCPU_ POOL |
|  |  |  |  |  | 1.04E-06 | 0.080925 | 173 | 0.003861 | -3.25791 | 5.983237 | FarmCPU_2020 |
| PH | Q.CIM.ph.5AL.2 | wsnp_Ex_c2132_4004831 | 5A | 403353551 | 0.000286 | 0.112717 | 173 | 0.30869 | -3.72027 | 3.542895 | MLM_2020 |
| PH | Q.CIM.ph.5AL.3 | wsnp_Ex_c40022_47169698 | 5A | 404533251 | 0.000286 | 0.112717 | 173 | 0.30869 | -3.72027 | 3.542895 | MLM_2020 |
| PH | Q.CIM.ph.5AL.4 | wsnp_BE443187A_Ta_2_3 | 5A | 450164758 | 5.06E-05 | 0.294798 | 173 | 0.09019 | -1.30799 | 4.295902 | FarmCPU_2020 |
| PH | Q.CIM.ph.5AL.5 | wsnp_Ex_c19724_28721128 | 5A | 503137639 | 2.02E-08 | 0.196532 | 173 | 0.000109 | 80 | 7.69515 | BLINK_2021 |
|  |  |  |  |  | 0.00022 | 0.196532 | 173 | 0.472279 | -3.14621 | 3.658225 | FarmCPU_2021 |
|  |  |  |  |  | 0.000648 | 0.196532 | 173 | 0.916291 | -3.18046 | 3.188314 | MLM_2021 |
| PH | Q.CIM.ph.5AL.6 | IACX5390 | 5A | 605101626 | 0.000104 | 0.286127 | 173 | 0.139814 | -1.30761 | 3.983777 | FarmCPU_2020 |
| PH | Q.CIM.ph.5BS.1 | RAC875_rep_c116173_605 | 5B | 35289978 | 2.72E-06 | 0.153179 | 173 | 0.014108 | 56 | 5.565291 | BLINK_2020 |
| PH | Q.CIM.ph.5BS.2 | Excalibur_c14594_182 | 5B | 38157606 | 0.000123 | 0.265896 | 173 | 0.147323 | 0.950386 | 3.909904 | FarmCPU_ POOL |
|  |  |  |  |  | 1.18E-07 | 0.265896 | 173 | 0.000438 | 2.956642 | 6.929789 | FarmCPU_2019 |
|  |  |  |  |  | 0.00071 | 0.265896 | 173 | 0.676153 | 1.853555 | 3.149024 | MLM_ POOL |
|  |  |  |  |  | 0.000423 | 0.265896 | 173 | 0.856474 | 3.486181 | 3.373877 | MLM_2019 |
| PH | Q.CIM.ph.5BS.3 | AX-94606697 | 5B | 59162669 | 2.54E-05 | 0.228324 | 173 | 0.137107 | 68 | 4.594328 | BLINK _ POOL |
|  |  |  |  |  | 0.000847 | 0.228324 | 173 | 0.676153 | -1.91873 | 3.072271 | MLM_ POOL |
| PH | Q.CIM.ph.5BL.4 | Kukri_c36789_230 | 5B | 355618301 | 0.000286 | 0.112717 | 173 | 0.30869 | 3.72027 | 3.542895 | MLM_2020 |
| PH | Q.CIM.ph.5BL.5 | wsnp_Ex_c5632_9904112 | 5B | 356186273 | 0.000262 | 0.115607 | 173 | 0.30869 | -3.73498 | 3.581421 | MLM_2020 |
| PH | Q.CIM.ph.5DL.1 | wsnp_Ex_c19724_28720939 | 5D | 398107597 | 0.000254 | 0.187861 | 173 | 0.472279 | -3.09487 | 3.594409 | FarmCPU_2021 |
| PH | Q.CIM.ph.6AS.1 | Kukri_c21943_958 | 6A | 70419120 | 3.23E-05 | 0.320809 | 173 | 0.058064 | 1.779232 | 4.490358 | FarmCPU_2019 |
| PH | Q.CIM.ph.6AS.2 | RAC875_c17189_155 | 6A | 105181461 | 2.15E-07 | 0.095376 | 173 | 0.00232 | 384 | 6.666966 | BLINK _ POOL |
|  |  |  |  |  | 2.07E-06 | 0.095376 | 173 | 0.004462 | 1.946008 | 5.683876 | FarmCPU_ POOL |
|  |  |  |  |  | 7.95E-06 | 0.095376 | 173 | 0.017122 | 2.982801 | 5.099883 | FarmCPU_2020 |
|  |  |  |  |  | 6.46E-05 | 0.095376 | 173 | 0.231943 | 4.374366 | 4.189915 | MLM_2020 |
| PH | Q.CIM.ph.6AL.3 | Kukri_rep_c104521_601 | 6A | 564270705 | 3.22E-05 | 0.144509 | 173 | 0.069362 | 64 | 4.492323 | BLINK_2020 |
|  |  |  |  |  | 5.86E-05 | 0.144509 | 173 | 0.09019 | -2.09869 | 4.232162 | FarmCPU_2020 |
|  |  |  |  |  | 0.000269 | 0.144509 | 173 | 0.30869 | -3.48257 | 3.571037 | MLM_2020 |
| PH | Q.CIM.ph.6AL.4 | Kukri_c6128_319 | 6A | 564272520 | 0.000269 | 0.144509 | 173 | 0.30869 | -3.48257 | 3.571037 | MLM_2020 |
| PH | Q.CIM.ph.6AL.5 | BobWhite_c5255_429 | 6A | 603280457 | 0.000301 | 0.095376 | 173 | 0.324042 | 2.751589 | 3.521816 | FarmCPU_2019 |
| PH | Q.CIM.ph.6BL.1 | wsnp_Ex_c5177_9174930 | 6B | 694678780 | 5.04E-05 | 0.104046 | 173 | 0.067884 | 1.494412 | 4.297562 | FarmCPU_ POOL |
| PH | Q.CIM.ph.7AS.1 | BobWhite_c14495_230 | 7A | 14189397 | 4.86E-06 | 0.297688 | 173 | 0.014108 | 72 | 5.313139 | BLINK_2020 |
| PH | Q.CIM.ph.7AS.2 | wsnp_Ex_c23755_32994701 | 7A | 95806762 | 0.000312 | 0.300578 | 173 | 0.591222 | 57 | 3.506103 | BLINK_2019 |
| PH | Q.CIM.ph.7AS.3 | Ku_c9598_2119 | 7A | 205171484 | 2.66E-07 | 0.49422 | 173 | 0.000957 | -1.22738 | 6.574597 | FarmCPU_ POOL |
| PH | Q.CIM.ph.7AL.4 | wsnp_Ku_c17161_26193994 | 7A | 660450478 | 7.02E-05 | 0.210983 | 173 | 0.094607 | 2.038451 | 4.153405 | FarmCPU_2019 |
| PH | Q.CIM.stb_ph.7AL.4 | wsnp_JD_c20555_18262260 | 7A | 674276749 | 0.0009 | 0.312139 | 173 | 0.676153 | -1.64912 | 3.045715 | MLM_ POOL |
| PH | Q.CIM.ph.7AL.5 | wsnp_JD_c20555_18262317 | 7A | 674276806 | 0.000826 | 0.34104 | 173 | 0.676153 | 1.590347 | 3.083138 | MLM_ POOL |
| PH | Q.CIM.ph.7BS.1 | Ra_c56305_1946 | 7B | 159142231 | 1.30E-07 | 0.488439 | 173 | 0.000438 | -2.42142 | 6.885147 | FarmCPU_2019 |
| PH | Q.CIM.ph.7BL.2 | AX-94767893 | 7B | 741575987 | 0.000414 | 0.390173 | 173 | 0.676153 | 1.740548 | 3.38252 | MLM_ POOL |
| PH | Q.CIM.ph.7BL.3 | Tdurum_contig30909_76 | 7B | 743304117 | 8.96E-05 | 0.393064 | 173 | 0.241315 | 48 | 4.047774 | BLINK_2021 |
| DH | Q.CIM.stb_dh.1AS.1 | AX-95154820 | 1A | 3120139 | 0.000262 | 0.141618 | 173 | 0.831425 | 36 | 3.581285 | BLINK_2019 |
|  |  |  |  |  | 4.48E-06 | 0.141618 | 173 | 0.013697 | -1.29993 | 5.349141 | FarmCPU_2019 |
|  |  |  |  |  | 0.000567 | 0.141618 | 173 | 0.96198 | -2.08589 | 3.246752 | MLM_2019 |
| DH | Q.CIM.dh.1AS.1 | RAC875_c38756_141 | 1A | 7335009 | 2.78E-05 | 0.268786 | 173 | 0.03333 | -0.68923 | 4.555343 | FarmCPU_2020 |
| DH | Q.CIM.dh.1AL.1 | Excalibur_c59894_97 | 1A | 463813596 | 0.000587 | 0.066474 | 173 | 0.646916 | 2.07606 | 3.231537 | MLM_POOL |
|  |  |  |  |  | 0.000374 | 0.066474 | 173 | 0.686823 | 2.928048 | 3.426922 | MLM_2021 |
| DH | Q.CIM.dh.1AL.2 | AX-94550326 | 1A | 464116406 | 0.000876 | 0.063584 | 173 | 0.674388 | -2.18664 | 3.05738 | MLM_POOL |
|  |  |  |  |  | 0.000995 | 0.063584 | 173 | 0.809624 | -2.93403 | 3.002204 | MLM_2021 |
| DH | Q.CIM.dh.1AL.3 | Ra_c58315_265 | 1A | 473637106 | 0.000258 | 0.054913 | 173 | 0.397376 | 416 | 3.588117 | BLINK_2021 |
|  |  |  |  |  | 1.40E-05 | 0.054913 | 173 | 0.018887 | 1.839411 | 4.853168 | FarmCPU_2021 |
| DH | Q.CIM.dh.1AL.4 | AX-94408000 | 1A | 550444223 | 0.000245 | 0.476879 | 173 | 0.240062 | 0.523558 | 3.610701 | FarmCPU_POOL |
| DH | Q.CIM.dh.1AL.5 | AX-94796921 | 1B | 38067645 | 4.79E-06 | 0.130058 | 173 | 0.013697 | 1.294109 | 5.319593 | FarmCPU_2019 |
| DH | Q.CIM.dh.1BL.1 | wsnp_Ex_c1440_2764867 | 1B | 551177422 | 1.95E-05 | 0.33526 | 173 | 0.105294 | 120 | 4.708983 | BLINK_2020 |
|  |  |  |  |  | 9.27E-05 | 0.33526 | 173 | 0.090827 | -0.54843 | 4.03281 | FarmCPU_2020 |
| DH | Q.CIM.dh.1BL.2 | TA004668-0687 | 1B | 643097047 | 1.18E-06 | 0.398844 | 173 | 0.012745 | -0.91781 | 5.927072 | FarmCPU_2019 |
| DH | Q.CIM.dh.2AS.1 | Kukri_c3701_392 | 2A | 1370472 | 1.80E-05 | 0.488439 | 173 | 0.038843 | 70 | 4.744135 | BLINK_2021 |
| DH | Q.CIM.dh.2AL.2 | RAC875_c96033_191 | 2A | 753617305 | 3.47E-08 | 0.312139 | 173 | 0.000187 | -0.77318 | 7.460227 | FarmCPU_POOL |
| DH | Q.CIM.dh.2AL.3 | BS00091763_51 | 2A | 773185993 | 0.000606 | 0.306358 | 173 | 0.646916 | -0.94778 | 3.217629 | MLM_POOL |
|  |  |  |  |  | 0.000249 | 0.306358 | 173 | 0.66943 | -1.23805 | 3.604652 | MLM_2020 |
| DH | Q.CIM.dh.2BL.1 | Excalibur_c33267_538 | 2B | 729180151 | 0.000498 | 0.138728 | 173 | 0.646916 | -1.23543 | 3.302539 | MLM_POOL |
| DH | Q.CIM.dh.2BL.2 | Excalibur_c44325_638 | 2B | 741445277 | 4.91E-06 | 0.268786 | 173 | 0.010113 | 0.873208 | 5.309011 | FarmCPU_2021 |
| DH | Q.CIM.stb_dh.2BL.9 | AX-95084761 | 2B | 784545663 | 0.000672 | 0.213873 | 173 | 0.646916 | 1.086337 | 3.172749 | MLM_POOL |
|  |  |  |  |  | 0.000183 | 0.213873 | 173 | 0.657027 | 1.659583 | 3.737713 | MLM_2021 |
| DH | Q.CIM.dh.2BL.3 | BS00004413_51 | 2B | 784551315 | 4.79E-05 | 0.075145 | 173 | 0.258312 | -2.0375 | 4.319243 | MLM_POOL |
|  |  |  |  |  | 0.000931 | 0.075145 | 173 | 0.96198 | -2.14786 | 3.031024 | MLM_2019 |
|  |  |  |  |  | 7.82E-06 | 0.075145 | 173 | 0.042114 | -3.11265 | 5.106958 | MLM_2021 |
| DH | Q.CIM.stb_dh.2DL.3 | AX-94787485 | 2D | 590930925 | 2.53E-06 | 0.179191 | 173 | 0.004749 | 44 | 5.596513 | BLINK_POOL |
| DH | Q.CIM.dh.3AS.1 | wsnp_Ra_c2228_4310870 | 3A | 140046639 | 2.64E-06 | 0.101156 | 173 | 0.004749 | 0 | 5.577701 | BLINK_POOL |
| DH | Q.CIM.dh.3AL.1 | Ra_c4373_453 | 3A | 720436380 | 0.000153 | 0.138728 | 173 | 0.831425 | -1.31323 | 3.816416 | BLINK_2019 |
|  |  |  |  |  | 0.000407 | 0.138728 | 173 | 0.646916 | -1.24891 | 3.389931 | MLM_POOL |
|  |  |  |  |  | 1.59E-05 | 0.138728 | 173 | 0.170931 | -2.02562 | 4.799596 | MLM_2019 |
| DH | Q.CIM.stb_dh.3AL.6 | RAC875_c61934_186 | 3A | 733995575 | 5.63E-06 | 0.236994 | 173 | 0.010113 | 0.94587 | 5.249372 | FarmCPU_2021 |
| DH | Q.CIM.dh.3BS.1 | AX-94660868 | 3B | 4328430 | 0.000113 | 0.124277 | 173 | 0.122035 | 0.988455 | 3.945932 | FarmCPU_POOL |
| DH | Q.CIM.dh.3BL.2 | Kukri_c66862_96 | 3B | 745220324 | 0.000637 | 0.473988 | 173 | 0.686533 | 1.062318 | 3.195756 | MLM_2020 |
| DH | Q.CIM.dh.3BL.3 | Ku_c24974_674 | 3B | 747712577 | 8.12E-09 | 0.485549 | 173 | 4.38E-05 | 74 | 8.090208 | BLINK_POOL |
|  |  |  |  |  | 1.36E-06 | 0.485549 | 173 | 0.004891 | 68 | 5.865859 | BLINK_2021 |
|  |  |  |  |  | 9.42E-08 | 0.485549 | 173 | 0.000338 | 0.748358 | 7.025827 | FarmCPU_POOL |
|  |  |  |  |  | 0.000191 | 0.485549 | 173 | 0.147106 | 0.628093 | 3.718658 | FarmCPU_2020 |
|  |  |  |  |  | 0.00036 | 0.485549 | 173 | 0.646916 | 0.986151 | 3.443489 | MLM_POOL |
|  |  |  |  |  | 3.76E-05 | 0.485549 | 173 | 0.404636 | 1.239362 | 4.425353 | MLM_2020 |
| DH | Q.CIM.dh.3BL.4 | AX-94439075 | 3B | 756265102 | 0.000217 | 0.381503 | 173 | 0.66943 | -1.1026 | 3.664237 | MLM_2020 |
| DH | Q.CIM.dh.3BL.5 | AX-94756413 | 3B | 791287062 | 0.000372 | 0.294798 | 173 | 0.96198 | -1.51154 | 3.429405 | MLM_2019 |
| DH | Q.CIM.dh.3DL.1 | AX-95165551 | 3D | 569524161 | 0.000147 | 0.419075 | 173 | 0.121943 | -0.6348 | 3.832316 | FarmCPU_2020 |
|  |  |  |  |  | 0.000182 | 0.419075 | 173 | 0.66943 | -1.05019 | 3.739432 | MLM_2020 |
| DH | Q.CIM.dh.4AS.1 | BS00068244_51 | 4A | 46125511 | 1.56E-08 | 0.066474 | 173 | 5.62E-05 | 34 | 7.805663 | BLINK_POOL |
|  |  |  |  |  | 7.78E-06 | 0.066474 | 173 | 0.02096 | 35 | 5.108962 | BLINK_2021 |
|  |  |  |  |  | 1.92E-05 | 0.066474 | 173 | 0.025824 | -1.36005 | 4.717306 | FarmCPU_POOL |
| DH | Q.CIM.dh.4AS.2 | wsnp_Ex_c6044_10590220 | 4A | 101510364 | 2.70E-07 | 0.17341 | 173 | 0.000728 | -0.97806 | 6.568239 | FarmCPU_POOL |
| DH | Q.CIM.dh.4AL.3 | BS00072025_51 | 4A | 605664279 | 1.04E-08 | 0.083815 | 173 | 0.000112 | 1.76435 | 7.982171 | FarmCPU_2020 |
| DH | Q.CIM.dh.4AL.4 | wsnp_Ex_c19207_28125072 | 4A | 611580737 | 2.28E-05 | 0.193642 | 173 | 0.040885 | -0.98435 | 4.642707 | FarmCPU_2019 |
| DH | Q.CIM.dh.4BL.1 | RAC875_c1357_860 | 4B | 609498098 | 5.08E-06 | 0.352601 | 173 | 0.013697 | 0.935393 | 5.293724 | FarmCPU_2019 |
| DH | Q.CIM.dh.4DS.1 | IAAV1674 | 4D | 1876348 | 9.34E-07 | 0.251445 | 173 | 0.002516 | 46 | 6.029674 | BLINK_POOL |
|  |  |  |  |  | 1.14E-07 | 0.251445 | 173 | 0.000616 | 44 | 6.941515 | BLINK_2021 |
|  |  |  |  |  | 2.67E-07 | 0.251445 | 173 | 0.001439 | 1.026527 | 6.573402 | FarmCPU_2021 |
| DH | Q.CIM.dh.5AS.1 | IAAV5061 | 5A | 91119501 | 1.76E-05 | 0.092486 | 173 | 0.02107 | -5.28491 | 4.754513 | BLINK_POOL |
| DH | Q.CIM.dh.5AS.2 | Ku_c69633_1873 | 5A | 97227265 | 1.76E-05 | 0.092486 | 173 | 0.02107 | -5.28491 | 4.754513 | BLINK_POOL |
| DH | Q.CIM.dh.5AS.3 | wsnp_Ku_c328_679106 | 5A | 104232188 | 1.76E-05 | 0.092486 | 173 | 0.02107 | -5.28491 | 4.754513 | BLINK_POOL |
| DH | Q.CIM.dh.5AL.4 | Excalibur_c1208_72 | 5A | 502219443 | 8.95E-05 | 0.369942 | 173 | 0.107193 | 0.453575 | 4.048009 | FarmCPU_POOL |
| DH | Q.CIM.dh.5AL.5 | BS00005860_51 | 5A | 598245820 | 0.000446 | 0.323699 | 173 | 0.686823 | -1.64331 | 3.350987 | MLM_2021 |
| DH | Q.CIM.dh.5AL.6 | BS00021968_51 | 5A | 677631865 | 0.000597 | 0.456647 | 173 | 0.96198 | -1.35866 | 3.223752 | MLM_2019 |
| DH | Q.CIM.dh.5BS.1 | Kukri_c1468_1609 | 5B | 65772685 | 3.52E-05 | 0.248555 | 173 | 0.063155 | 128 | 4.45386 | BLINK_2021 |
|  |  |  |  |  | 0.000815 | 0.248555 | 173 | 0.809624 | 1.413648 | 3.088604 | MLM_2021 |
| DH | Q.CIM.dh.5BL.2 | AX-94435238 | 5B | 581132597 | 0.0003 | 0.075145 | 173 | 0.59812 | 32 | 3.523524 | BLINK_2020 |
|  |  |  |  |  | 4.87E-06 | 0.075145 | 173 | 0.007814 | -1.28364 | 5.312391 | FarmCPU_POOL |
|  |  |  |  |  | 4.54E-05 | 0.075145 | 173 | 0.048891 | -1.3445 | 4.343192 | FarmCPU_2021 |
| DH | Q.CIM.dh.5BS.1 | AX-94663690 | 5D | 45354865 | 4.73E-07 | 0.17052 | 173 | 0.001698 | -1.23448 | 6.325339 | FarmCPU_2021 |
| DH | Q.CIM.dh.6AL.1 | Tdurum_contig97355_136 | 6A | 591729142 | 0.000581 | 0.291908 | 173 | 0.646916 | -0.98069 | 3.236097 | MLM_POOL |
|  |  |  |  |  | 0.00085 | 0.291908 | 173 | 0.809624 | -1.34648 | 3.070719 | MLM_2021 |
| DH | Q.CIM.dh.6AL.2 | Tdurum_contig97355_194 | 6A | 591729183 | 0.00072 | 0.283237 | 173 | 0.646916 | 0.946336 | 3.142388 | MLM_POOL |
| DH | Q.CIM.dh.6AL.2 | Excalibur_c2737_309 | 6A | 591732019 | 0.000408 | 0.286127 | 173 | 0.646916 | 0.998431 | 3.389104 | MLM_POOL |
|  |  |  |  |  | 0.000333 | 0.286127 | 173 | 0.686823 | 1.43775 | 3.477861 | MLM_2021 |
| DH | Q.CIM.dh.6AL.3 | Excalibur_c25390_2483 | 6A | 605231868 | 2.26E-05 | 0.343931 | 173 | 0.030469 | 0.633782 | 4.645463 | FarmCPU_2020 |
| DH | Q.CIM.dh.6AL.4 | RAC875_c12821_466 | 6A | 615439091 | 1.79E-06 | 0.187861 | 173 | 0.00385 | 0.952823 | 5.747936 | FarmCPU_2020 |
| DH | Q.CIM.dh.6AL.5 | Tdurum_contig70819_393 | 6A | 615821812 | 0.000291 | 0.066474 | 173 | 0.309097 | -1.11338 | 3.536302 | BLINK_POOL |
| DH | Q.CIM.dh.6BS.1 | AX-94408875 | 6B | 23653379 | 1.88E-11 | 0.078035 | 173 | 2.03E-07 | 41 | 10.72517 | BLINK_POOL |
|  |  |  |  |  | 7.32E-14 | 0.078035 | 173 | 7.89E-10 | 44 | 13.13553 | BLINK_2021 |
|  |  |  |  |  | 8.58E-16 | 0.078035 | 173 | 9.24E-12 | -2.24175 | 15.06652 | FarmCPU_POOL |
|  |  |  |  |  | 1.83E-05 | 0.078035 | 173 | 0.039442 | -1.28236 | 4.737484 | FarmCPU_2019 |
|  |  |  |  |  | 2.04E-13 | 0.078035 | 173 | 2.20E-09 | -2.63482 | 12.69049 | FarmCPU_2021 |
|  |  |  |  |  | 4.58E-06 | 0.078035 | 173 | 0.049397 | -2.41013 | 5.338713 | MLM_POOL |
|  |  |  |  |  | 3.27E-05 | 0.078035 | 173 | 0.175938 | -2.81931 | 4.486029 | MLM_2019 |
|  |  |  |  |  | 0.000534 | 0.078035 | 173 | 0.686533 | -1.90883 | 3.272678 | MLM_2020 |
|  |  |  |  |  | 3.12E-06 | 0.078035 | 173 | 0.03364 | -3.37524 | 5.505559 | MLM_2021 |
| DH | Q.CIM.dh.6BS.2 | wsnp_Ex_rep_c115803_95396724 | 6B | 25256244 | 0.000108 | 0.465318 | 173 | 0.096536 | -0.55862 | 3.968545 | FarmCPU_2020 |
| DH | Q.CIM.dh.6BL.3 | RAC875_c68525_284 | 6B | 657946476 | 1.55E-07 | 0.150289 | 173 | 0.000557 | -1.06052 | 6.809469 | FarmCPU_2020 |
| DH | Q.CIM.dh.6DS.1 | wsnp_Ex_c14439_22426200 | 6D | 2406518 | 6.17E-05 | 0.482659 | 173 | 0.066524 | -0.67278 | 4.20944 | FarmCPU_2020 |
|  |  |  |  |  | 0.000805 | 0.482659 | 173 | 0.666953 | -0.83462 | 3.094379 | MLM_POOL |
|  |  |  |  |  | 0.000463 | 0.482659 | 173 | 0.686533 | -0.97516 | 3.334252 | MLM_2020 |
| DH | Q.CIM.dh.6DS.2 | TA002853-0110-w | 6D | 2408966 | 0.000523 | 0.459538 | 173 | 0.686533 | 0.916224 | 3.281446 | MLM_2020 |
| DH | Q.CIM.dh.6DL.3 | AX-94765421 | 6D | 445497018 | 0.000446 | 0.291908 | 173 | 0.686823 | -1.33071 | 3.350474 | MLM_2021 |
| DH | Q.CIM.dh.7AS.1 | Tdurum_contig93663_457 | 7A | 18876986 | 5.08E-06 | 0.476879 | 173 | 0.007814 | -0.62214 | 5.294464 | FarmCPU_POOL |
| DH | Q.CIM.dh.7AS.2 | Tdurum_contig12722_779 | 7A | 46720779 | 2.36E-06 | 0.346821 | 173 | 0.004239 | -0.82025 | 5.627013 | FarmCPU_2020 |
| DH | Q.CIM.dh.7AS.3 | AX-94388916 | 7A | 58238172 | 2.47E-08 | 0.17052 | 173 | 0.000133 | -1.03199 | 7.607723 | FarmCPU_2020 |
| DH | Q.CIM.dh.7AL.4 | IAAV6170 | 7A | 538784257 | 0.000506 | 0.098266 | 173 | 0.96198 | -1.96278 | 3.29583 | MLM_2019 |
| DH | Q.CIM.dh.7AL.5 | wsnp_Ku_c60707_62509051 | 7A | 659372722 | 1.43E-05 | 0.277457 | 173 | 0.022028 | 0.540892 | 4.844351 | FarmCPU_2020 |
| DH | Q.CIM.dh.7AL.6 | BS00009838_51 | 7A | 666607524 | 4.87E-06 | 0.072254 | 173 | 0.010113 | 1.340472 | 5.312081 | FarmCPU_2021 |
| DH | Q.CIM.stb_dh.7AL.5 | wsnp_Ex_c3611_6599309 | 7A | 675113102 | 2.88E-05 | 0.147399 | 173 | 0.034486 | 0.95936 | 4.540533 | FarmCPU_2021 |
| DH | Q.CIM.dh.7AL.7 | wsnp_Ex_rep_c101269_86664147 | 7A | 704793025 | 7.00E-06 | 0.132948 | 173 | 0.01078 | -1.03245 | 5.154683 | FarmCPU_2021 |
| DH | Q.CIM.dh.7AL.8 | BS00010819_51 | 7A | 723259910 | 1.57E-05 | 0.179191 | 173 | 0.105294 | 42 | 4.803512 | BLINK_2020 |
|  |  |  |  |  | 1.21E-06 | 0.179191 | 173 | 0.003261 | -1.00887 | 5.917012 | FarmCPU_2020 |
|  |  |  |  |  | 0.000543 | 0.179191 | 173 | 0.686533 | -1.36456 | 3.264954 | MLM_2020 |
| DH | Q.CIM.dh.7BS.1 | AX-94545252 | 7B | 133792834 | 0.000971 | 0.199422 | 173 | 0.809624 | -2.19859 | 3.012962 | MLM_2021 |
| DH | Q.CIM.dh.7BL.2 | Excalibur_c25630_537 | 7B | 665679233 | 7.32E-07 | 0.315029 | 173 | 0.001578 | -0.67898 | 6.135337 | FarmCPU_POOL |
| DH | Q.CIM.dh.7BL.3 | BobWhite_c25708_334 | 7B | 727634253 | 0.000494 | 0.141618 | 173 | 0.646916 | -1.36449 | 3.306069 | MLM_POOL |
|  |  |  |  |  | 0.000575 | 0.141618 | 173 | 0.686533 | -1.43472 | 3.240369 | MLM_2020 |

**Table S5:** QTNs in complete linkage across the genome

| **QTNs** | **Chromosome** | **Position1** | **QTLs (Position 2)** | **Chromosome** | **Position2** | **Dist_bp** | **R^2** | **DPrime** | **pDiseq** |
| --- | --- | --- | --- | --- | --- | --- | --- | --- | --- |
| Q.CIM.stb.1AL.8 | 1A | 5.75E+08 | Q.CIM.stb.1AL.7 | 1A | 5.75E+08 | 115 | 1 | 1 | 9.3E-20 |
| Q.CIM.stb.1AL.7 | 1A | 5.75E+08 | Q.CIM.stb.1AL.6 | 1A | 5.75E+08 | 104490 | 1 | 1 | 1.01E-19 |
| Q.CIM.stb.1AL.8 | 1A | 5.75E+08 | Q.CIM.stb.1AL.6 | 1A | 5.75E+08 | 104605 | 1 | 1 | 9.3E-20 |
| Q.CIM.stb_ph.2AL.3 | 2A | 3.82E+08 | Q.CIM.stb.4AL.4 | 1B | 1.49E+08 | N/A | 1 | 1 | 1.6E-43 |
| Q.CIM.stb.3AL.3 | 3A | 5.35E+08 | Q.CIM.stb.3AL.2 | 3A | 5.35E+08 | 934 | 1 | 1 | 1.42E-18 |
| Q.CIM.stb.3AL.4 | 3A | 5.38E+08 | Q.CIM.stb.3AL.3 | 3A | 5.35E+08 | 2313420 | 1 | 1 | 1.32E-18 |
| Q.CIM.stb.3AL.4 | 3A | 5.38E+08 | Q.CIM.stb.3AL.2 | 3A | 5.35E+08 | 2314354 | 1 | 1 | 1.42E-18 |
| Q.CIM.stb.3BL.4 | 3B | 5.41E+08 | Q.CIM.stb.3AL.2 | 3A | 5.35E+08 | N/A | 1 | 1 | 1.98E-17 |
| Q.CIM.stb.3BL.4 | 3B | 5.41E+08 | Q.CIM.stb.3AL.3 | 3A | 5.35E+08 | N/A | 1 | 1 | 1.85E-17 |
| Q.CIM.stb.3BL.4 | 3B | 5.41E+08 | Q.CIM.stb.3AL.4 | 3A | 5.38E+08 | N/A | 1 | 1 | 1.73E-17 |
| Q.CIM.stb.4AL.4 | 4A | 5.99E+08 | Q.CIM.stb.4AL.3 | 4A | 5.99E+08 | 27 | 1 | 1 | 2.17E-26 |
| Q.CIM.stb.4AL.3 | 4A | 5.99E+08 | Q.CIM.stb.4AL.2 | 4A | 5.99E+08 | 734583 | 0.896888 | 1 | 5.01E-24 |
| Q.CIM.stb.4AL.4 | 4A | 5.99E+08 | Q.CIM.stb.4AL.2 | 4A | 5.99E+08 | 734610 | 0.896888 | 1 | 5.01E-24 |
| Q.CIM.stb.4BL.4 | 4B | 6.5E+08 | Q.CIM.stb.4BL.3 | 4B | 6.49E+08 | 132031 | 0.951537 | 1 | 3.58E-27 |
| Q.CIM.stb.4BL.6 | 4B | 6.57E+08 | Q.CIM.stb.4BL.5 | 4B | 6.57E+08 | 268258 | 1 | 1 | 0 |
| Q.CIM.stb.5AL.7 | 5A | 5.73E+08 | Q.CIM.stb.5AL.6 | 5A | 5.72E+08 | 677492 | 0.948951 | 1 | 7.78E-43 |
| Q.CIM.stb.5AL.11 | 5A | 5.89E+08 | Q.CIM.stb.5AL.10 | 5A | 5.87E+08 | 1945657 | 1 | 1 | 6.87E-32 |
| Q.CIM.stb.5AL.8 | 5A | 5.74E+08 | Q.CIM.stb.5AL.7 | 5A | 5.73E+08 | 544965 | 0.80321 | 1 | 2.33E-34 |

**Table S6:** The physical position of reported Stb genes and Meta QTLs

| **Gene/MQTL** | **chr** | **Physical position (bp)** | | **Markers** | **Reference** |
| --- | --- | --- | --- | --- | --- |
| Stb1 | 5B | 402787130 |  | (Xbarc74;) | Adhikari et al. 2004a |
| Stb11 | 1B | 42329007 |  | barc8 | Chartrain et. al., 2005 |
| Stb12 | 4A | 732902997 |  | (Xwmc219;) | Chartrain et al., 2005c |
| Stb13 | 7B | 546855428 |  | (Xwmc396;) | Cowling 2006 |
| Stb14 | 3B | 27530271 |  | (wmc500;wmc632) | Cowling, 2006 |
| Stb16 | 3D | 590040459 |  | - | Saintenac, et al., 2021 |
| Stb17 | 5A | 579827316 |  | (gwm617;hbg291)hbg247, | Ghaffary et al.., 2012 |
| Stb18 | 6D | 21365827 |  | (gpw5176;gpw3087, 26130831 ) | Ghaffary et al., 2011 |
| Stb19 | 1D | 7340804 |  | (1218021, ;4909967, 7420420 ) | Yang et al., 2018 |
| Stb2 | 1B | 42183529 |  | (wmc406;barc8)wmc230 | Liu et al., 2013 |
| Stb3 | 7A | 89970411 |  | TPt-4209 (wPt-1664, wPt-9132, wPt-0864) | Goodwin and Thompson, 2011 |
| Stb4 | 7D | 13460405 |  | gwm111 | Adhikari et al., 2004 |
| Stb6 | 3A | 26200000 |  | - | Saintenac et al., 2018 |
| Stb7 | 4A | 739515101 |  | (Xwmc313;) | McCartney et al. 2003 |
| Stb8 | 7B | 740720573 |  | (Xgwm146;) | Adhikari et al. 2003 |
| Stb9 | 2B | 739396089 |  | (wmc332;gpw1214, 786618055) | Chartrain et. al., 2009 |
| Stbsm3 | 3A | 11685754 |  | (barc321;barc12 ) | Cuthbert, 2011 |
| StbWW | 1B | 53059799 |  | barc119, | Raman et al., 2009 |
| TmStb1 | 7A | 116002861 |  | (Xbarc174;) | Jing et al., 2008 |
| MQTL11 | 2D | 79926878 | 170364558 | (wPt-665644, wPt-0298) wPt-8330 | Goudemand et al, 2013 |
| MQTL13 | 3A | 721223988 | 743563796 | wPt-1036 (wPt-1596, wPt-9761) | Goudemand et al, 2013 |
| MQTL17 | 4B | 549138483 | 579103874 | wPt-1101 (wPt-7365, wPt-6209, wPt-3991) | Goudemand et al, 2013 |
| MQTL20 | 6A | 1252532 | 25798538 | tPt-4209 (wPt-1664, wPt-9132, wPt-0864) | Goudemand et al, 2013 |
| MQTL24 | 7A | 693198258 | 721774214 | (wPt-5524, wPt-0639, wPt-744897) wPt-0971 | Goudemand et al, 2013 |
| MQTL3 | 1B | 632911927 | 632912608 | (wPt-6975) wPt-4721 (wPt-5281) | Goudemand et al, 2013 |
| MQTL4 | 2A | 31987181 | 60709473 | (wPt-740658, wmc177, wPt-9320) wPt-6711 | Goudemand et al, 2013 |
| MQTL5 | 2A | 759323599 | 763485731 | (wPt-9277, wPt-6662, wPt-741584) wPt-7901 | Goudemand et al, 2013 |
| MQTL8 | 2B | 686046185 | 708210455 | (wPt-1646, wPt-0189) wPt-8340 | Goudemand et al, 2013 |
