# Supplementary figures and images for: Identification of resistance sources and genomic regions regulating Septoria tritici blotch resistance in South Asian bread wheat germplasm
Source: Plant Genome. 2024 Nov 27;18(1):e20531. doi: 10.1002/tpg2.20531 (PMC11726422; doi:10.1002/tpg2.20531)

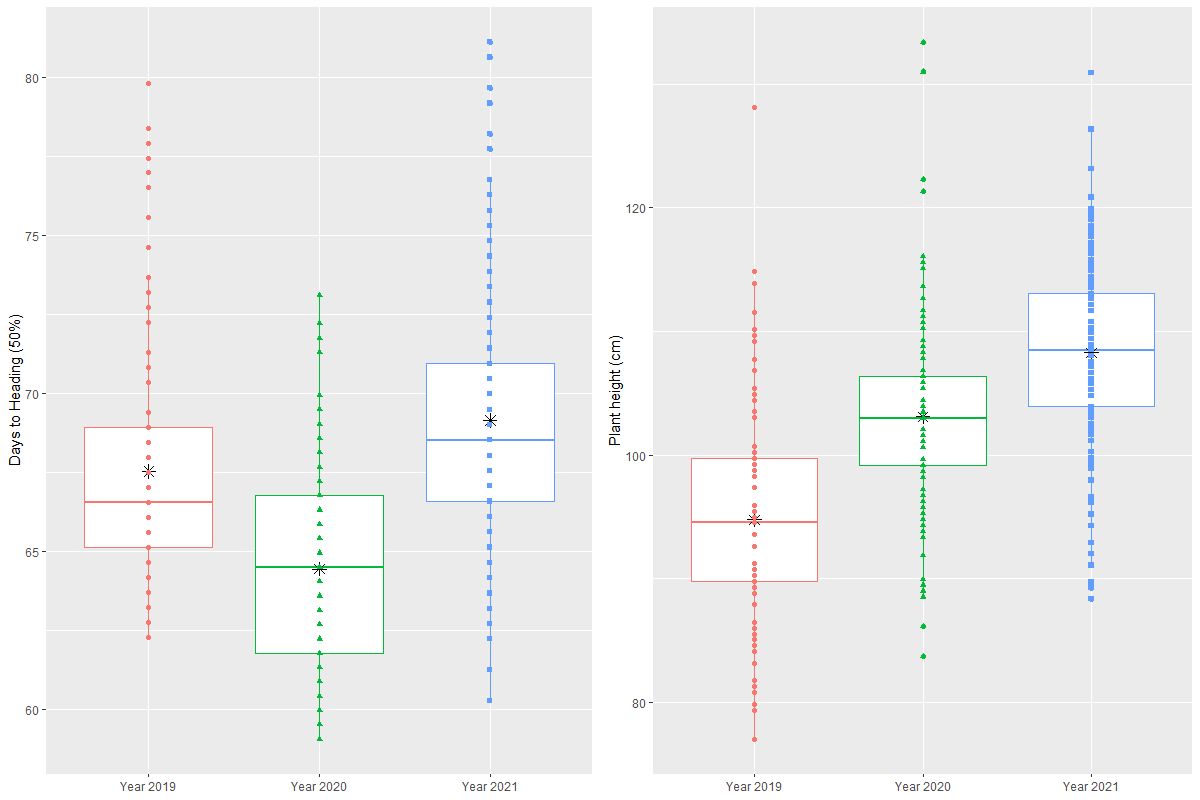

Supplement: Supplementary file 2 — Supporting Information [file TPG2-18-e20531-s001.zip › Supplementary Figures/Fig. S1 Days to heading and Plant height boxplots across the Years.tiff]

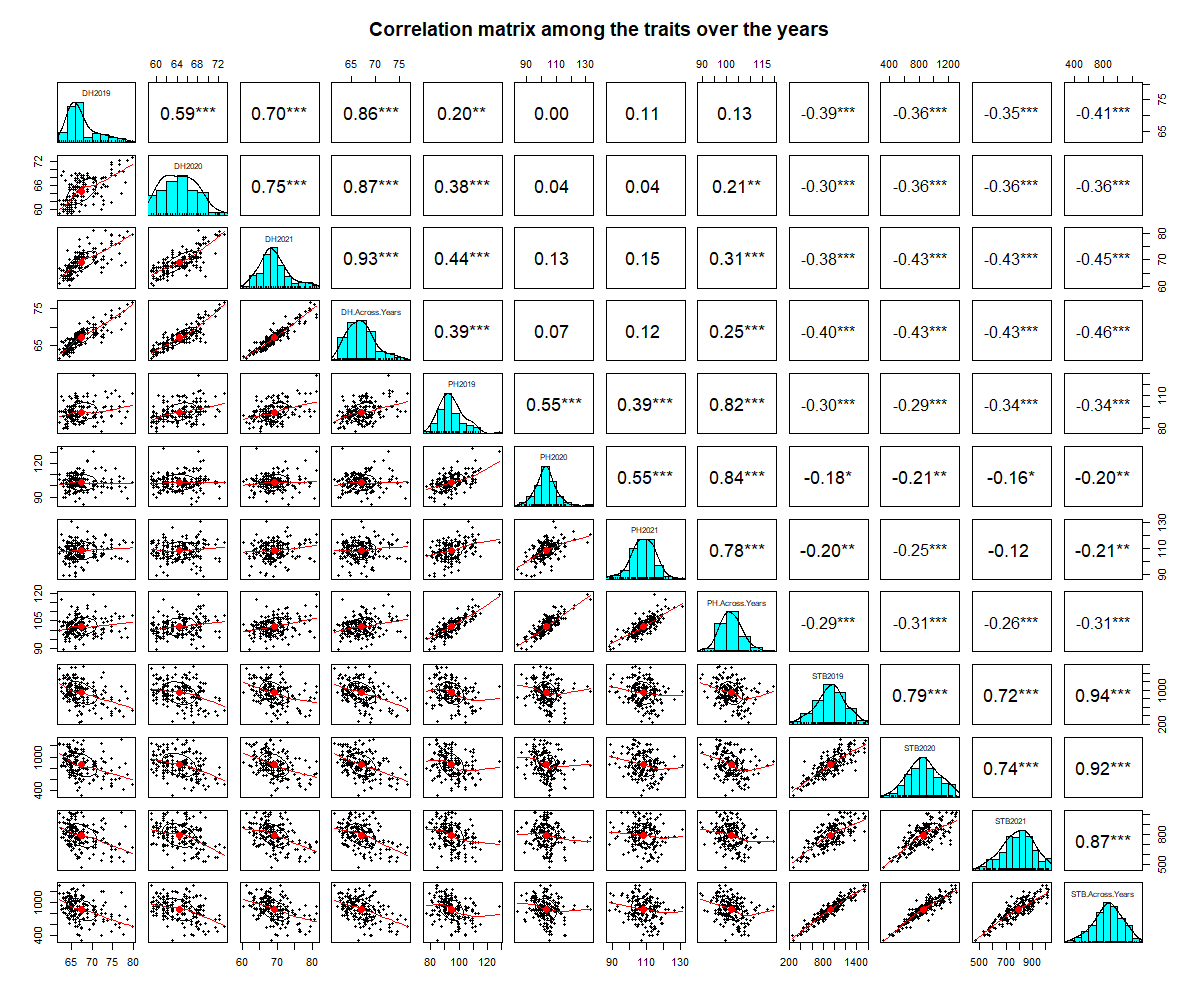

Supplement: Supplementary file 2 — Supporting Information [file TPG2-18-e20531-s001.zip › Supplementary Figures/Fig. S2 Correlation matrix among the Days to heading, plant height and Septoria tritici blotch across the years.tiff]

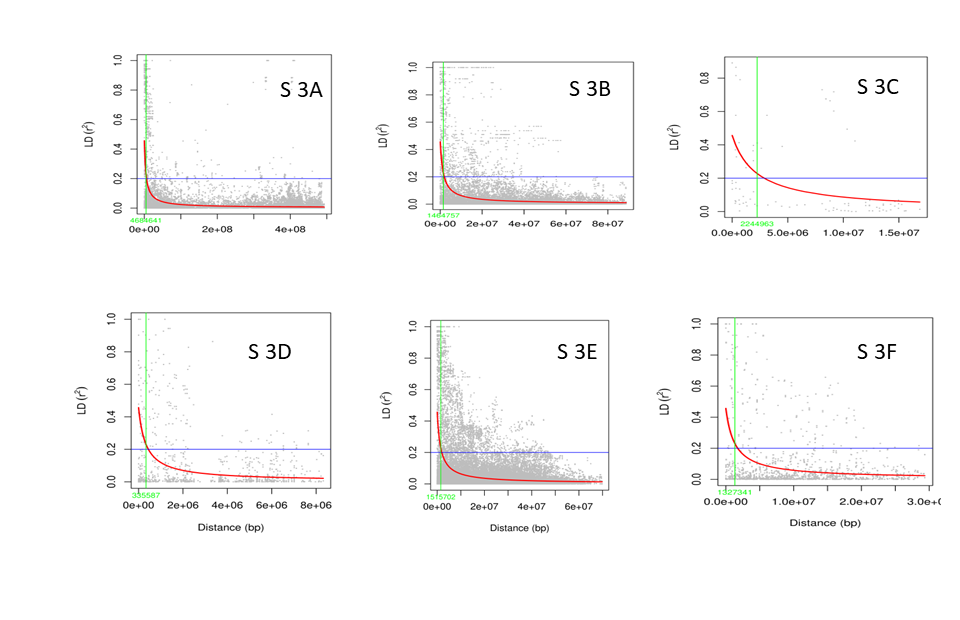

Supplement: Supplementary file 2 — Supporting Information [file TPG2-18-e20531-s001.zip › Supplementary Figures/Fig. S3 LD decay across the genome S 3A) 1D short arm; S 3B) 2B long arm; S 3C) 3B short arm; S 3D) 4A long arm; S 3E) 5A long arm; and S 3F) 7A short arm.TIF]

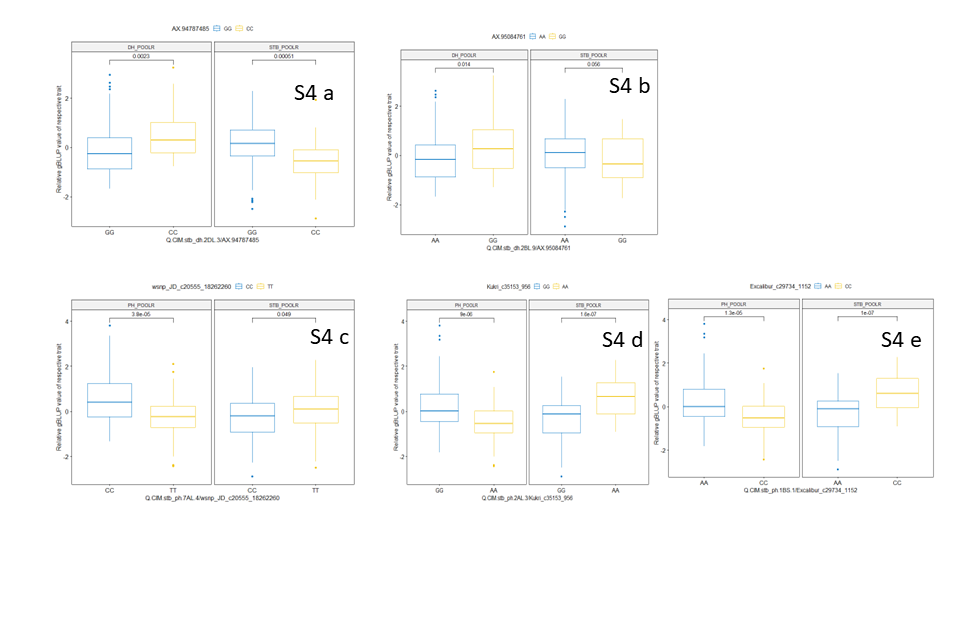

Supplement: Supplementary file 2 — Supporting Information [file TPG2-18-e20531-s001.zip › Supplementary Figures/Fig. S4 Mean differentiating ability of common QTNs for STB, PH and DH.TIF]

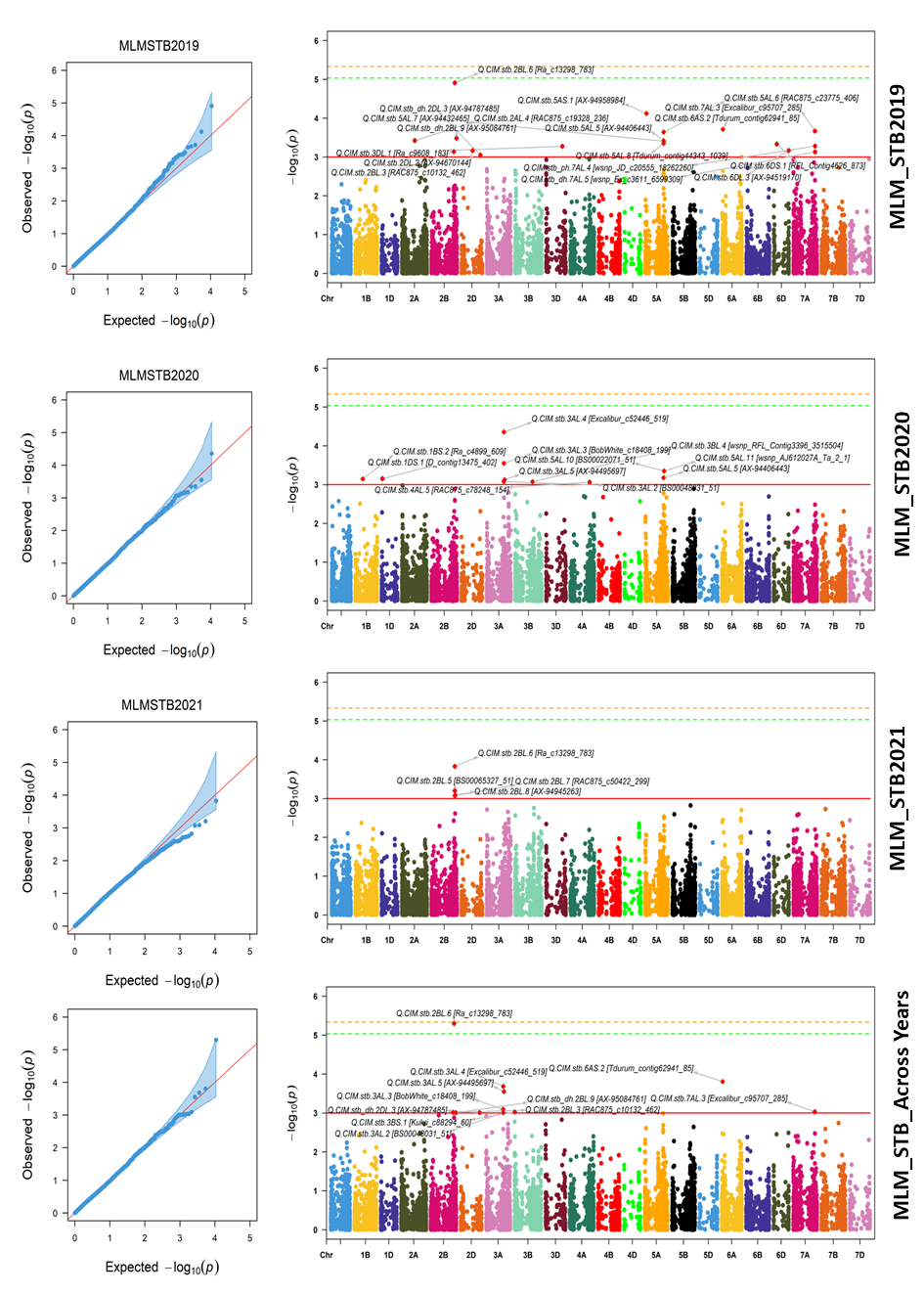

Supplement: Supplementary file 2 — Supporting Information [file TPG2-18-e20531-s001.zip › Supplementary Figures/Fig. S5. Manhattan plot with MLM for Septoria tritici blotch.TIF]
